# The environmental degradation of naphthalimide, rhodamine and BODIPY fluorophores by hydroxyl radicals: a theoretical insight

**DOI:** 10.1039/d6ra00906a

**Published:** 2026-03-05

**Authors:** Nguyen Linh Nam, Mai Van Bay, Nguyen Thi Hoa, Nguyen Quang Trung, Nguyen Minh Thong, Adam Mechler, Pham Cam Nam, Nguyen Khoa Hien, Duong Tuan Quang, Quan V. Vo

**Affiliations:** a The University of Danang – University of Technology and Education Danang 550000 Vietnam vvquan@ute.udn.vn; b The University of Danang – University of Sciences and Education Danang 550000 Vietnam; c Quality Assurance and Testing Center 2 Da Nang 550000 Vietnam; d Department of Biochemistry and Chemistry, La Trobe University Victoria 3086 Australia; e The University of Danang – University of Technology and Sciences Danang 550000 Vietnam; f Mientrung Institute for Scientific Research, Vietnam National Museum of Nature, Vietnam Academy of Science and Technology Hue 530000 Vietnam; g Department of Chemistry, Hue University Hue 530000 Vietnam

## Abstract

Rhodamine (RDA), naphthalimide (NPA), and BODIPY (BOD) are widely used fluorophores whose environmental fate is hitherto unexplored. In this study, quantum chemical calculations were employed to investigate the HO˙-driven degradation processes of these fluorophores in aqueous and organic media, focusing on the mechanism, kinetics, thermodynamics, and ecological risks. The results suggest that all three fluorophores undergo reactions with HO˙, but their behaviors differ markedly. RDA displays strong pH-dependent reactivity, with overall rate constants ranging from 8.76 × 10^8^ to 4.02 × 10^10^ M^−1^ s^−1^ depending on protonation state, and lifetimes spanning from hours to years in natural waters. NPA degrades more slowly, with rate constants of 7.06 × 10^7^ M^−1^ s^−1^ (neutral form) and 8.20 × 10^8^ M^−1^ s^−1^ (anion), resulting in greater environmental persistence. BOD reacts rapidly across all conditions (4.39 × 10^9^ M^−1^ s^−1^) consistently *via* RAF mechanism. Temperature and solvent polarity also influence degradation: higher temperatures accelerate all reactions, and methanol enhances reactivity, while lipid-like media reduce degradation rates for RDA and NPA but not for BOD. NPA, RAD and these intermediates are predicted to exhibit high ecological toxicity (log LC_50_/EC_50_/ChV < 2), poor biodegradability, and high bioaccumulation potential, while BOD and its products may be comparatively benign. Overall, the hazard ranking is predicted to be RDA ≈ NPA > BOD, with RDA posing the highest long-term ecological risk. These findings highlight the need to evaluate not only degradation efficiency but also the toxicity and environmental fate of intermediates when applying advanced oxidation processes for fluorophore removal.

## Abstract 2

In the environment, all three fluorophores undergo reactions with HO˙, but their behaviors differ markedly.

## Graphical abstract

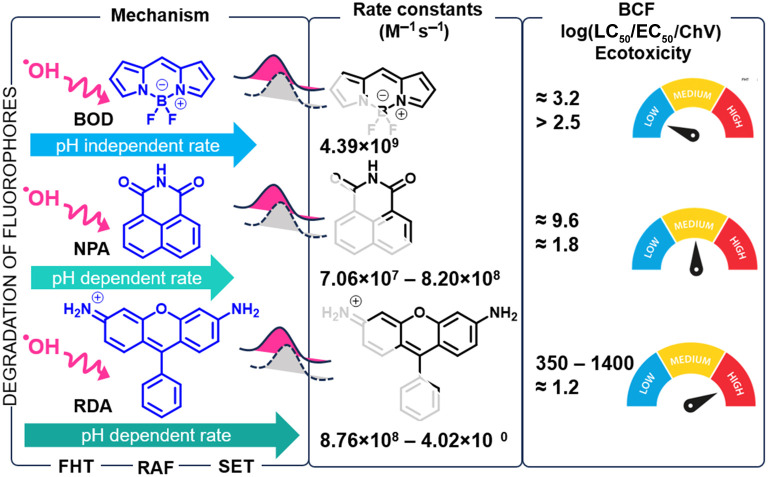

## 1. Introduction

Fluorophores (FPHs) are widely employed in modern chemical, biological, and environmental research principally as labels for observation or sensing.^1–3^ Among them, rhodamine (RDA),^4–6^ naphthalimide (NPA),^7–9^ and BODIPY (BOD)^10–14^ are the most important scaffolds that are also frequently derivatized to tune their photophysical properties. Consistently, these scaffolds are frequently used in the design of fluorescent sensors for detecting ions, biomolecules, and environmental pollutants owing to their strong absorption, tunable emission, and chemical versatility.^15,16^ Increasing production and use of these compounds have led to the inevitable release of organic contaminants containing RDA, NPA, and BOD motifs into aquatic and terrestrial environments.

Hydroxyl radicals (HO˙) are the key transient oxidants in natural surface waters and aquifers due to their exceptionally high reactivity toward organic substrates.^17,18^ Consistently their steady-state concentrations are extremely low (typically 10^−18^ to 10^−15^ M), also influenced by temperature, pH, and matrix constituents such as dissolved organic matter (DOM), carbonate/bicarbonate, and halides that can act as radical scavengers or secondary radical sources.^19–21^ Due to its high reactivity HO˙ is consumed at the site of its generation through photochemical processes, and that is the main reason why, even at very low steady state concentrations, HO˙ plays a decisive role in controlling the environmental fate of biomolecules including industrial chemicals, underpinning the natural self-purification of water.

In contrast, engineered advanced oxidation processes (AOPs) intentionally generate substantially higher transient concentrations of reactive oxygen species (ROS), including HO˙, SO_4_˙^−^, Cl˙, ClO˙, HOO˙ and O_2_˙^−^ to enhance contaminant degradation. Among these, HO˙ is also regarded as the predominant oxidant responsible for organic transformation.^22–24^ Through single-electron transfer, hydrogen abstraction, and radical addition mechanisms, HO˙ can initiate complex degradation pathways that may convert relatively benign fluorophores into more reactive or potentially toxic intermediates. Therefore, a comprehensive environmental safety assessment requires not only evaluation of the parent compounds but also a mechanistic understanding of their HO˙-induced degradation pathways and products under clearly defined environmental and treatment scenarios.

**Fig. 1:**
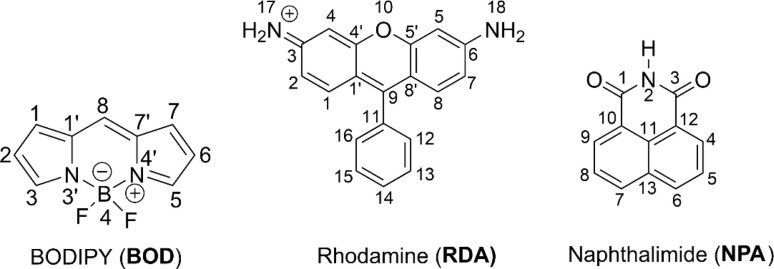
Structure of fluorophores (FPHs).

To date, however, systematic studies on the degradation mechanisms and ecological toxicity of the intermediates derived from RDA, NPA, and BOD (Fig. 1) have not been reported. This knowledge gap is critical, as these fluorophores are structurally diverse and may exhibit different degradation behaviors, reactivities, and risks. Comprehensive mechanistic insights are therefore needed to assess both their environmental persistence and the potential ecological hazards of their transformation products.

In recent years, computational chemistry has reached a level of predictive accuracy that allows its use as a tool for exploring experimentally challenging chemical processes, such as environmental degradation pathways,^25–30^ delivering reliable predictions of thermodynamics, kinetics, as well as toxicity of the byproducts. In this study, we employed state-of-the-art computational approaches to investigate the degradation of RDA, NPA, and BOD by hydroxyl radicals in aqueous solution, as well as their relevance to sensor applications in biological and lipid-like environments, with a particular focus on elucidating their mechanistic pathways and assessing the toxicity of major intermediates. This work provides the first systematic evaluation of these important fluorophores, linking molecular reactivity with environmental risk.

## 2. Computational methods

All calculations were performed using Gaussian 16 at the M062X/6-311++G(d,p) level of theory.^31^ This approach has been demonstrated to provide reliable thermodynamic and kinetic parameters for radical reactions.^32–38^ Solvent effects in water were described using the SMD implicit solvation model,^32^ which is widely applied in studies of radical reactions. The protocol has been validated against experimental data, typically affording *k*_calc._/*k*_exp._ ratios between 0.3 and 2.9.^34,39–43^ Although improved treatments of intramolecular rotational contributions have been suggested,^44^ the Quantum Mechanics-based Overall Free Radical Scavenging Activity (QM-ORSA) method^43^ was adopted to balance computational cost and accuracy for the present system. Accordingly, kinetic parameters were determined within the QM-ORSA method.^34,43,45^ Under standard conditions of 1 M concentration and varying ambient temperatures (273–373 K for water), the rate constant (*k*) was determined using eqn (1) and transition state theory (TST) and details in Table S1, SI.^41,46–51^1
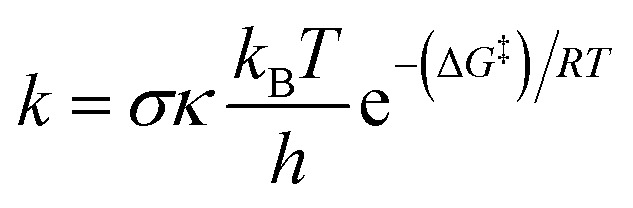


The Gibbs free energy of activation is denoted as Δ*G*^‡^, while *k*_B_ and *h* represent the Boltzmann constant and Planck constant, respectively. Tunneling corrections (*κ*), were computed using the Eckart barrier model.^52^*σ* stands for the reaction symmetry number.^53,54^

The radical adduct formation (RAF), single electron transfer (SET), or formal hydrogen transfer (FHT) pathways detailed in eqn (2)–(4) can provide the mechanistic underpinning of the reaction between FPHs and HO˙, with consideration to the molecular structure:^34,41,45,55–58^

2 RAF: FPHs-H + HO˙ → [HO-FPHs-H]˙

3 SET: FPHs-H + HO˙ → [FPHs-H]˙^+^ + HO^−^

4 FHT: FPHs-H + HO˙ → FPHs˙ + H_2_O

The ecotoxicological potential was predicted using the Ecological Structure–Activity Relationship model (ECOSAR V2.0), which has been extensively validated for assessing the aquatic toxicity of organic compounds.^59–63^ Bioconcentration factors (BCFs) of the transformation products derived from FPH degradation were estimated with the BCFBAF module implemented in the EPI Suite™ package.^64^ In addition, the biodegradation behavior of FPHs and their corresponding degradation products was evaluated using the BIOWIN 3, 4, and 5 sub-models available within EPI Suite™.^64^ For computational consistency, radical intermediates were converted into their hydrogen-saturated closed-shell forms prior to prediction.

## 3. Results and discussion

### 3.1. The reaction of fluorophores with HO˙ in water

#### 3.1.1. Deprotonation

**Fig. 2:**
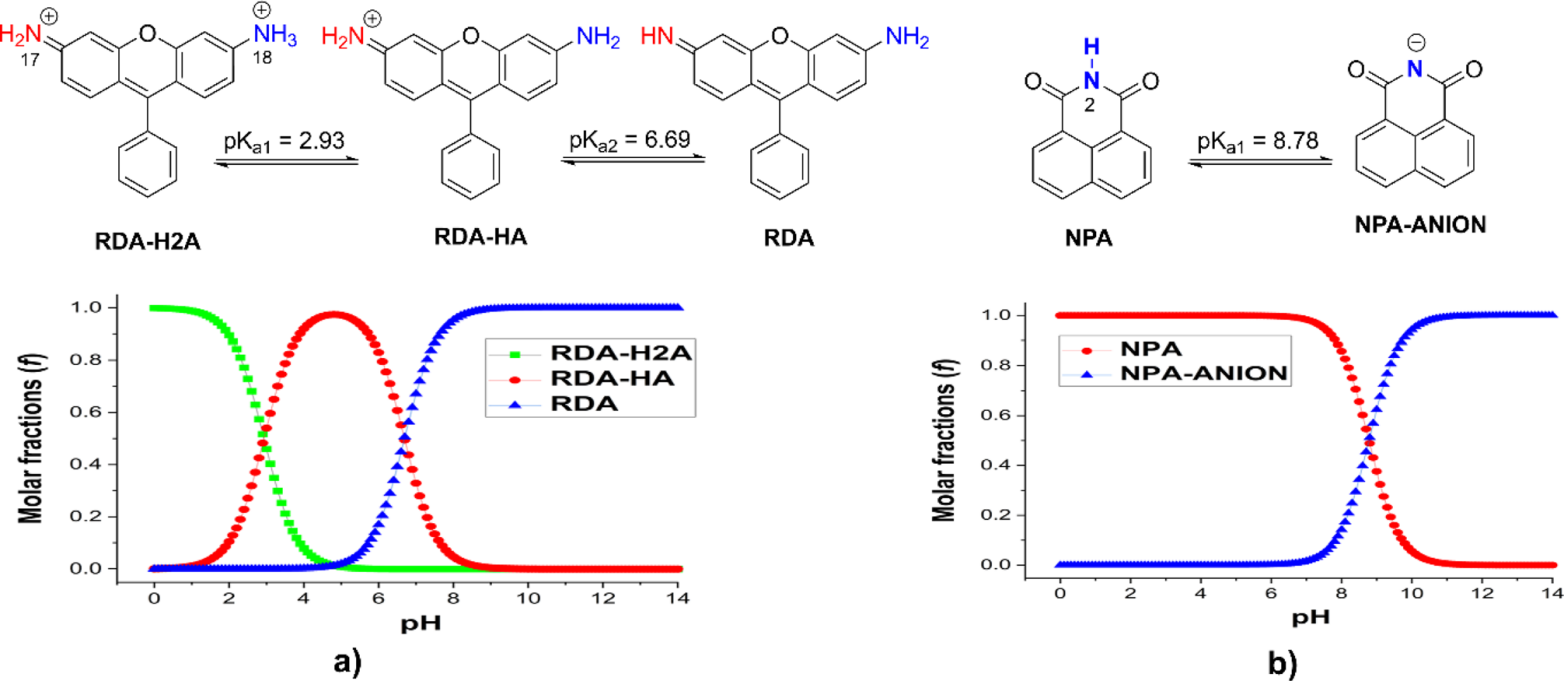
The deprotonation and molar fractions of RDA (a) and NPA (b) (the results were obtained *via* the Chemaxon code).^65^

In natural aquatic systems, the protonation equilibria of RDA and NPA determine their speciation profiles and strongly influence their reactivity toward hydroxyl radicals. For RDA, the p*K*_a_ values (p*K*_a1_ = 2.93; p*K*_a2_ = 6.69) indicate two successive deprotonation steps (Fig. 2a). At acidic pH (<3), RDA-H_2_A is the dominant species. In the pH = 3–6, the equilibrium shifts toward the RDA-HA form, which becomes the major contributor. Under alkaline conditions (pH ≥ 7), the neutral RDA form progressively dominates, reaching over 95% at pH 8. This pH-dependent distribution highlights that RDA will mostly exist as the neutral form at pH > 8, which modifies its solvation and electronic distribution and thus its susceptibility to radical attack.

By contrast, NPA, with a single weakly acidic site (p*K*_a_ = 8.78), remains almost entirely in the neutral state throughout acidic to near-neutral pH (Fig. 2b). Only under mildly alkaline conditions does the anionic species emerge, becoming significant around its p*K*_a_. Thus, unlike RDA, NPA shows little speciation change in natural waters, and its reactivity toward hydroxyl radicals will be governed largely by its intrinsic neutral electronic structure. The results show that BOD does not undergo proton dissociation and therefore maintains a constant molecular form across the environmental pH range. Compared with RDA and NPA, this absence of speciation effects implies that its degradation kinetics are independent of pH and controlled mainly by inherent electronic properties.

Accordingly, RDA is highly sensitive to environmental pH through its stepwise deprotonation equilibria, while NPA and BOD remain largely invariant under neutral conditions. This difference provides a clear mechanistic basis for the distinct environmental degradation pathways expected for these fluorophores. Thus, the degradation processes of RAD and NPA in aqueous solution will be evaluated under their deprotonated forms, whereas that of BOD will be excluded from consideration.

#### 3.1.2. Thermal evaluation

The calculated Δ*G*° (kcal mol^−1^) of the reactions between FPHs and HO˙ radical *via* the formal hydrogen transfer (FHT), radical adduct formation (RAF), and single electron transfer (SET) pathways at 298.15 K in water are summarized in Table 1. These data provide a thermodynamic basis for assessing the dominant degradation mechanisms of the studied fluorophores under environmentally relevant conditions.

**Table 1:** The calculated Δ*G*° (kcal mol^−1^) of the reaction between FPHs with HO˙ radical following the FHT, RAF and SET pathways at 298.15 K in water

Mechanisms	Positions	BOD	RDA-H_2_A	RDA-HA	RDA	NPA	NPA-ANION
FHT	B–F	70.9					
	N2–H					0.0	
	N17–H		−13.9	−19.9	−28.4		
	N18–H		−35.6	−19.9			
RAF	C1	−26.6	−13.3	−9.7	−15.3		6.7
	C1′	−10.3	−4.9	3.1	−11.4		
	C2	−14.3	−17.8	−13.3	−22.9		
	C3	−32.2	0.5	−4.4	−5.3		
	C4		−13.1	−8.9	−19.7	−12.4	−12.5
	C4′		−10.7	−6.5	−16.7		
	C5	−32.4	−10.9	−8.3	−10.0	−7.3	−8.0
	C5′		−12.2	−6.3	−18.6		
	C6	−14.4	−16.4	−4.2	−12.9	−17.0	−17.8
	C7	−26.2	−7.0	−12.8	−10.3	−17.0	−17.8
	C7′	−10.4			−10.9		
	C8	−9.0	−13.4	−10.2	−0.4	−7.3	−8.0
	C8′		6.0	0.4	−15.3		
	C9		−23.3	−15.4	−31.6	−12.4	−12.5
	C10					−4.8	−7.6
	C11		−4.9	−0.4	−0.4	9.0	8.7
	C12		−6.9	−6.6	−6.6	−4.8	−7.6
	C13		−5.8	−5.9	−5.9	10.1	9.6
	C14		−8.7	−7.6	−7.4		
	C15		−5.8	−6.0	−6.0		
	C16		−6.9	−6.7	−6.7		
	N3′	26.8					
	N4′	27.0					
	N17		12.4	19.7	29.6		
	N18			19.6	−10.6		
SET		8.8	24.3	9.7	−9.5	25.0	13.6

For RDA, the protonation state strongly modulates the reaction thermodynamics. In the diprotonated (RDA-H_2_A) and monoprotonated (RDA-HA) forms, FHT at N–H bonds are highly exergonic, with Δ*G*° values down from −13.9 to −35.6 kcal mol^−1^. In neutral form (RDA), FHT at N17–H remains strongly favorable (Δ*G*° = −28.4 kcal mol^−1^), while RAF at conjugated carbon centers such as C9 (Δ*G*° = −31.6 kcal mol^−1^) and C2 (Δ*G*° = −22.9 kcal mol^−1^) also becomes competitive, consistent with the dual H-abstraction/addition accessibility seen in aromatic compounds.^30,66,67^ RAF at nitrogen sites, however, is endergonic (positive Δ*G*°), supporting the role of N atoms as H-donors rather than radical-addition sites.

NPA exhibits a markedly different profile. For the neutral form, the Gibbs free energy of FHT(N2–H) is nearly zero, suggesting a limited driving force for hydrogen abstraction. Instead, RAF provides the dominant route, with favorable addition at C6/C7 (Δ*G*° = −17.0 kcal mol^−1^) and C4/C9 (Δ*G*° = −12.4 kcal mol^−1^). Deprotonation to the NPA-ANION slightly enhances the exergonicity of RAF, whereas SET remains energetically unfavorable (Δ*G*° = 13.6 kcal mol^−1^). Such carbon-centered RAF as the major degradation route parallels findings for oxidation of phenols and aromatic amino acids by ˙OH.^68,69^ However, the thermodynamic profile is markedly different in the BOD. FHT is strongly unfavorable (Δ*G*° = 70.9 kcal mol^−1^) and SET is marginally accessible (Δ*G*° = 8.8 kcal mol^−1^). In contrast, the RAF pathway at several aromatic carbons is highly exergonic. Specifically, the calculated Δ*G*° values are −32.4 kcal mol^−1^ at C5 and −32.2 kcal mol^−1^ at C3, indicating that RAF is the dominant thermodynamically favored pathway. This agrees with computational studies on methyl *N*-(3,4-dichlorophenyl)carbamate and oxcarbazepine degradation, where radical addition at electron-rich aromatic carbons drives degradation.^28,30^

Thus, the findings reveal distinct thermodynamic characteristics among the studied compounds. RDA can undergo either FHT or RAF mechanisms depending on its protonation state, whereas NPA proceeds *via* both RAF and FHT mechanisms. In contrast, BOD is predominantly degraded through the RAF pathway. The consistently unfavorable SET process indicates its minor role in aqueous media, apart from the NPA-ANION state. These thermodynamic insights establish a mechanistic basis for assessing and predicting the environmental degradation behaviors of fluorophores under hydroxyl radical attack.

#### 3.1.3. Kinetic evaluation

**Fig. 3:**
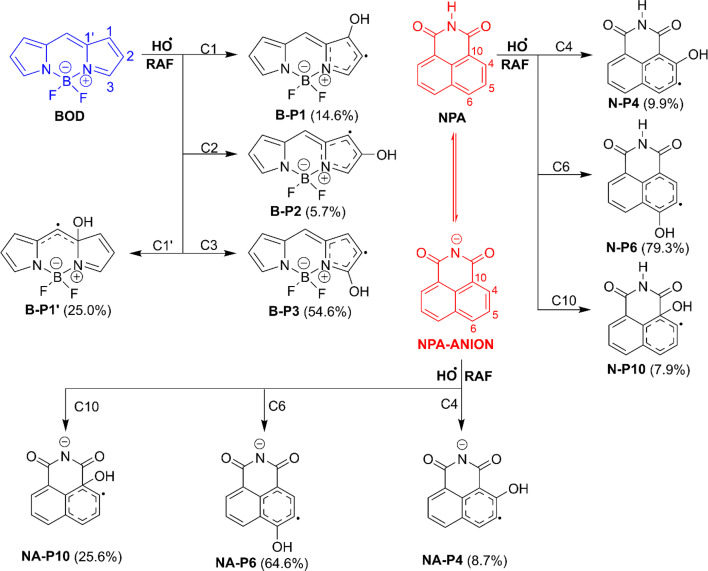
The dominant mechanisms and % products (*Γ* > 5%) of the HO˙ + BOD/NPA reactions in water at 298.15 K.

**Fig. 4:**
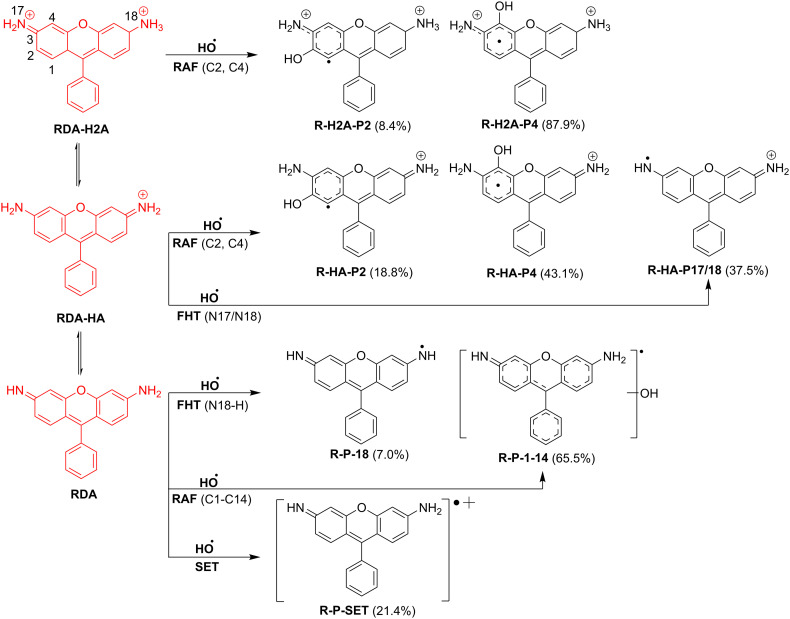
The dominant mechanisms and % products (*Γ* > 5%) of the HO˙ + RDA reactions in water at 298.15 K.

The kinetic analysis of hydroxyl radical attack on the studied fluorophores in aqueous solution reveals strong mechanistic dependence on both molecular structure and protonation state. The detailed data, including activation free energies (Δ*G*^‡^), tunneling corrections (*k*), branching ratios (*Γ*), and rate constants (*k*_Eck_ and *k*_overall_), are presented in Table 2 for BOD/NPA and Table 3 for RDA. The corresponding dominant pathways and their relative product distributions are illustrated in Fig. 3 (BOD/NPA) and Fig. 4 (RDA).

**Table 2:** Calculated Δ*G*^‡^ (kcal mol^−1^), rate constants (*k*_Eck_ and *k*_overall_ M^−1^ s^−1^), *k* and *Γ* of the reaction between HO˙ and BOD/NPA reactions at 298.15 K in water

Mechanisms		BOD			NPA					
		Δ*G*^‡^	*k*_app_	*Γ*	NPA			NPA-ANION		
					Δ*G*^‡^	*k*_app_	*Γ*	Δ*G*^‡^	*k*_app_	*Γ*
FHT	N2–H				25.2	2.00 × 10^−6^	0.0			
RAF	C1	5.8	6.40 × 10^8^	14.6						
	C1′	5.2	1.10 × 10^9^	25.0						
	C2	6.4	2.50 × 10^8^	5.7						
	C3	3.7	2.40 × 10^9^	54.6						
	C4				8.7	7.00 × 10^6^	9.9	7.2	7.10 × 10^7^	8.7
	C5				9.4	2.00 × 10^6^	2.8	5.9	5.30 × 10^8^	1.1
	C6				7.4	5.60 × 10^7^	79.3	6.5	2.10 × 10^8^	64.6
	C8	8.6	3.20 × 10^6^	0.1						0.0
	C10				8.3	5.60 × 10^6^	7.9	6.5	2.10 × 10^8^	25.6
***k***_**overall**_			**4.39 × 10** ^**9**^			**7.06 × 10** ^**7**^			**8.20 × 10** ^**8**^	

**Table 3:** Calculated Δ*G*^‡^ (kcal mol^−1^), rate constants (*k*_app_ and *k*_overall_ M^−1^ s^−1^), *k* and *Γ* of the reaction between HO˙ and RDA reactions at 298.15 K in water

Mechanism		RDA-H_2_A			RDA-HA			RDA		
		Δ*G*^‡^	*k*_app_	*Γ* (%)	Δ*G*^‡^	*k*_app_	*Γ* (%)	Δ*G*^‡^	*k*_app_	*Γ* (%)
SET								1.0	8.50 × 10^9^	21.2
FHT	N17–H	20.6	9.90 × 10^−3^	0.0	4.9	2.00 × 10^9^	37.5			0.0
	N18–H	25.0	2.50 × 10^1^	0.0				≈0	2.80 × 10^9^	7.0
RAF	C1	8.8	2.70 × 10^6^	0.3	9.1	3.40 × 10^6^	0.1	5.2	7.20 × 10^8^	1.8
	C1′	10.7	1.10 × 10^5^	0.0				1.3	2.60 × 10^9^	6.5
	C2	6.8	7.40 × 10^7^	8.4	5.3	1.00 × 10^9^	18.8	2.6	2.60 × 10^9^	6.5
	C3	13.2	1.40 × 10^3^	0.0	11.4	6.80 × 10^4^	0.0	9.4	9.00 × 10^5^	0.0
	C4	5.1	7.70 × 10^8^	87.9	3.7	2.30 × 10^9^	43.1	≈0	2.80 × 10^9^	7.0
	C4′	10.2	2.50 × 10^5^	0.0	11.4	7.10 × 10^4^	0.0	2.5	2.30 × 10^9^	5.7
	C5	7.9	1.30 × 10^7^	1.5				1.9	2.70 × 10^9^	6.7
	C5′	11.7	2.50 × 10^4^	0.0				2.2	2.30 × 10^9^	5.7
	C6	10.3	2.60 × 10^5^	0.0				3.8	2.60 × 10^9^	6.5
	C7	9.6	7.50 × 10^5^	0.1				3.0	2.20 × 10^9^	5.5
	C8	9.4	1.20 × 10^6^	0.1				6.0	2.50 × 10^8^	0.6
	C8′	14.5	1.90 × 10^2^	0.0				2.1	2.60 × 10^9^	6.5
	C9	11.0	7.00 × 10^4^	0.0	11.8	1.70 × 10^4^	0.0	0.3	2.60 × 10^9^	6.5
	C11	16.1	1.30 × 10^1^	0.0	16.1	1.20 × 10^1^	0.0	15.9	1.80 × 10^1^	0.0
	C12	8.4	1.10 × 10^7^	1.3	7.9	2.40 × 10^7^	0.5	7.9	1.20 × 10^7^	0.0
	C13	9.5	1.60 × 10^6^	0.2	9.2	2.60 × 10^6^	0.0	23.4	2.20 × 10^−15^	0.0
	C14	9.4	1.00 × 10^6^	0.1	9.1	1.50 × 10^6^	0.0	8.5	3.90 × 10^6^	0.0
	N17							1.5	2.60 × 10^9^	6.5
***k***_**overall**_			**8.76 × 10** ^**8**^			**5.33 × 10** ^**9**^			**4.02 × 10** ^**10**^	

The BOD + HO˙ reaction only occurred following the RAF mechanism. The major pathway was C3 addition forming B-P3 (Δ*G*^‡^ = 3.7 kcal mol^−1^, *Γ* = 54.6%, *k* = 2.40 × 10^9^ M^−1^ s^−1^, Fig. 3). Significant secondary products included B-P1′ (Δ*G*^‡^ = 5.2 kcal mol^−1^, *Γ* = 25%, *k* = 1.10 × 10^9^ M^−1^ s^−1^), B-P1 (Δ*G*^‡^ = 5.8 kcal mol^−1^, *Γ* = 14.6%, *k* = 6.40 × 10^8^ M^−1^ s^−1^), and B-P2 (Δ*G*^‡^ = 6.4 kcal mol^−1^, *Γ* = 5.7%, *k* = 2.50 × 10^8^ M^−1^ s^−1^). With *k*_overall_ = 4.39 × 10^9^ M^−1^ s^−1^, BOD was consumed rapidly by HO˙ despite the absence of any contributions of the FHT and SET reactions.

Studying the degradation of NPA indicated that the neutral form reacted moderately with HO˙, characterized by a dominant RAF pathway at C6 forming N-P6 (Δ*G*^‡^ = 7.4 kcal mol^−1^, *Γ* = 79.3%, *k* = 5.60 × 10^7^ M^−1^ s^−1^, Fig. 3) and minor contributions from N-P4 (C4 addition, Δ*G*^‡^ = 8.7 kcal mol^−1^, *Γ* = 9.9%, *k* = 7.00 × 10^6^ M^−1^ s^−1^) and N-P10 (C10 addition, Δ*G*^‡^ = 8.3 kcal mol^−1^, *Γ* = 7.9%, *k* = 5.60 × 10^6^ M^−1^ s^−1^). Its overall rate constant (7.06 × 10^7^ M^−1^ s^−1^) was below the fast degradation threshold, indicating medium reactivity and comparatively higher environmental persistence. Deprotonation to the NPA-ANION, however, increased the reactivity substantially, raising the overall rate to 8.20 × 10^8^ M^−1^ s^−1^. The RAF reaction was still favored for the anion state with a broader distribution (N-P6, *Γ* = 64.6%, *k* = 2.10 × 10^8^ M^−1^ s^−1^) and remained dominant, while N-P10 (*Γ* = 25.6%, *k* = 2.10 × 10^8^ M^−1^ s^−1^) became a significant secondary pathway.

As presented in Table 3, the overall rate constant for the RDA-H_2_A + HO˙ reaction was the lowest, at 8.76 × 10^8^ M^−1^ s^−1^, while that for the RDA + HO˙ reaction was the highest, reaching 4.02 × 10^10^ M^−1^ s^−1^. The RDA-HA + HO˙ reaction exhibited an intermediate reactivity, with an overall rate constant of 5.33 × 10^9^ M^−1^ s^−1^.

The most favorable pathway of the RDA-H_2_A + HO˙ reaction was RAF at C4, yielding the intermediate R-H_2_A-P4 (*k* = 7.70 × 10^8^ M^−1^ s^−1^, *Γ* = 87.9%, Fig. 4). A secondary pathway at C2 (Δ*G*^‡^ = 6.8 kcal mol^−1^) produced R-H_2_A-P2 (*Γ* = 8.4%, *k* = 7.40 × 10^7^ M^−1^ s^−1^). In contrast, the RDA-HA + HO˙ reaction was defined by both the FHT and RAF reactions. The FHT at N–H (Δ*G*^‡^ = 4.9 kcal mol^−1^) lead to R-HA-P17/18 (*Γ* = 37.5%, *k* = 2.00 × 10^9^ M^−1^ s^−1^), whereas the RAF at C4 (Δ*G*^‡^ = 3.7 kcal mol^−1^) yielded R-HA-P4 (*Γ* = 43.1%, *k* = 2.30 × 10^9^ M^−1^ s^−1^) and at C2 (Δ*G*^‡^ = 5.3 kcal mol^−1^) formed R-HA-P2 (*Γ* = 18.8%, *k* = 1.00 × 10^9^ M^−1^ s^−1^). Unlike the protonated species, neutral RDA distributed reactivity across a broad set of RAF pathways, each contributing between 1.8 and 7.0% to the branching ratio with *k* = 7.20 × 10^8^ to 2.80 × 10^9^ M^−1^ s^−1^. In addition, the SET pathway produced R-P-SET with a substantial contribution (*Γ* = 21.2%, *k* = 8.50 × 10^9^ M^−1^ s^−1^). This mechanistic expansion underscored the influence of deprotonation, which opened multiple parallel decay routes and significantly accelerated degradation.

The results show that protonation state governed the degradation mechanisms. The defining mechanism of the RDA degradation shifted from selective RAF to mixed FHT/RAF and finally to RAF/SET with deprotonation. NPA remained RAF-controlled but became more reactive, and BOD was also consistently RAF-dominated. Overall, deprotonation enhanced reactivity and influenced fluorophore stability under hydroxyl radical attack.

#### 3.1.4. The effects of pH on the degradation

**Fig. 5:**
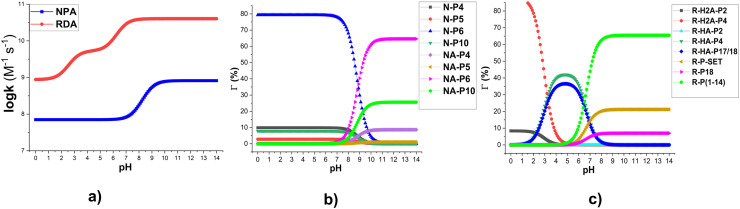
The log(*k*_overall_) (a) and the branching ratios (*Γ*, %) of the main products of the NPA (b)/RDA (c) + HO˙ reactions in water following the pH values at 298.15 K.

The degradation of RDA and NPA by hydroxyl radicals shows a marked dependence on pH, while BOD remains unaffected since it does not undergo proton dissociation. The molar fractions of the individual species were calculated using conventional acid–base equilibrium expressions. While uncertainties in the reported p*K*_a_ values may moderately affect the predicted species distributions and log(*k*_overall_), such deviations are expected to impose limited and approximately symmetric influences, and therefore do not materially alter the overall kinetic trends or mechanistic conclusions. Fig. 5a illustrates the pH–log(*k*_overall_) relationships, and Fig. 5b and c depict the evolution of branching ratios (*Γ*, %) of the main intermediates as a function of pH.

It was observed that proton dissociation exerts a pronounced influence on the reactivity of RDA. At pH values below 2, where the diprotonated form (RDA-H_2_A) predominated, the overall rate constant remained relatively low (log(*k*_overall_) = 8.9–9.1). However, as the pH increased to the range of 2.0–8.7, the log(*k*_overall_) rose by approximately 1.5 units, subsequently stabilizing at 10.6 for pH > 8.7.

This steep rise reflects the combined effect of expanded RAF channels and the additional SET contribution available in the neutral form. Concomitantly, the branching ratios changed from a highly selective RAF pathway at C4 (R-H_2_A-P4, Fig. 5c) in acidic solution, to a mixed FHT/RAF reaction (R-HA-P17/18, R-HA-P4, R-HA-P2) under mildly acidic–neutral conditions, and finally to a diversified RAF and SET reactions at higher pH, with multiple intermediates (R-P-SET, R-P-18, R-P-(1–14)) contributing more than 5%. Thus, higher pH values favored both faster overall degradation and a broader distribution of intermediate products.

For NPA, the neutral form (NPA-HA) dominated under acidic to neutral conditions, yielding log(*k*_overall_) = 7.8 and moderate reactivity. In this condition, RAF at C6 (N-P6) was the dominant product (>79%, Fig. 5b), with minor contributions from C4 (N-P4) and C10 (N-P10). As the pH increased toward alkaline conditions, deprotonation occurred, forming the NPA-ANION. This transition substantially accelerated the reaction, with log(*k*_overall_) rising above 8.9 and entering the fast-degradation domain. The product distribution also broadened: while NA-P6 remained the major intermediate (≈65%), NA-P10 increased to ≈25%, and NA-P4 retained ≈9%. The reduction of activation barriers upon deprotonation thus explained both the enhancement in rate constants and the redistribution of branching ratios.

From an applied perspective, these findings suggest that water treatment processes based on hydroxyl radicals (*e.g.*, advanced oxidation processes) should be optimized at mildly alkaline conditions to ensure efficient removal of both compounds. While RDA will degrade readily across a broad pH window, achieving effective decomposition of NPA requires a shift toward alkaline environments. This distinction emphasizes the necessity of pH control in designing advanced oxidation protocols targeting fluorophore contaminants.

#### 3.1.5. The effects of temperature on the degradation of typical states

**Fig. 6:**
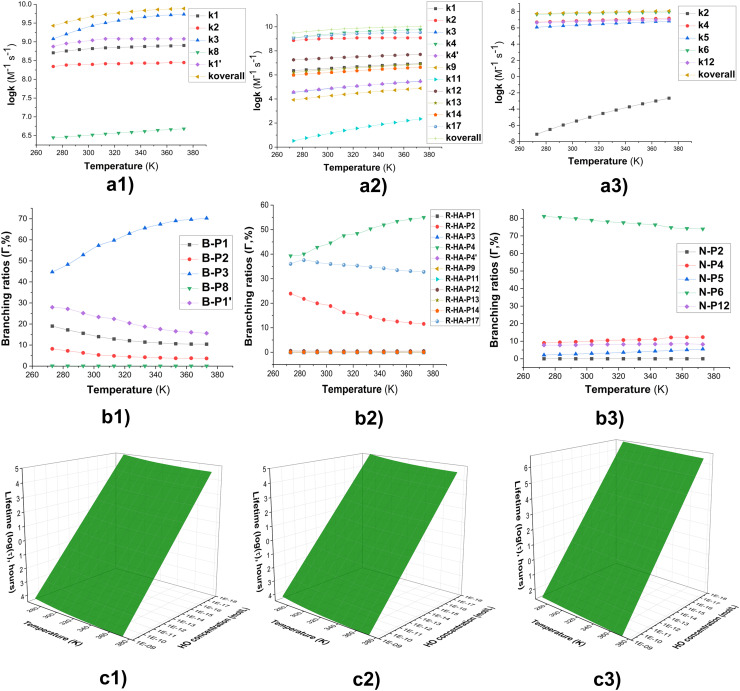
(a) The temperature influence on apparent rate constants (log *k*); (b) *Γ* values (%); (c) lifetime (log(*t*), h) at 273–373 K ((1): BOD; (2): RDA-HA; (3): NPA-HA).

To evaluate the effect of temperature on the degradation of the representative fluorophores, the rate constants for the reactions of the typical FPHs, including BOD, RDA-HA, and NPA with HO˙ were calculated in the range of 273–373 K (Fig. 6a). In all cases, the rate constants (log(*k*)) increased as the temperature rose, although the magnitude of this enhancement varied among the three compounds.

As illustrated in Fig. 6a1–6a3, the overall rate constant (log(*k*_overall_)) for the reaction between BOD and HO˙ radicals exhibited a moderate increase from 9.4 to 9.9, indicating that the RAF processes at the C1, C3, and C1′ sites remained predominant throughout the investigated temperature range. For the RDA-HA compound, log(*k*_overall_) increased by approximately 0.5 unit, primarily due to the synergistic contributions of the FHT(N17–H) and the RAF at the C2 and C4 positions. In contrast, the reaction of NPA with HO˙ radicals displayed only a slight variation in log(*k*_overall_), ranging from 7.8 to 8.1 within the 273–373 K.

As the temperature increased, the branching ratios of the principal intermediates exhibited systematic changes (Fig. 6b). In the case of BOD, the predominant intermediate (B-P3) increased markedly by 25.6%, whereas B-P1′ and B-P1 showed moderate declines from 28.0% to 15.6% and from 19.0% to 10.4%, respectively. For the RDA-HA state, the RAF product at the C4 position (R-HA-P4) rose from 39.3% to 55.0%, while the FHT (R-HA-P17/18) and RAF (R-HA-P2) products decreased slightly by 3.2 and 12.3 percentage units, respectively, indicating that the RAF(C4) pathway became increasingly favored at elevated temperatures. In contrast, NPA exhibited persistent selectivity toward the C6 pathway (N-P6), which remained the dominant route with branching ratios between 81.1% and 74.0% over the examined temperature range, while the C4 (N-P4) and C10 (N-P10) intermediates fluctuated within 9.0–12.3% and 7.8–8.2%, respectively.

The environmental lifetimes (*τ*) of the fluorophores under HO˙ exposure were estimated over the temperature range of 273–373 K (Fig. 6c). Considering environmentally relevant hydroxyl radical concentrations 10^−18^ to 10^−15^ M in natural water, 10^−10^ to 10^−9^ M in AOP-treated wastewater,^21,70^ the calculated lifetimes spanned from several hours to multiple years, depending on both radical concentration and temperature. For BOD, log(*τ*) decreased notably from 5.02 at 273 K to 1.55 at 373 K in natural waters. Similarly, RDA-HA lifetime also decreased, from 4.96 to 1.43. In contrast, NPA displayed relatively higher persistence, with log(*τ*) values of 6.69 at 273 K and 3.36 at 373 K, even under oxidative environments. Under AOP-treated wastewater conditions ([HO˙] = 10^−10^ to 10^−9^ M), the degradation of BOD and RDA-HA was predicted to occur within seconds, whereas NPA decomposition would still require several minutes.

### 3.2. The reaction of fluorophores with HO˙ in organic solvents

The degradation of FPHs by hydroxyl radicals was further investigated in organic solvents, namely methanol and pentyl ethanoate, to simulate conditions relevant to sensor applications in biological and lipid-like environments.^71–74^ The kinetic parameters are summarized in Table 4 (methanol) and Table 5 (pentyl ethanoate).

**Table 4:** Calculated Δ*G*^‡^ (kcal mol^−1^), *k*_Eck_, *k*_overall_ (M^−1^ s^−1^), *k* and *Γ* (%) of the reaction between HO˙ and FPHs reactions at 298.15 K in methanol

Mechanism		BOD			RDA			NPA		
		Δ*G*^‡^	*k*_app_	*Γ*	Δ*G*^‡^	*k*_app_	*Γ*	Δ*G*^‡^	*k*_app_	*Γ*
FHT	N2–H							17.1	4.40	0.0
	N17–H				5.4	1.80 × 10^9^	26.8			
RAF	C1	6.0	4.80 × 10^8^	9.4	8.8	5.30 × 10^6^	0.1			
	C1′	6.0	4.60 × 10^8^	9.0						
	C2	6.2	3.40 × 10^8^	6.7	5.4	1.10 × 10^9^	16.4			
	C3	3.9	3.80 × 10^9^	74.7	11.1	1.00 × 10^5^	0.0			
	C4				3.5	3.80 × 10^9^	56.5	8.2	1.50 × 10^7^	12.7
	C4′				10.9	1.50 × 10^5^	0.0			
	C5							9.1	3.10 × 10^6^	2.6
	C6							7.2	8.20 × 10^7^	69.4
	C8	8.2	6.70 × 10^6^	0.1						
	C9				11.6	2.40 × 10^4^	0.0			
	C10							8.1	1.80 × 10^7^	15.2
	C11				15.8	1.90 × 10^1^	0.0			
	C12				8.3	1.10 × 10^7^	0.2			
	C13				9.2	2.80 × 10^6^	0.0			
	C14				9.0	2.00 × 10^6^	0.0			
***k***_**overall**_			**5.09 × 10** ^**9**^			**6.72 × 10** ^**9**^			**1.18 × 10** ^**8**^	

**Table 5:** Calculated Δ*G*^‡^ (kcal mol^−1^), *k*_Eck_, *k*_overall_ (M^−1^ s^−1^), *k* and *Γ* (%) of the reaction between HO˙ and FPHs reactions at 298.15 K in pentyl ethanoate

Mechanism		BOD			RDA			NPA		
		Δ*G*^‡^	*k*_app_	*Γ*	Δ*G*^‡^	*k*_app_	*Γ*	Δ*G*^‡^	*k*_app_	*Γ*
FHT	N2–H							16.1	1.00 × 10^1^	0.0
	N17–H				10.8	5.50 × 10^6^	4.7			
RAF	C1	6.7	1.70 × 10^8^	12.3	10.5	3.70 × 10^5^	0.3			
	C1′	8.8	4.60 × 10^6^	0.3						
	C2	8.9	4.20 × 10^6^	0.3	7.7	3.40 × 10^7^	29.2			
	C3	5.3	1.20 × 10^9^	86.9	14.4	4.50 × 10^2^	0.0			
	C4				7.1	7.50 × 10^7^	64.4	10.0	7.60 × 10^5^	4.4
	C4′				13.1	3.90 × 10^3^	0.0			
	C5							9.9	9.30 × 10^5^	5.4
	C6							8.2	1.50 × 10^7^	86.5
	C8	9.0	1.70 × 10^6^	0.1						
	C9				13.6	8.50 × 10^2^	0.0			
	C10							9.6	6.60 × 10^5^	3.8
	C11				16.9	3.40	0.0			
	C12				9.9	9.00 × 10^5^	0.8			
	C13				10.2	5.30 × 10^5^	0.5			
	C14				10.7	1.10 × 10^5^	0.1			
**Total**			**1.38 × 10** ^**9**^			**1.16 × 10** ^**8**^			**1.74 × 10** ^**7**^	

As shown in Table 4, the overall rate constant of the BOD + HO˙ reaction in methanol reached 5.09 × 10^9^ M^−1^ s^−1^, with the C3 addition (B-P3) dominating at *Γ* = 74.7%, followed by C1 (9.4%) and C1′ (9.0%). In pentyl ethanoate, *k*_overall_ decreased to 1.38 × 10^9^ M^−1^ s^−1^, and C3 addition became even more selective (*Γ* = 86.9%), while C1 was the only notable secondary product (*Γ* = 12.3%). Thus, BOD degrades efficiently in both solvents, with methanol leading to faster kinetics and pentyl ethanoate being more restrictive in the possible products.

For RDA, methanol introduced multiple competing pathways. The overall rate constant was high (6.72 × 10^9^ M^−1^ s^−1^), with RAF at C4 (*Γ* = 56.5%, R-P4), RAF at C2 (*Γ* = 16.4%, R-P2), and FHT at N17–H (*Γ* = 26.8%, R-P17) all contributing significantly. In pentyl ethanoate, however, the overall rate dropped to 1.16 × 10^8^ M^−1^ s^−1^, nearly two orders of magnitude lower, while the dominant mechanism changed almost entirely to RAF. Specifically, C4 addition (*Γ* = 64.4%) and C2 addition (*Γ* = 29.2%) dominated, with FHT becoming negligible (*Γ* < 5%). This demonstrates that proton-transfer channels are strongly solvent-dependent and suppressed in nonpolar media.

For NPA, reactivity was consistently lower than BOD and RDA. In methanol, *k*_overall_ was 1.18 × 10^8^ M^−1^ s^−1^, dominated by C6 addition (*Γ* = 69.4%, N-P6), with secondary contributions from C10 (15.2%) and C4 (12.7%). In pentyl ethanoate, the overall rate decreased further to 1.74 × 10^7^ M^−1^ s^−1^, with selectivity narrowing almost exclusively on C6 addition (*Γ* = 86.5% *vs.* C5 5.4% and C10 3.8%) became minor. Thus, solvent effects amplify the persistence of NPA and limit its product diversity.

Overall, the solvent dielectric constant has a strong effect on fluorophore degradation kinetics. In methanol, both RDA and BOD undergo fast decomposition with multiple pathways, whereas in lipid-like pentyl ethanoate, only BOD maintains high reactivity, while RDA and NPA degrade more slowly. This finding has practical implications: fluorescent labels intended for biological or lipid environments may exhibit enhanced stability (particularly NPA), whereas BOD-based probes are prone to rapid oxidative degradation across both hydrophilic and lipophilic conditions.

### 3.3. Environmental risk assessment

#### 3.3.1. Ecological toxicity

**Fig. 7:**
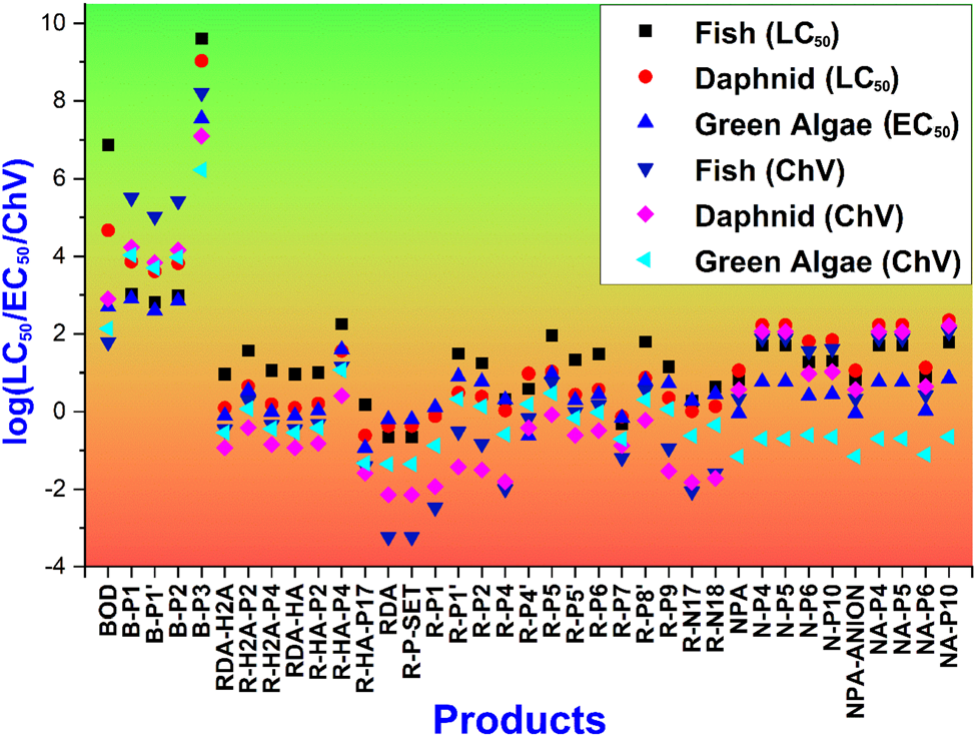
Acute and chronic toxicity (log(LC_50_/EC_50_/ChV), mg L^−1^) of FPHs and the main products.

The ecological toxicity of the FPHs and their hydroxyl radical-derived intermediates was evaluated against three representative aquatic species (fish, daphnids, and green algae), using acute (LC_50_, EC_50_) and chronic (ChV) endpoints. Fig. 7 presents the toxicity profiles as log(LC_50_/EC_50_/ChV, mg L^−1^), where values < 2 indicate toxic effects, and lower values correspond to higher toxicity.^75^

For BOD and its primary degradation products (B-P1, B-P1′, B-P2, B-P3), the log(LC_50_/EC_50_/ChV) values are predicted to be consistently above 2, indicating low toxicity across all organisms. This suggests that BOD, despite its high reactivity with HO˙ radicals, and its major intermediates, are unlikely to pose significant acute or chronic ecological risks.

By contrast, RDA and its degradation intermediates are predicted to display strong toxicity potential. The parent species RDA-H_2_A, RDA-HA, and neutral RDA all produced intermediates with log values < 2, notably R-H_2_A-P4, R-HA-P17, and several neutral RAF products (R-P2, R-P4, R-P5, R-P6, R-P9). These results may indicate toxicity against all three tested organisms, particularly green algae and daphnid. Furthermore, the SET product (R-P-SET) is predicted to fall in the toxic range, underscoring the ecological risks associated with incomplete oxidative degradation of RDA.

For NPA, the neutral form and several RAF intermediates, including N-P4, N-P6, and N-P10, are predicted to exhibit values < 2, suggesting toxicity primarily toward algae. The anionic form of NPA and its associated products (NA-P5, NA-P10) also entered the toxic range, further emphasizing the role of pH in determining ecological risk.

Overall, the results highlight substantial differences among the three fluorophores. BOD and its products are ecologically benign, while RDA degradation is predicted to generate highly toxic intermediates, and NPA might exhibit moderate but pH-dependent toxicity. Therefore, it is critical to recognize that although hydroxyl radical attack accelerates fluorophore degradation, the process may simultaneously yield toxic intermediates, especially in the case of RDA. These findings underline the importance of coupling degradation efficiency assessments with toxicity evaluations when predicting the environmental fate of fluorophores.

#### 3.3.2. Bioconcentration and biodegradability

The ecological risk posed by fluorophores depends not only on their reactivity and toxicity but also on their potential for bioaccumulation and biodegradation. Estimating bioconcentration factors (BCFs) provides a rational measure of organismal exposure to these chemicals and their degradation products.^76,77^ Typically, compounds with BCF values above 5000 are considered to have high bioaccumulation potential.^78^ The predicted BCF values of the studied fluorophores and their intermediates are summarized in Table S2, SI.

For BOD and its RAF-derived products (B-P1, B-P1′, B-P2, B-P3), BCF values were consistently low (≈3.16), far below the threshold for significant bioaccumulation. This result indicates that, despite their persistence in the aquatic environment, BOD and its breakdown products are unlikely to accumulate substantially in aquatic organisms. In contrast, RDA and its degradation intermediates are predicted to exhibit much higher BCF values. The parent neutral RDA and its SET-derived product (R-P-SET) reached values above 1400, while other products, such as R-P7, R-P1, and R-P17, fell in the range of 350–700. These values, although below the 5000 threshold, are several orders of magnitude greater than those of BOD and NPA, suggesting that RDA and its products may accumulate significantly in fish tissues and other organisms, thereby elevating ecological risk. For NPA and its products, both the neutral and anionic forms showed low BCF values (≈9.6), and all intermediates (N-P4, N-P5, N-P6, N-P12, NA-P4, NA-P5, NA-P6, NA-P10) remained below 5. These results suggest that NPA, similar to BOD, does not present substantial bioaccumulation potential.

The biodegradability of these fluorophores was evaluated using the BIOWIN 3, 4, and 5 models in EPI Suite,^64^ (Table S2, SI). The results indicate that all parent compounds and their major products are non-biodegradable, with degradation timelines extending from weeks to months. While some initial biodegradation may occur, the persistence of both the reactants and their intermediates is predicted to be high.

In summary, although BOD and NPA do not accumulate in living organisms, their poor biodegradability suggests that they may persist in aquatic systems. RDA and its intermediates are predicted to present the most severe concern, combining poor biodegradability with relatively high BCF values, thus posing the greatest long-term ecological hazard among the three fluorophore systems.

## 4. Conclusion

A comprehensive computational investigation was conducted to assess the hydroxyl radical-driven degradation pathways of three common fluorophores: RDA, NPA, and BOD. These fluorophores have markedly different reactivities and thus varying environmental lifetimes, ranging from a few hours for neutral RDA to several months or years for neutral NPA. The radical degradation predominantly proceeds *via* the RAF and FHT mechanisms, while the SET pathway also contributes to neutral RDA. The protonation states determine the degradation pathways of RDA and NPA but do not affect the degradation of BOD. It was established that RAD, NPA and their intermediates are predicted to exhibit high ecotoxicity (log LC_50_/EC_50_/ChV < 2), poor biodegradability, and strong bioaccumulation potential, whereas the degradation products of BOD are expected to be less toxic and more environmentally benign despite their persistence. Overall, the study yielded a hazard ranking of RDA ≈ NPA > BOD, underscoring that although hydroxyl radical oxidation efficiently progresses fluorophore degradation, it may simultaneously generate harmful by products.

## Conflicts of interest

There are no conflicts to declare.

## Supplementary Material

RA-016-D6RA00906A-s001

## Data Availability

The data supporting this article have been included as part of the supplementary information (SI). Supplementary information is available. See DOI: https://doi.org/10.1039/d6ra00906a.
