# Supplementary material for: The environmental degradation of naphthalimide, rhodamine and BODIPY fluorophores by hydroxyl radicals: a theoretical insight
Source: RSC Adv. 2026 Mar 5;16(14):12558–69. doi: 10.1039/d6ra00906a (PMC12961683; doi:10.1039/d6ra00906a)
Supplement: RA-016-D6RA00906A-s001 [file RA-016-D6RA00906A-s001.pdf]

Supplementary Information (SI) for RSC Advances.
This journal is © The Royal Society of Chemistry 2026

Supporting Information (SI)

The environmental degradation of naphthalimide, rhodamine and bodipy fluorophores by hydroxyl radicals: A theoretical insight

Nguyen Linh Nam,[1*] Mai Van Bay[2*], Nguyen Thi Hoa,[1] Nguyen Quang Trung,[3] Nguyen Minh Thong[2], Adam Mechler[4], Pham Cam Nam[5] Nguyen Khoa Hien,[6] Duong Tuan Quang[7] and Quan V. Vo[1**]

[1]The University of Danang - University of Technology and Education, Danang 550000, Vietnam.
[2]The University of Danang - University of Sciences and Education, Danang 550000, Vietnam.
[3]Quality assurance and Testing center 2, Da Nang 550000, Vietnam.
[4]Department of Biochemistry and Chemistry, La Trobe University, Victoria 3086, Australia.
[5]The University of Danang - University of Technology and Sciences, Danang 550000, Vietnam.
[6]Mientrung Institute for Scientific Research, Vietnam National Museum of Nature, Vietnam Academy of Science and Technology, Hue 530000, Vietnam
[7]Department of Chemistry, Hue University, Hue 530000, Vietnam
**: First authors have equal contributions.*
***Corresponding authors: vvquan@ute.udn.vn;*

Table of Contents

Table S1. The method to calculate rate constant following the conventional transition state theory S2

Table S2. Bioconcentration (BCF) and biodegradability of FPHs and the main degradation products.................S4

Table S3: The Cartesian coordinates, energies of TS of the reaction between FPHs with HO• in the studied media (M: methanol; P: pentyl ethanoate; H: water)...S6

References S92

Table S1. The method to calculate rate constant following the conventional transition state theory

The rate constant (k) was calculated by using the conventional transition state theory (TST) (at 298.15 K, 1M standard state) according to the equation (1):[1-5]

$$k = \sigma\kappa \frac{k_B T}{h} e^{-(\Delta G^{\neq})/RT} \quad (1)$$

Where: σ is the reaction symmetry number ,[6-7]

κ contains the tunneling corrections calculated using the Eckart barrier,[8]

k_B is the Boltzmann constant,

h is the Planck constant,

$\Delta G^{\neq}$ is the Gibbs free energy of activation.

The Marcus Theory was used to estimate the reaction barriers of SET reactions.[9-12] The free energy of reaction $\Delta G^{\neq}$ for the SET pathway was computed following the equations (2,3).

$$\Delta G^{\neq}_{SET} = \frac{\lambda}{4}\left(1 + \frac{\Delta G^{0}_{SET}}{\lambda}\right)^2 \quad (2)$$

$$\lambda \approx \Delta E_{SET} - \Delta G^{0}_{SET} \quad (3)$$

where ΔG_{SET} is the Gibbs energy of reaction, ΔE_{SET} is the non-adiabatic energy difference between reactants and vertical products for SET.[13-14]

For rate constants that were close to the diffusion limit a correction was applied to yield realistic results[15]. The apparent rate constants (k_{app}) were calculated following the Collins–Kimball theory in the solvents at 298.15K;[16] the steady-state Smoluchowski rate constant (k_D) for an irreversible bimolecular diffusion–controlled reaction was calculated following the literature as corroding to equations (4,5).[15,17]

$$k_{app} = \frac{k_{TST} k_D}{k_{TST} + k_D} \quad (4)$$

$$k_D = 4\pi R_{AB} D_{AB} N_A \quad (5)$$

where R_{AB} is the reaction distance, N_A is the Avogadro constant, and $D_{AB} = D_A + D_B$ (D_{AB} is the mutual diffusion coefficient of the reactants A and B),[16,18] where D_A or D_B is estimated using the

Stokes–Einstein formulation (6).[19-20]

$$D_{A\ or\ B} = \frac{k_{\mathrm{B}}T}{6\pi\eta a_{A\ or\ B}} \quad (6)$$

η is the viscosity of the solvents at 298.15 K (i.e. $\eta(H_2O) = 8.91\times10^{-4}$ Pa s, η(pentyl ethanoate) = 8.62×10^{-4} Pa s, η(methanol) = 5.43×10^{-4} Pa s) and *a* is the radius of the solute.

All transition states were characterized by the existence of only one single imaginary frequency. Intrinsic coordinate calculations (IRCs) were performed to ensure that each transition state is connected correctly with the pre-complex and post-complex.

Table S2. Bioconcentration (BCF) and biodegradability of FPHs and the main degradation products.

Products	BCF	Biodegradability			
		BIOWIN3	BIOWIN4	BIOWIN5	Biodegradable
**BOD**	3.162	2.7727 (weeks)	3.5693 (days-weeks)	0.2192	NO
**B-P1**	3.162	2.8929 (weeks)	3.6728 (days-weeks)	0.3393	NO
**B-P1'**	3.162	2.5208 (weeks-months)	3.3899 (days-weeks)	0.2406	NO
**B-P2**	3.162	2.8929 (weeks)	3.6728 (days-weeks)	0.3404	NO
**B-P3**	3.162	2.8929 (weeks)	3.6728 (days-weeks)	0.3404	NO
**RDA-H2A**	59.110	2.5997 (weeks-months	3.4701 (days-weeks)	–0.0078	NO
**R-H2A-P2**	15.400	2.7243 (weeks-months)	3.5765 (days-weeks)	0.0995	NO
**R-H2A-P4**	49.900	2.5730 (weeks-months)	3.4568 (days-weeks)	–0.0631	NO
**RDA-HA**	59.110	2.5997 (weeks-months)	2.5997 (weeks-months)	–0.0078	NO
**R-HA-P2**	47.360	2.7199 (weeks-months)	3.5736 (days-weeks)	0.1195	NO
**R-HA-P4**	3.162	2.7199 (weeks-months)	3.5736 (days-weeks)	0.1184	NO
**R-HA-P17**	353.600	2.5997 (weeks-months)	3.4701 (days-weeks)	–0.0078	NO
**RDA**	1419.000	2.3953 (weeks-months)	3.4083 (days-weeks)	–0.0179	NO
**R-P-SET**	1419.000	2.3953 (weeks-months)	3.4083 (days-weeks)	–0.0179	NO
**R-P1**	369.600	2.5200 (weeks-months)	3.5146 (days-weeks)	0.0895	NO
**R-P1'**	10.330	2.0686 (months)	3.1603 (weeks)	–0.0136	NO
**R-P2**	18.260	2.5200 (weeks-months)	3.5146 (days-weeks)	0.0895	NO
**R-P4**	156.000	2.5155 (weeks-months)	3.5117 (days-weeks)	0.1084	NO
**R-P4'**	23.750	2.1434 (months)	3.2288 (weeks)	0.0097	NO
**R-P5**	6.226	2.6559 (weeks-months)	3.5163 (days-weeks)	0.0631	NO
**R-P5'**	26.550	2.3523 (weeks-months)	3.2936 (days-weeks)	–0.0003	NO
**R-P6**	19.180	2.2090 (months)	3.1648 (weeks)	–0.0888	NO
**R-P7**	684.100	2.4164 (weeks-months)	3.4249 (days-weeks)	–0.0094	NO
**R-P8'**	9.101	2.3523 (weeks-months)	3.2936 (days-weeks)	–0.0003	NO
**R-P9**	22.620	2.1434 (months)	3.2288 (weeks)	0.0086	NO
**R-N17**	172.800	2.4159 (weeks-months)	3.3066 (days-weeks)	–0.2576	NO
**R-N18**	70.110	2.3953 (weeks-months)	3.4083 (days-weeks)	–0.0179	NO

**NPA**	9.588	2.7634 (weeks)	3.5632 (days-weeks)	0.1748	NO
**N-P4**	3.162	2.8836 (weeks)	3.6667 (days-weeks)	0.2801	NO
**N-P5**	3.162	2.8836 (weeks)	3.6667 (days-weeks)	0.2801	NO
**N-P6**	3.162	2.8836 (weeks)	3.6667 (days-weeks)	0.2889	NO
**N-P12**	3.162	2.5115 (weeks-months)	3.3838 (days-weeks)	0.1891	NO
**NPA - ANION**	9.588	2.7634 (weeks)	3.5632 (days-weeks)	0.1748	NO
**NA-P4**	3.162	2.8836 (weeks)	3.6667 (days-weeks)	0.2801	NO
**NA-P5**	3.162	2.8836 (weeks)	3.6667 (days-weeks)	0.2801	NO
**NA-P6**	4.623	2.7844 (weeks)	3.5798 (days-weeks)	0.1832	NO
**NA-P10**	3.162	2.5115 (weeks-months)	3.3838 (days-weeks)	0.1891	NO

Table S3: The Cartesian coordinates, energies of TS of the reaction between FPHs with HO$^{\bullet}$ in the studied media (M: methanol; P: pentyl ethanoate; H: water)

Cartesian coordinates / Energies

BOD-C1'-OH-TS-H

Atom	X	Y	Z		
C	0.66169	-0.186783	-0.051727	Electronic Energy (EE)	-757.065
C	-0.110555	-1.270245	-0.384039	Zero-point Energy Correction	0.164047
C	0.730839	-2.411887	-0.31842	Thermal Correction to Energy	0.176206
B	3.196636	-2.960632	0.281627	Thermal Correction to Enthalpy	0.17715
C	5.700255	-2.426951	-0.113173	Thermal Correction to Free Energy	0.125256
C	6.543722	-1.288325	-0.186028		
C	5.732087	-0.18416	-0.074036		
C	3.208351	0.019295	0.180795		
C	4.394715	-0.657161	0.058756		
N	4.4337	-2.055767	0.023556		
C	1.9764	-0.676087	0.248647		
N	1.963483	-2.058038	0.025061		
F	3.191459	-4.059344	-0.562698		
F	3.199611	-3.373531	1.622823		
H	0.375628	0.85325	0.002634		
H	-1.155588	-1.283816	-0.650044		
H	0.480106	-3.446362	-0.50941		
H	5.974002	-3.472586	-0.154334		
H	7.61453	-1.315057	-0.311541		
H	6.014223	0.858166	-0.090439		
H	3.203105	1.102292	0.218824		
O	1.816589	-0.625941	2.342541		
H	2.396194	-1.392901	2.467389		

BOD-C1'-OH-TS-M

Atom	X	Y	Z		
C	0.654759	-0.170912	-0.226671	Electronic Energy (EE)	-757.071
C	-0.15002	-1.280103	-0.342793	Zero-point Energy Correction	0.163755
C	0.69124	-2.413777	-0.202641	Thermal Correction to Energy	0.176092
B	3.176552	-2.933835	0.301687	Thermal Correction to Enthalpy	0.177036
C	5.668561	-2.390354	-0.166034	Thermal Correction to Free Energy	0.124322
C	6.510871	-1.248914	-0.224927		
C	5.721059	-0.157886	0.032054		
C	3.164487	0.046452	0.133935		
C	4.392915	-0.641465	0.279025		

N	4.419858	-2.029653	0.103451
C	1.985398	-0.636822	-0.023428
N	1.95011	-2.035171	-0.020747
F	3.22768	-4.032581	-0.54323
F	3.108154	-3.349015	1.638939
H	0.371354	0.870122	-0.283059
H	-1.214976	-1.312486	-0.512469
H	0.421444	-3.461277	-0.227082
H	5.930085	-3.429895	-0.311646
H	7.568902	-1.267806	-0.434962
H	6.003606	0.884164	0.070709
H	3.165925	1.130489	0.141344
O	4.460718	-0.503053	2.371193
H	3.865508	-1.253763	2.52193

BOD-C1'-OH-TS-P

Atom	X	Y	Z		
				Electronic Energy (EE)	-757.063
C	0.666362	-0.186779	-0.030256	Zero-point Energy Correction	0.164112
C	-0.089042	-1.263409	-0.412082	Thermal Correction to Energy	0.176297
C	0.753615	-2.405559	-0.336597	Thermal Correction to Enthalpy	0.177241
B	3.205853	-2.958293	0.318867	Thermal Correction to Free Energy	0.125247
C	5.704127	-2.430976	-0.133809		
C	6.540668	-1.295201	-0.224558		
C	5.725812	-0.188542	-0.096113		
C	3.204469	0.012583	0.214606		
C	4.397733	-0.663559	0.064529		
N	4.437682	-2.056932	0.030042		
C	1.97572	-0.676487	0.310428		
N	1.968129	-2.058426	0.057585		
F	3.189545	-4.070215	-0.503966		
F	3.218734	-3.333559	1.668864		
H	0.370652	0.849569	0.040032		
H	-1.12466	-1.273446	-0.713022		
H	0.516117	-3.437118	-0.558224		
H	5.971498	-3.4777	-0.179515		
H	7.608979	-1.315704	-0.371724		
H	6.010058	0.853188	-0.118355		
H	3.200877	1.095353	0.256593		
O	1.805592	-0.602535	2.303692		
H	2.282662	-1.428825	2.479746		

BOD-C1-OH-TS-H

Atom	X	Y	Z		
				Electronic Energy (EE)	-757.063
C	0.56907	-0.263944	-0.09421	Zero-point Energy Correction	0.162907

C	-0.229015	-1.414241	-0.230494	Thermal Correction to Energy	0.175579
C	0.627855	-2.502493	-0.193242	Thermal Correction to Enthalpy	0.176523
B	3.166726	-2.992232	0.019688	Thermal Correction to Free Energy	0.122224
C	5.676288	-2.385962	0.078787		
C	6.500773	-1.233441	0.117138		
C	5.660751	-0.143671	0.105936		
C	3.116963	0.009144	0.026306		
C	4.32799	-0.642574	0.059914		
N	4.393852	-2.036815	0.043597		
C	1.919554	-0.716487	-0.037138		
N	1.915322	-2.087665	-0.075517		
F	3.23884	-3.845137	-1.084186		
F	3.133808	-3.757981	1.189382		
H	0.27144	0.766878	-0.209239		
H	-1.302435	-1.454797	-0.323597		
H	0.396024	-3.557014	-0.245779		
H	5.971305	-3.426602	0.076416		
H	7.57882	-1.240476	0.147868		
H	5.922635	0.903842	0.125634		
H	3.084303	1.092375	0.047105		
O	0.581471	-0.008789	2.12181		
H	1.351111	0.5818	2.166281		

BOD-C1-OH-TS-M

Atom	X	Y	Z		
				Electronic Energy (EE)	-757.069
C	0.569879	-0.26032	-0.097003	Zero-point Energy Correction	0.163002
C	-0.228358	-1.41007	-0.232478	Thermal Correction to Energy	0.175599
C	0.628254	-2.498905	-0.192046	Thermal Correction to Enthalpy	0.176544
B	3.166375	-2.989487	0.022127	Thermal Correction to Free Energy	0.122715
C	5.676639	-2.386076	0.083223		
C	6.502236	-1.234201	0.116491		
C	5.663102	-0.143587	0.099273		
C	3.1186	0.010872	0.019157		
C	4.329991	-0.641723	0.055117		
N	4.394704	-2.035454	0.045761		
C	1.920791	-0.713504	-0.039936		
N	1.915251	-2.084782	-0.074314		
F	3.238756	-3.842053	-1.082184		
F	3.132533	-3.753411	1.192782		
H	0.271309	0.770671	-0.211846		
H	-1.302279	-1.450171	-0.324246		
H	0.395533	-3.55372	-0.241566		
H	5.970423	-3.427403	0.086006		
H	7.580658	-1.241746	0.148177		
H	5.926864	0.903936	0.114192		

H	3.087642	1.09458	0.033768
O	0.571232	-0.026944	2.115574
H	1.343317	0.557216	2.196429

BOD-C1-OH-TS-P

Atom	X	Y	Z		
C	0.616895	-0.198733	-0.09974	Electronic Energy (EE)	-757.064
C	-0.196983	-1.348372	-0.159713	Zero-point Energy Correction	0.163362
C	0.650256	-2.445279	-0.106943	Thermal Correction to Energy	0.176016
B	3.187934	-2.959037	0.058113	Thermal Correction to Enthalpy	0.17696
C	5.704513	-2.378447	0.071793	Thermal Correction to Free Energy	0.122712
C	6.54164	-1.236371	0.069743		
C	5.712274	-0.136774	0.034492		
C	3.166644	0.041125	-0.031947		
C	4.37571	-0.621983	0.01309		
N	4.425916	-2.013709	0.037122		
C	1.964829	-0.669105	-0.071303		
N	1.943443	-2.040835	-0.050635		
F	3.237904	-3.840456	-1.019727		
F	3.158431	-3.678509	1.252946		
H	0.325939	0.820449	-0.29734		
H	-1.273322	-1.378727	-0.217561		
H	0.408227	-3.498701	-0.106026		
H	5.98268	-3.423225	0.098649		
H	7.619901	-1.252332	0.092128		
H	5.989068	0.907081	0.022545		
H	3.144385	1.124512	-0.03505		
O	0.616268	0.388258	1.96458		
H	0.5709	-0.517113	2.313244		

BOD-C2-OH-TS-H

Atom	X	Y	Z		
C	0.704982	-0.097015	-0.094321	Electronic Energy (EE)	-757.063
C	-0.165803	-1.206463	-0.030963	Zero-point Energy Correction	0.163627
C	0.678787	-2.364556	-0.055444	Thermal Correction to Energy	0.176055
B	3.176212	-2.951949	0.055541	Thermal Correction to Enthalpy	0.176999
C	5.701876	-2.440942	0.062403	Thermal Correction to Free Energy	0.123686
C	6.568934	-1.322951	0.04694		
C	5.770119	-0.200451	0.010627		
C	3.240117	0.060527	-0.037344		
C	4.421207	-0.646156	0.002807		
N	4.432491	-2.042426	0.036704		
C	2.00258	-0.593609	-0.05124		
N	1.947406	-2.00049	-0.03298		

F	3.187613	-3.82471	-1.032501
F	3.125212	-3.687922	1.240643
H	0.434584	0.946988	-0.134455
H	-1.218026	-1.212141	-0.265355
H	0.384623	-3.405298	-0.050739
H	5.95539	-3.492036	0.091839
H	7.646315	-1.369976	0.061613
H	6.073092	0.835905	-0.010775
H	3.265021	1.143857	-0.061271
O	-0.502633	-1.442477	1.975954
H	-1.061188	-2.231183	1.907909

BOD-C2-OH-TS-M

Atom	X	Y	Z		
C	0.621297	-0.198361	-0.138518	Electronic Energy (EE)	-757.07
C	-0.170516	-1.32606	-0.112046	Zero-point Energy Correction	0.163594
C	0.702698	-2.436114	-0.023799	Thermal Correction to Energy	0.176024
B	3.229527	-2.924856	0.111518	Thermal Correction to Enthalpy	0.176968
C	5.725871	-2.321397	0.088156	Thermal Correction to Free Energy	0.123596
C	6.560387	-1.15701	0.108728		
C	5.68659	-0.057074	-0.013497		
C	3.149549	0.081693	-0.069288		
C	4.390581	-0.562574	-0.007435		
N	4.454517	-1.966953	0.052938		
C	1.972143	-0.633211	-0.067428		
N	1.968699	-2.027592	0.002762		
F	3.276152	-3.829633	-0.949483		
F	3.226761	-3.623553	1.320031		
H	0.3117	0.834706	-0.202786		
H	-1.247327	-1.381405	-0.149844		
H	0.455504	-3.488506	0.021456		
H	6.026342	-3.359749	0.131924		
H	7.622866	-1.159339	-0.075568		
H	5.95196	0.987782	-0.071711		
H	3.118479	1.164039	-0.124517		
O	6.80772	-1.356367	2.147309		
H	7.399141	-2.124321	2.128154		

BOD-C2-OH-TS-P

Atom	X	Y	Z		
C	0.69388	-0.107059	-0.102771	Electronic Energy (EE)	-757.061
C	-0.170164	-1.214665	-0.019364	Zero-point Energy Correction	0.163523
C	0.681441	-2.369568	-0.039205	Thermal Correction to Energy	0.176164
B	3.180822	-2.95732	0.027479	Thermal Correction to Enthalpy	0.177109
				Thermal Correction to Free Energy	0.122739

C	5.705519	-2.441885	0.079452
C	6.568975	-1.325142	0.067833
C	5.765916	-0.202056	0.017145
C	3.231402	0.053053	-0.049077
C	4.421673	-0.650983	-0.001579
N	4.434843	-2.042455	0.038705
C	1.999957	-0.598767	-0.059108
N	1.946934	-2.003429	-0.026279
F	3.189875	-3.778255	-1.098757
F	3.133999	-3.734918	1.179823
H	0.416473	0.934941	-0.146382
H	-1.221513	-1.223695	-0.25809
H	0.396537	-3.413074	-0.024841
H	5.954479	-3.49359	0.115652
H	7.646401	-1.366814	0.095429
H	6.071033	0.833757	-0.004011
H	3.2547	1.136486	-0.077839
O	-0.505759	-1.381625	1.963441
H	-1.028511	-2.198412	1.957936

BOD-C3-OH-TS-H

Atom	X	Y	Z		
C	0.649486	-0.146304	-0.117597	Electronic Energy (EE)	-757.067
C	-0.188752	-1.244077	-0.157211	Zero-point Energy Correction	0.16317
C	0.636457	-2.395481	-0.138981	Thermal Correction to Energy	0.175552
B	3.14862	-2.956827	0.034379	Thermal Correction to Enthalpy	0.176497
C	5.678461	-2.444339	0.060725	Thermal Correction to Free Energy	0.123473
C	6.53834	-1.320921	0.084402		
C	5.732914	-0.202623	0.069633		
C	3.19701	0.038225	-0.013684		
C	4.386557	-0.656439	0.033357		
N	4.406145	-2.051781	0.030024		
C	1.974415	-0.639501	-0.084713		
N	1.931711	-2.018205	-0.124757		
F	3.208398	-3.890961	-0.996887		
F	3.045231	-3.639956	1.263418		
H	0.379957	0.899094	-0.105467		
H	-1.267162	-1.25519	-0.184842		
H	0.359954	-3.431425	-0.270964		
H	5.937803	-3.494319	0.06688		
H	7.615796	-1.361745	0.108608		
H	6.029833	0.835662	0.077028		
H	3.205856	1.121814	-0.002727		
O	0.338463	-2.772519	2.123965		
H	1.226679	-3.170799	2.087029		

BOD-C3-OH-TS-M

Atom	X	Y	Z		
				Electronic Energy (EE)	-757.073
C	0.646557	-0.201759	-0.065677	Zero-point Energy Correction	0.163118
C	-0.151781	-1.325445	-0.053679	Thermal Correction to Energy	0.175557
C	0.715735	-2.442679	-0.022366	Thermal Correction to Enthalpy	0.176501
B	3.248279	-2.938506	0.03635	Thermal Correction to Free Energy	0.123215
C	5.761864	-2.359266	-0.00178		
C	6.580239	-1.202092	-0.012748		
C	5.734533	-0.110694	-0.043268		
C	3.182889	0.055867	-0.059304		
C	4.411719	-0.613188	-0.059619		
N	4.465742	-1.99161	-0.057422		
C	1.99612	-0.646645	-0.043682		
N	1.985647	-2.041197	-0.015961		
F	3.241074	-3.857775	-1.011282		
F	3.299179	-3.636789	1.258024		
H	0.341938	0.834416	-0.091208		
H	-1.229559	-1.37322	-0.065713		
H	0.463398	-3.494558	-0.002923		
H	6.051373	-3.396703	-0.090374		
H	7.659087	-1.205842	0.014124		
H	5.997184	0.93693	-0.045358		
H	3.166119	1.139657	-0.075752		
O	5.951012	-2.655173	2.279407		
H	5.089292	-3.107137	2.231219		

BOD-C3-OH-TS-P

Atom	X	Y	Z		
				Electronic Energy (EE)	-757.068
C	0.643272	-0.145313	-0.122566	Zero-point Energy Correction	0.164062
C	-0.190552	-1.237317	-0.137846	Thermal Correction to Energy	0.176252
C	0.636823	-2.395314	-0.090882	Thermal Correction to Enthalpy	0.177197
B	3.152894	-2.958058	0.031696	Thermal Correction to Free Energy	0.12464
C	5.682012	-2.444475	0.074901		
C	6.541299	-1.323269	0.09159		
C	5.734758	-0.20327	0.063466		
C	3.194451	0.037448	-0.025482		
C	4.390414	-0.657371	0.028255		
N	4.409256	-2.048619	0.037782		
C	1.976273	-0.638506	-0.082265		
N	1.931631	-2.015197	-0.094152		
F	3.203622	-3.859116	-1.025575		
F	3.061854	-3.665604	1.243233		
H	0.371814	0.899784	-0.125763		

H	-1.269053	-1.250519	-0.150723
H	0.363061	-3.427649	-0.249533
H	5.935229	-3.495652	0.089274
H	7.618806	-1.361728	0.120684
H	6.035736	0.83392	0.063658
H	3.203345	1.121139	-0.030686
O	0.326166	-2.79002	2.042553
H	1.219063	-3.173911	2.089998

BOD-C5-OH-TS-H

Atom	X	Y	Z		
C	0.646693	-0.201915	-0.067054	Electronic Energy (EE)	-757.067
C	-0.150773	-1.325923	-0.06124	Zero-point Energy Correction	0.163059
C	0.717265	-2.442869	-0.028442	Thermal Correction to Energy	0.175506
B	3.249603	-2.936846	0.050396	Thermal Correction to Enthalpy	0.176451
C	5.763018	-2.358	-0.009807	Thermal Correction to Free Energy	0.123208
C	6.580026	-1.200536	-0.014817		
C	5.73309	-0.109085	-0.039095		
C	3.182216	0.057111	-0.053371		
C	4.411536	-0.612262	-0.057669		
N	4.465913	-1.991022	-0.065718		
C	1.996636	-0.645779	-0.040671		
N	1.98697	-2.04106	-0.016179		
F	3.239339	-3.879797	-0.974827		
F	3.30524	-3.609038	1.288013		
H	0.342429	0.83396	-0.092366		
H	-1.228024	-1.374735	-0.078628		
H	0.465223	-3.494524	-0.011705		
H	6.052924	-3.394194	-0.106714		
H	7.65857	-1.20347	0.009598		
H	5.994247	0.938505	-0.03654		
H	3.165364	1.140581	-0.0655		
O	5.953354	-2.68202	2.272967		
H	5.076782	-3.100491	2.200376		

BOD-C6-OH-TS-H

Atom	X	Y	Z		
C	0.621147	-0.198472	-0.140903	Electronic Energy (EE)	-757.063
C	-0.1705	-1.326281	-0.115094	Zero-point Energy Correction	0.163656
C	0.702591	-2.436113	-0.025484	Thermal Correction to Energy	0.176081
B	3.229057	-2.925146	0.114259	Thermal Correction to Enthalpy	0.177025
C	5.72496	-2.320535	0.092117	Thermal Correction to Free Energy	0.12371
C	6.559668	-1.155735	0.116402		
C	5.684725	-0.055535	-0.008794		

C	3.148306	0.082725	-0.068716
C	4.389671	-0.56113	-0.005076
N	4.453736	-1.96634	0.054701
C	1.971895	-0.63281	-0.068071
N	1.968807	-2.027705	0.002563
F	3.276904	-3.833325	-0.94332
F	3.226429	-3.621539	1.324279
H	0.312365	0.834386	-0.205907
H	-1.246837	-1.382058	-0.154284
H	0.455348	-3.48816	0.019608
H	6.026055	-3.358399	0.136178
H	7.62043	-1.159058	-0.075557
H	5.949207	0.989102	-0.067871
H	3.116679	1.164677	-0.124253
O	6.825478	-1.352542	2.144034
H	7.394523	-2.135861	2.106246

BOD-C7-OH-TS-H

Atom	X	Y	Z		
C	0.73891	-0.13113	-0.014158	Electronic Energy (EE)	-757.063
C	-0.103315	-1.219114	-0.025831	Zero-point Energy Correction	0.163023
C	0.719409	-2.373569	-0.018206	Thermal Correction to Energy	0.175668
B	3.227643	-2.985248	0.03645	Thermal Correction to Enthalpy	0.176612
C	5.773991	-2.503099	-0.091749	Thermal Correction to Free Energy	0.12243
C	6.634139	-1.417215	-0.107049		
C	5.834451	-0.263946	-0.007239		
C	3.284419	0.015843	0.010415		
C	4.481497	-0.713212	0.003384		
N	4.483936	-2.084595	-0.02343		
C	2.071372	-0.632976	0.000025		
N	2.00296	-2.027253	-0.004704		
F	3.197996	-3.856918	-1.05445		
F	3.210925	-3.73078	1.219649		
H	0.478833	0.917006	-0.016478		
H	-1.181741	-1.223855	-0.039341		
H	0.42238	-3.413631	-0.021133		
H	6.004969	-3.5585	-0.127241		
H	7.710133	-1.460793	-0.160764		
H	6.138279	0.765196	-0.120853		
H	3.319082	1.099127	0.021486		
O	5.748039	0.002529	2.202697		
H	4.961913	0.572041	2.228922		

BOD-C8-OH-TS-H

Atom	X	Y	Z	Electronic Energy (EE)	-757.062
C	0.619527	-0.199821	-0.20261	Zero-point Energy Correction	0.16459
C	-0.186079	-1.324599	-0.318435	Thermal Correction to Energy	0.176563
C	0.658141	-2.442319	-0.209125	Thermal Correction to Enthalpy	0.177507
B	3.154146	-2.948342	0.219736	Thermal Correction to Free Energy	0.126021
C	5.678217	-2.403115	-0.050257		
C	6.521457	-1.262945	-0.103811		
C	5.709444	-0.161462	0.004911		
C	3.132465	0.045459	0.119011		
C	4.369823	-0.643793	0.129782		
N	4.401977	-2.027313	0.078627		
C	1.941444	-0.655909	-0.034278		
N	1.925956	-2.046912	-0.043436		
F	3.220177	-3.989455	-0.702271		
F	3.102425	-3.473249	1.517315		
H	0.324286	0.83829	-0.241992		
H	-1.253485	-1.358116	-0.469841		
H	0.404784	-3.492706	-0.245469		
H	5.948735	-3.448413	-0.106539		
H	7.594063	-1.288631	-0.212571		
H	5.984018	0.882613	0.007702		
H	3.13423	1.125575	0.05815		
O	3.805972	0.061947	2.172321		
H	3.484938	-0.819064	2.424778		

BOD-C8-OH-TS-M

Atom	X	Y	Z	Electronic Energy (EE)	-757.069
C	0.616627	-0.20028	-0.203931	Zero-point Energy Correction	0.164391
C	-0.189254	-1.325169	-0.311969	Thermal Correction to Energy	0.176423
C	0.655722	-2.442723	-0.201216	Thermal Correction to Enthalpy	0.177367
B	3.153705	-2.949649	0.208044	Thermal Correction to Free Energy	0.125623
C	5.678055	-2.403235	-0.03968		
C	6.521582	-1.26366	-0.093517		
C	5.708819	-0.161462	0.005492		
C	3.130921	0.045377	0.110042		
C	4.368551	-0.643233	0.124618		
N	4.40126	-2.02673	0.08079		
C	1.939635	-0.656003	-0.038846		
N	1.923632	-2.046737	-0.041149		
F	3.220107	-3.975674	-0.731493		
F	3.102409	-3.49554	1.496786		
H	0.320553	0.837913	-0.24553		
H	-1.257701	-1.35918	-0.45864		
H	0.402107	-3.493569	-0.232426		
H	5.94858	-3.449206	-0.08942		

H	7.595355	-1.289464	-0.194389
H	5.984226	0.882778	0.00762
H	3.132451	1.125843	0.048602
O	3.815039	0.068946	2.161848
H	3.504281	-0.811624	2.430063

BOD-C8-OH-TS-P

Atom	X	Y	Z		
C	0.622231	-0.19893	-0.209331	Electronic Energy (EE)	-757.063
C	-0.17343	-1.325458	-0.393064	Zero-point Energy Correction	0.164482
C	0.669626	-2.441173	-0.283744	Thermal Correction to Energy	0.17657
B	3.151065	-2.906392	0.31506	Thermal Correction to Enthalpy	0.177515
C	5.669195	-2.396608	-0.130917	Thermal Correction to Free Energy	0.12551
C	6.513506	-1.257724	-0.17566		
C	5.707363	-0.155827	-0.010251		
C	3.134124	0.040428	0.185215		
C	4.372051	-0.63667	0.121021		
N	4.40114	-2.019495	0.034294		
C	1.936634	-0.654921	-0.009123		
N	1.929046	-2.041281	-0.062575		
F	3.201281	-4.080114	-0.414045		
F	3.117847	-3.17402	1.695041		
H	0.319336	0.837523	-0.230609		
H	-1.234034	-1.355846	-0.587359		
H	0.427557	-3.492182	-0.352081		
H	5.931472	-3.442429	-0.209624		
H	7.583081	-1.280076	-0.312606		
H	5.991554	0.885273	0.017871		
H	3.128879	1.122451	0.179933		
O	3.754731	-0.065454	2.220865		
H	3.522406	-0.993356	2.403386		

NPA-ANION-C10-OH-TS

Atom	X	Y	Z		
O	2.415756	2.263422	0.069141	Electronic Energy (EE)	-741.949
O	2.408447	-2.247215	-0.103193	Zero-point Energy Correction	0.1616
N	2.455498	0.010361	-0.038308	Thermal Correction to Energy	0.173834
C	-0.415586	0.021683	0.043197	Thermal Correction to Enthalpy	0.174778
C	0.296859	1.23192	0.025367	Thermal Correction to Free Energy	0.122217
C	0.303384	-1.209984	0.130328		
C	-1.827117	0.017146	0.011403		
C	1.794258	1.187181	0.026261		
C	1.800809	-1.16704	-0.018611		
C	-0.394558	2.427318	-0.020455		

C	-0.402767	-2.414416	0.023682
C	-2.509212	1.255389	-0.031421
C	-2.505912	-1.233235	-0.008433
C	-1.801713	2.436781	-0.043275
C	-1.803819	-2.418923	-0.01573
H	0.153205	3.361568	-0.04294
H	0.149223	-3.346474	0.005588
H	-3.593484	1.257855	-0.058389
H	-3.590347	-1.235885	-0.035536
H	-2.327471	3.383158	-0.07814
H	-2.333112	-3.362528	-0.059139
O	0.463593	-1.295317	2.238736
H	0.835061	-2.190893	2.288514

NPA-ANION-C4-OH-TS

Atom	X	Y	Z		
O	2.410078	2.200256	0.144402	Electronic Energy (EE)	-741.948
O	2.388486	-2.308598	-0.086131	Zero-point Energy Correction	0.161856
N	2.442527	-0.052835	0.026369	Thermal Correction to Energy	0.173997
C	-0.427579	-0.041145	0.005692	Thermal Correction to Enthalpy	0.174941
C	0.28905	1.176607	0.045216	Thermal Correction to Free Energy	0.122482
C	0.283009	-1.259411	-0.022701		
C	-1.844778	-0.041325	-0.020351		
C	1.784921	1.127052	0.076		
C	1.778578	-1.22602	-0.028785		
C	-0.399036	2.369666	0.059669		
C	-0.413279	-2.470486	0.00219		
C	-2.519475	1.198162	-0.005218		
C	-2.532375	-1.293437	-0.066562		
C	-1.808299	2.37783	0.032509		
C	-1.840844	-2.467921	-0.068451		
H	0.146956	3.30454	0.091385		
H	0.128175	-3.395211	-0.141937		
H	-3.603825	1.207401	-0.025283		
H	-3.616264	-1.287723	-0.106518		
H	-2.333956	3.325024	0.0426		
H	-2.363348	-3.415894	-0.101115		
O	-0.063076	-2.673393	2.08545		
H	-0.650339	-1.946947	2.352043		

NPA-ANION-C5-OH-TS

Atom	X	Y	Z	Electronic Energy (EE)	-741.945
O	2.426987	2.288799	0.022448	Zero-point Energy Correction	0.161537
O	2.493184	-2.220571	-0.131118	Thermal Correction to Energy	0.173815

N	2.497762	0.034894	-0.055028	Thermal Correction to Enthalpy	0.174759
C	-0.370983	0.002079	0.006876	Thermal Correction to Free Energy	0.12176
C	0.323709	1.225881	0.000383		
C	0.36113	-1.21903	-0.012982		
C	-1.791107	-0.00979	0.019221		
C	1.820338	1.203095	-0.008558		
C	1.856706	-1.152946	-0.071602		
C	-0.381812	2.412695	-0.001396		
C	-0.303685	-2.411252	0.008564		
C	-2.486488	1.227632	0.010177		
C	-2.457091	-1.249944	0.029281		
C	-1.791863	2.412751	0.000907		
C	-1.732029	-2.445425	0.099825		
H	0.157207	3.352351	-0.007058		
H	0.24735	-3.343714	-0.013521		
H	-3.571013	1.217565	0.014227		
H	-3.54111	-1.274253	0.025473		
H	-2.324322	3.355823	-0.003552		
H	-2.244371	-3.384974	-0.058436		
O	-1.930084	-2.686319	2.156077		
H	-1.320423	-3.440866	2.175006		

NPA-ANION-C6-OH-TS

Atom	X	Y	Z	Electronic Energy (EE)	-741.949
O	2.434606	2.248802	-0.026186	Zero-point Energy Correction	0.161232
O	2.398538	-2.263106	0.04947	Thermal Correction to Energy	0.173612
N	2.455001	-0.006805	0.013137	Thermal Correction to Enthalpy	0.174556
C	-0.41514	0.020514	0.012742	Thermal Correction to Free Energy	0.121107
C	0.307357	1.23274	-0.008535		
C	0.289106	-1.21224	0.021232		
C	-1.827571	0.034691	0.003256		
C	1.80303	1.177176	-0.007155		
C	1.787344	-1.179147	0.029101		
C	-0.37375	2.431398	-0.030111		
C	-0.403276	-2.403753	0.010172		
C	-2.498693	1.274499	-0.018275		
C	-2.52453	-1.21398	0.047088		
C	-1.781812	2.451114	-0.035531		
C	-1.808276	-2.408286	-0.00026		
H	0.180232	3.362062	-0.044982		
H	0.139904	-3.340335	-0.000422		
H	-3.583349	1.286546	-0.020451		
H	-3.600524	-1.215804	-0.074845		
H	-2.301052	3.40165	-0.053108		
H	-2.34335	-3.350182	-0.022332		

O	-2.787346	-1.340405	2.207896		
H	-3.056517	-2.272811	2.249082		

NPA-C10-OH-TS-M

Atom	X	Y	Z		
O	2.450308	2.255798	0.02794	Electronic Energy (EE)	-742.421
O	2.449387	-2.250375	-0.105671	Zero-point Energy Correction	0.175157
N	2.389547	0.004485	-0.043293	Thermal Correction to Energy	0.187479
C	-0.396636	0.01524	0.039253	Thermal Correction to Enthalpy	0.188423
C	0.29678	1.239092	0.029711	Thermal Correction to Free Energy	0.136117
C	0.306496	-1.23121	0.138321		
C	-1.807881	0.00985	0.008151		
C	1.776171	1.242996	0.014847		
C	1.787291	-1.235758	-0.016591		
C	-0.397182	2.434206	-0.002383		
C	-0.40227	-2.435219	0.015655		
C	-2.495503	1.245634	-0.022616		
C	-2.491486	-1.237398	-0.015587		
C	-1.801187	2.435027	-0.022718		
C	-1.798826	-2.430516	-0.026347		
H	0.151697	3.368599	-0.017107		
H	0.150704	-3.367223	-0.008595		
H	-3.580048	1.239454	-0.047361		
H	-3.576003	-1.233005	-0.043757		
H	3.405554	0.009456	-0.100633		
H	-2.334836	3.377166	-0.045833		
H	-2.3352	-3.36975	-0.077137		
O	0.563854	-1.197465	2.177864		
H	0.663239	-2.148158	2.352126		

NPA-C10-OH-TS-P

Atom	X	Y	Z		
O	2.446415	2.259411	0.045213	Electronic Energy (EE)	-742.415
O	2.443972	-2.254314	-0.122144	Zero-point Energy Correction	0.175695
N	2.392122	0.004509	-0.045476	Thermal Correction to Energy	0.187942
C	-0.398297	0.014948	0.045043	Thermal Correction to Enthalpy	0.188886
C	0.295758	1.238168	0.032407	Thermal Correction to Free Energy	0.136779
C	0.307305	-1.230684	0.1492		
C	-1.809753	0.009106	0.011539		
C	1.780942	1.248578	0.023874		
C	1.791918	-1.241075	-0.020428		
C	-0.39654	2.43223	-0.004896		
C	-0.400671	-2.432758	0.020903		
C	-2.495976	1.245479	-0.025142		

C	-2.492944	-1.238915	-0.008778
C	-1.801211	2.433827	-0.028699
C	-1.799328	-2.429592	-0.019325
H	0.158266	3.362877	-0.019833
H	0.157447	-3.361509	-0.011891
H	-3.580493	1.241238	-0.050559
H	-3.577395	-1.236268	-0.036638
H	3.40679	0.009621	-0.095995
H	-2.334672	3.375972	-0.055579
H	-2.334545	-3.369562	-0.07036
O	0.553373	-1.195276	2.16234
H	0.661486	-2.145084	2.333461

NPA-C12-OH-TS

Atom	X	Y	Z		
O	2.449548	2.256984	0.02453	Electronic Energy (EE)	-742.418
O	2.446964	-2.250882	-0.088688	Zero-point Energy Correction	0.175055
N	2.387447	0.004857	-0.042243	Thermal Correction to Energy	0.187425
C	-0.398112	0.017013	0.043931	Thermal Correction to Enthalpy	0.188369
C	0.295566	1.240506	0.031589	Thermal Correction to Free Energy	0.135888
C	0.303813	-1.229762	0.146031		
C	-1.809092	0.012102	0.009884		
C	1.7739	1.243699	0.014403		
C	1.784012	-1.234775	-0.007		
C	-0.398329	2.43575	-0.004383		
C	-0.405573	-2.433513	0.019005		
C	-2.496591	1.247592	-0.024079		
C	-2.493564	-1.23465	-0.016713		
C	-1.802075	2.436963	-0.025594		
C	-1.801675	-2.428236	-0.027564		
H	0.150342	3.369733	-0.02221		
H	0.146804	-3.365466	-0.003056		
H	-3.580702	1.24048	-0.051099		
H	-3.577622	-1.228357	-0.047928		
H	3.402973	0.009055	-0.100409		
H	-2.335179	3.378826	-0.05179		
H	-2.338086	-3.366761	-0.081298		
O	0.544751	-1.217818	2.187889		
H	0.724447	-2.162412	2.32503		

NPA-C4-OH-TS-M

Atom	X	Y	Z		
O	2.444499	2.208374	0.093461	Electronic Energy (EE)	-742.42
O	2.421942	-2.300567	-0.016163	Zero-point Energy Correction	0.174769
				Thermal Correction to Energy	0.187257

N	2.37202	-0.044008	0.029225	Thermal Correction to Enthalpy	0.188201
C	-0.41027	-0.035186	0.00669	Thermal Correction to Free Energy	0.135132
C	0.287495	1.198362	0.039803		
C	0.280943	-1.266748	-0.017136		
C	-1.828537	-0.034827	-0.019014		
C	1.765313	1.198506	0.05813		
C	1.757392	-1.281014	-0.003196		
C	-0.403265	2.389607	0.052001		
C	-0.417293	-2.478256	0.006983		
C	-2.508491	1.202759	-0.004132		
C	-2.521853	-1.282912	-0.062903		
C	-1.810533	2.389543	0.030388		
C	-1.842876	-2.465042	-0.065257		
H	0.143633	3.324845	0.076438		
H	0.122858	-3.401134	-0.157724		
H	-3.593139	1.203012	-0.022176		
H	-3.605845	-1.268452	-0.102453		
H	3.389688	-0.047918	0.042992		
H	-2.343204	3.332628	0.040036		
H	-2.372915	-3.408738	-0.098531		
O	-0.060833	-2.739891	2.042201		
H	-0.640334	-2.03453	2.377667		

NPA-C4-OH-TS-P

Atom	X	Y	Z	Electronic Energy (EE)	-742.414
O	2.440926	2.216224	0.128724	Zero-point Energy Correction	0.175437
O	2.426829	-2.296492	-0.113401	Thermal Correction to Energy	0.187813
N	2.378579	-0.039877	0.029942	Thermal Correction to Enthalpy	0.188758
C	-0.407207	-0.034758	0.009383	Thermal Correction to Free Energy	0.135989
C	0.289352	1.198969	0.045601		
C	0.286529	-1.266678	-0.013504		
C	-1.825945	-0.034888	-0.018679		
C	1.773228	1.207177	0.075438		
C	1.767599	-1.284907	-0.036486		
C	-0.401597	2.389354	0.052931		
C	-0.408098	-2.477921	0.035362		
C	-2.505759	1.202194	-0.0112		
C	-2.517069	-1.285662	-0.056539		
C	-1.808503	2.389482	0.022476		
C	-1.836412	-2.465592	-0.044592		
H	0.151035	3.320917	0.080973		
H	0.138457	-3.397114	-0.124397		
H	-3.59034	1.202925	-0.03379		
H	-3.600893	-1.274798	-0.100254		
H	3.394819	-0.042181	0.029301		

H	-2.342115	3.332026	0.026267
H	-2.365395	-3.410153	-0.065065
O	-0.084849	-2.722126	2.043272
H	-0.726775	-2.06771	2.365567

NPA-C4-OH-TS

Atom	X	Y	Z	Electronic Energy (EE)	-742.417
O	2.445623	2.208617	0.092016	Zero-point Energy Correction	0.174911
O	2.425003	-2.30138	-0.020409	Thermal Correction to Energy	0.187316
N	2.373859	-0.044367	0.025337	Thermal Correction to Enthalpy	0.18826
C	-0.408084	-0.036906	0.007642	Thermal Correction to Free Energy	0.135446
C	0.288921	1.19698	0.041083		
C	0.283396	-1.267832	-0.017486		
C	-1.826211	-0.037319	-0.016947		
C	1.765676	1.19775	0.056857		
C	1.758897	-1.281154	-0.005897		
C	-0.402835	2.38762	0.055353		
C	-0.414884	-2.479643	0.00899		
C	-2.507177	1.1995	-0.000238		
C	-2.519456	-1.285169	-0.062942		
C	-1.810059	2.386697	0.03517		
C	-1.840273	-2.467065	-0.06584		
H	0.142853	3.323076	0.080106		
H	0.125503	-3.402404	-0.153218		
H	-3.591458	1.197976	-0.01766		
H	-3.603032	-1.269729	-0.103191		
H	3.391088	-0.047782	0.036858		
H	-2.342991	3.329116	0.046297		
H	-2.369699	-3.410587	-0.099786		
O	-0.077286	-2.726716	2.050859		
H	-0.660977	-2.010867	2.354376		

NPA-C5-OH-TS-M

Atom	X	Y	Z	Electronic Energy (EE)	-742.418
O	2.462114	2.291091	-0.025387	Zero-point Energy Correction	0.174669
O	2.529158	-2.214397	-0.089529	Thermal Correction to Energy	0.187275
N	2.427936	0.037677	-0.063226	Thermal Correction to Enthalpy	0.18822
C	-0.352799	0.005461	0.009484	Thermal Correction to Free Energy	0.134542
C	0.32266	1.244018	-0.000365		
C	0.360979	-1.229017	-0.007417		
C	-1.773315	-0.007795	0.021268		
C	1.800196	1.269486	-0.027072		
C	1.838871	-1.212207	-0.057137		
C	-0.385801	2.429551	0.000967		

C	-0.30612	-2.4211	0.013411
C	-2.473833	1.226742	0.014242
C	-2.444805	-1.245491	0.028252
C	-1.792536	2.419909	0.007802
C	-1.732288	-2.448097	0.105038
H	0.153472	3.369717	-0.007258
H	0.247096	-3.352962	-0.008989
H	-3.558486	1.207955	0.01846
H	-3.529113	-1.261419	0.019709
H	3.445201	0.054419	-0.090451
H	-2.3332	3.35817	0.006707
H	-2.251007	-3.383507	-0.058656
O	-1.887639	-2.679524	2.138675
H	-1.384108	-3.509657	2.165651

NPA-C5-OH-TS-P

Atom	X	Y	Z		
O	2.45682	2.258259	0.009941	Electronic Energy (EE)	-742.414
O	2.545616	-2.252499	-0.176959	Zero-point Energy Correction	0.175541
N	2.439268	0.001701	-0.07804	Thermal Correction to Energy	0.187912
C	-0.34467	-0.045062	0.006076	Thermal Correction to Enthalpy	0.188856
C	0.325402	1.196175	0.028552	Thermal Correction to Free Energy	0.135981
C	0.3748	-1.27779	-0.04968		
C	-1.763725	-0.067463	0.032891		
C	1.808413	1.236039	-0.010063		
C	1.858678	-1.25829	-0.108644		
C	-0.387395	2.377065	0.072162		
C	-0.285601	-2.472196	-0.060577		
C	-2.46895	1.161498	0.072806		
C	-2.429471	-1.314675	0.000533		
C	-1.793499	2.358683	0.093894		
C	-1.713543	-2.513331	0.027086		
H	0.152763	3.316341	0.086695		
H	0.277956	-3.396316	-0.104931		
H	-3.553418	1.138903	0.088721		
H	-3.513356	-1.33562	-0.005409		
H	3.454638	0.024808	-0.115818		
H	-2.340424	3.292798	0.126108		
H	-2.222958	-3.444986	-0.174869		
O	-1.969811	-2.907817	1.993032		
H	-1.524903	-2.1272	2.360671		

NPA-C5-OH-TS

Atom	X	Y	Z		
				Electronic Energy (EE)	-742.415

O	2.462572	2.292105	-0.028029	Zero-point Energy Correction	0.174606
O	2.528798	-2.214847	-0.08488	Thermal Correction to Energy	0.187218
N	2.42784	0.037988	-0.063665	Thermal Correction to Enthalpy	0.188162
C	-0.352589	0.006659	0.010929	Thermal Correction to Free Energy	0.134484
C	0.322934	1.244846	-0.001099		
C	0.360783	-1.228196	-0.004421		
C	-1.772897	-0.006158	0.023368		
C	1.799481	1.269752	-0.028492		
C	1.837785	-1.2116	-0.05454		
C	-0.385674	2.4305	-0.001426		
C	-0.306882	-2.419774	0.017627		
C	-2.473527	1.228069	0.014434		
C	-2.445209	-1.243309	0.031665		
C	-1.79219	2.421236	0.005863		
C	-1.733325	-2.446372	0.109182		
H	0.153235	3.370378	-0.011504		
H	0.244884	-3.352075	-0.002915		
H	-3.557799	1.208213	0.018623		
H	-3.529181	-1.257582	0.02158		
H	3.444637	0.054543	-0.091032		
H	-2.332418	3.359238	0.003143		
H	-2.251364	-3.38113	-0.057614		
O	-1.887608	-2.693032	2.140892		
H	-1.379657	-3.520429	2.14649		

NPA-C6-OH-TS-M

Atom	X	Y	Z	Electronic Energy (EE)	-742.421
O	2.465733	2.245448	-0.017005	Zero-point Energy Correction	0.174649
O	2.43324	-2.260948	0.042679	Thermal Correction to Energy	0.187141
N	2.382782	-0.006667	0.01768	Thermal Correction to Enthalpy	0.188086
C	-0.399603	0.018249	0.014589	Thermal Correction to Free Energy	0.134863
C	0.303589	1.245065	-0.008002		
C	0.286861	-1.228843	0.022912		
C	-1.811739	0.032179	0.00631		
C	1.781078	1.23883	-0.003349		
C	1.765953	-1.242599	0.029244		
C	-0.380612	2.442831	-0.031049		
C	-0.407129	-2.418933	0.008677		
C	-2.488556	1.268091	-0.015454		
C	-2.515336	-1.214827	0.057109		
C	-1.78526	2.453371	-0.035692		
C	-1.810146	-2.416721	-0.000765		
H	0.174363	3.373572	-0.046423		
H	0.137416	-3.355449	-0.006633		
H	-3.573498	1.270853	-0.014886		

H	-3.590768	-1.207469	-0.071518
H	3.400499	-0.013122	0.021884
H	-2.312762	3.399152	-0.053492
H	-2.351172	-3.354878	-0.027478
O	-2.743459	-1.326607	2.173221
H	-3.070312	-2.238749	2.254693

NPA-C6-OH-TS-P

Atom	X	Y	Z	Electronic Energy (EE)	-742.416
O	2.441228	2.215956	0.068756	Zero-point Energy Correction	0.175331
O	2.389459	-2.299942	0.051248	Thermal Correction to Energy	0.187764
N	2.354402	-0.040964	0.066221	Thermal Correction to Enthalpy	0.188708
C	-0.430756	-0.004704	0.016478	Thermal Correction to Free Energy	0.13573
C	0.278053	1.220022	0.023081		
C	0.251333	-1.253822	0.013148		
C	-1.844032	0.014582	-0.011119		
C	1.760992	1.214449	0.053845		
C	1.734399	-1.281042	0.043802		
C	-0.400354	2.419164	0.002247		
C	-0.449476	-2.440385	-0.028624		
C	-2.514019	1.254909	-0.029875		
C	-2.555775	-1.229748	0.013444		
C	-1.805744	2.436058	-0.027459		
C	-1.851823	-2.433487	-0.057898		
H	0.163972	3.344184	0.007385		
H	0.097348	-3.375386	-0.050407		
H	-3.598648	1.262933	-0.042528		
H	-3.624581	-1.216247	-0.156238		
H	3.370292	-0.052957	0.088959		
H	-2.32908	3.384107	-0.043661		
H	-2.39714	-3.367657	-0.10077		
O	-3.022048	-1.457264	2.036337		
H	-2.126839	-1.61093	2.380882		

NPA-C6-OH-TS

Atom	X	Y	Z	Electronic Energy (EE)	-742.418
O	2.467439	2.24698	-0.02194	Zero-point Energy Correction	0.174547
O	2.433934	-2.260747	0.042779	Thermal Correction to Energy	0.187076
N	2.383975	-0.00579	0.014536	Thermal Correction to Enthalpy	0.188021
C	-0.398156	0.019826	0.017461	Thermal Correction to Free Energy	0.134695
C	0.305127	1.246367	-0.008208		
C	0.287828	-1.227399	0.025678		
C	-1.810216	0.034115	0.010908		
C	1.781658	1.239613	-0.006257		

C	1.766004	-1.241334	0.029111
C	-0.379154	2.444143	-0.03276
C	-0.406822	-2.417281	0.013371
C	-2.487032	1.269902	-0.013564
C	-2.514507	-1.212242	0.062687
C	-1.783681	2.455053	-0.036511
C	-1.809632	-2.414639	0.005727
H	0.17542	3.374597	-0.050674
H	0.136764	-3.353861	-0.002146
H	-3.571624	1.271839	-0.01338
H	-3.589343	-1.203792	-0.067766
H	3.401231	-0.012512	0.016267
H	-2.310727	3.400546	-0.056349
H	-2.351202	-3.352097	-0.018261
O	-2.754833	-1.342684	2.178237
H	-3.081288	-2.256776	2.228307

NPA-N2-OH-TS-M

Atom	X	Y	Z		
O	2.234524	2.412098	0.258712	Electronic Energy (EE)	-742.395
O	2.398797	-2.095882	0.031735	Zero-point Energy Correction	0.170465
N	2.315465	0.159111	0.126166	Thermal Correction to Energy	0.183522
C	-0.500288	0.061554	0.021718	Thermal Correction to Enthalpy	0.184466
C	0.151525	1.301663	0.12321	Thermal Correction to Free Energy	0.127088
C	0.241607	-1.129995	-0.025734		
C	-1.917554	0.012751	-0.035082		
C	1.64607	1.350354	0.176832		
C	1.734919	-1.076046	0.046498		
C	-0.590967	2.491372	0.172841		
C	-0.410214	-2.36833	-0.130352		
C	-2.642233	1.216627	0.015968		
C	-2.550151	-1.238518	-0.139299		
C	-1.972956	2.447453	0.120504		
C	-1.791598	-2.41997	-0.186927		
H	-0.067478	3.436506	0.250391		
H	0.182234	-3.274494	-0.164657		
H	-3.725641	1.186203	-0.026859		
H	-3.632876	-1.284344	-0.183383		
H	3.41306	0.183157	0.169692		
H	-2.546963	3.364554	0.158636		
H	-2.296608	-3.374147	-0.267801		
O	4.943125	0.144152	0.139045		
H	5.240825	-0.769495	0.097076		

NPA-N2-OH-TS-P

Atom	X	Y	Z		
O	2.234524	2.412098	0.258712	Electronic Energy (EE)	-742.359
O	2.398797	-2.095882	0.031735	Zero-point Energy Correction	0.171326
N	2.315465	0.159111	0.126166	Thermal Correction to Energy	0.184013
C	-0.500288	0.061554	0.021718	Thermal Correction to Enthalpy	0.184957
C	0.151525	1.301663	0.12321	Thermal Correction to Free Energy	0.131428
C	0.241607	-1.129995	-0.025734		
C	-1.917554	0.012751	-0.035082		
C	1.64607	1.350354	0.176832		
C	1.734919	-1.076046	0.046498		
C	-0.590967	2.491372	0.172841		
C	-0.410214	-2.36833	-0.130352		
C	-2.642233	1.216627	0.015968		
C	-2.550151	-1.238518	-0.139299		
C	-1.972956	2.447453	0.120504		
C	-1.791598	-2.41997	-0.186927		
H	-0.067478	3.436506	0.250391		
H	0.182234	-3.274494	-0.164657		
H	-3.725641	1.186203	-0.026859		
H	-3.632876	-1.284344	-0.183383		
H	3.41306	0.183157	0.169692		
H	-2.546963	3.364554	0.158636		
H	-2.296608	-3.374147	-0.267801		
O	4.943125	0.144152	0.139045		
H	5.240825	-0.769495	0.097076		

NPA-N2-OH-TS

Atom	X	Y	Z		
O	2.443916	2.230046	-0.036142	Electronic Energy (EE)	-742.384
O	2.413633	-2.281181	-0.066971	Zero-point Energy Correction	0.170685
N	2.381441	-0.025277	-0.066749	Thermal Correction to Energy	0.183839
C	-0.407542	-0.006562	-0.025251	Thermal Correction to Enthalpy	0.184783
C	0.29367	1.217634	-0.029407	Thermal Correction to Free Energy	0.129363
C	0.276938	-1.240078	-0.040493		
C	-1.825257	0.00291	0.001162		
C	1.777878	1.211631	-0.046226		
C	1.761255	-1.254102	-0.059167		
C	-0.394824	2.426267	-0.010843		
C	-0.428275	-2.439269	-0.033431		
C	-2.49968	1.242253	0.021082		
C	-2.517102	-1.22718	0.009618		
C	-1.788581	2.437294	0.014808		
C	-1.822089	-2.43165	-0.007267		
H	0.163623	3.354288	-0.015042		

H	0.117378	-3.374819	-0.04561
H	-3.583894	1.249245	0.042535
H	-3.601345	-1.219025	0.03107
H	3.444038	-0.039031	-0.002045
H	-2.319258	3.380471	0.029904
H	-2.365339	-3.367736	0.000554
O	4.691945	-0.297275	0.900836
H	5.011059	-1.175557	1.149493

RDA-C'-TS-H

Atom	X	Y	Z
O	-0.09375	1.857266	0.059123
N	4.616787	2.121147	-0.498093
N	-4.796302	1.784372	0.109625
C	0.055048	-0.919547	0.454656
C	1.23584	-0.145057	0.38883
C	-1.186574	-0.283048	0.326284
C	1.132721	1.251863	0.050263
C	-1.23223	1.131618	0.16125
C	3.537641	1.453134	-0.234337
C	-3.629936	1.133342	0.170131
C	0.130937	-2.380106	0.702076
C	2.204867	2.022776	-0.237873
C	-2.410273	1.83233	0.093404
C	2.564985	-0.712793	0.385681
C	-2.441009	-0.964337	0.370917
C	3.647182	0.035046	0.097355
C	-3.619562	-0.291049	0.298411
C	0.698821	-3.230978	-0.248961
C	-0.372553	-2.90828	1.893804
C	0.755112	-4.600161	-0.011067
C	-0.298863	-4.276222	2.13378
C	0.261046	-5.123426	1.181038
H	2.073161	3.073384	-0.471254
H	-2.395152	2.908578	-0.02747
H	2.676118	-1.758836	0.641079
H	-2.446289	-2.043186	0.461279
H	4.641407	-0.396088	0.107319
H	-4.566749	-0.816292	0.331989
H	-0.811159	-2.243044	2.629848
H	1.187701	-5.256996	-0.756385
H	-0.681751	-4.67935	3.06379
H	0.312146	-6.189781	1.367219
H	4.375113	3.092709	-0.703499
H	-4.825271	2.788824	0.008567

Electronic Energy (EE)	-992.796
Zero-point Energy Correction	0.294949
Thermal Correction to Energy	0.314118
Thermal Correction to Enthalpy	0.315062
Thermal Correction to Free Energy	0.246874

H	-5.669854	1.279652	0.156338
H	1.086158	-2.819307	-1.174572
O	1.045579	0.221067	2.682243
H	1.316539	-0.678744	2.902761

RDA-C1-TS-H

Atom	X	Y	Z
O	-0.141417	1.834681	0.020769
N	4.599299	2.14421	-0.275655
N	-4.871805	1.821118	0.116741
C	0.025577	-0.934728	0.446741
C	1.169938	-0.190735	0.298036
C	-1.252554	-0.282384	0.358242
C	1.086081	1.235486	0.065423
C	-1.290089	1.108467	0.151905
C	3.504731	1.474411	-0.085103
C	-3.690104	1.139382	0.15968
C	0.084475	-2.395911	0.705968
C	2.167741	2.029723	-0.111372
C	-2.470122	1.819378	0.057469
C	2.503166	-0.730473	0.430497
C	-2.495517	-0.946497	0.438219
C	3.611537	0.045198	0.181197
C	-3.683165	-0.263444	0.345847
C	0.607961	-3.264677	-0.253982
C	-0.388018	-2.90857	1.917124
C	0.658067	-4.632633	-0.004178
C	-0.32412	-4.274838	2.168235
C	0.195756	-5.13888	1.207384
H	2.036308	3.092273	-0.280604
H	-2.440769	2.89067	-0.102824
H	2.620447	-1.796328	0.570141
H	-2.511256	-2.021624	0.568684
H	4.603389	-0.389831	0.211057
H	-4.628345	-0.790265	0.407795
H	-0.796394	-2.2337	2.661682
H	1.059961	-5.301374	-0.756209
H	-0.682725	-4.664216	3.113763
H	0.239346	-6.20398	1.402557
H	4.370486	3.124698	-0.448908
H	-4.862012	2.759452	-0.257261
H	-5.712081	1.289672	-0.061662
H	0.969346	-2.866168	-1.195801
O	2.946341	-0.257106	2.497049
H	3.735892	-0.81254	2.589902

Electronic Energy (EE)	-992.791
Zero-point Energy Correction	0.295328
Thermal Correction to Energy	0.314321
Thermal Correction to Enthalpy	0.315266
Thermal Correction to Free Energy	0.247425

RDA-C1-TS-M

Atom	X	Y	Z		
O	-0.122837	1.79939	-0.042968	Zero-point Energy Correction	0.308033
N	4.560891	2.225656	-0.215057	Thermal Correction to Energy	0.327478
N	-4.833247	1.806658	0.043678	Thermal Correction to Enthalpy	0.328422
C	0.005967	-0.94482	0.422842	Thermal Correction to Free Energy	0.259849
C	1.174722	-0.193555	0.263834		
C	-1.242672	-0.298439	0.331132		
C	1.080095	1.200293	0.03363		
C	-1.275323	1.100497	0.099836		
C	3.463969	1.474838	-0.055233		
C	-3.668847	1.145466	0.126007		
C	0.079444	-2.39976	0.701252		
C	2.179502	2.025585	-0.122153		
C	-2.443724	1.820246	0.001049		
C	2.501563	-0.727323	0.426075		
C	-2.500874	-0.957889	0.430593		
C	3.610827	0.070571	0.185273		
C	-3.670896	-0.268357	0.335307		
C	0.604378	-3.272846	-0.254493		
C	-0.376559	-2.897674	1.924339		
C	0.664132	-4.63674	0.010929		
C	-0.297723	-4.260359	2.190518		
C	0.217696	-5.130927	1.233809		
H	2.034411	3.085406	-0.292618		
H	-2.408097	2.888753	-0.174229		
H	2.631511	-1.798549	0.48966		
H	-2.522085	-2.030784	0.576442		
H	4.60736	-0.354217	0.224813		
H	-4.623311	-0.780596	0.409082		
H	-0.78043	-2.215942	2.665299		
H	1.061624	-5.312392	-0.737679		
H	-0.642854	-4.641358	3.144758		
H	0.271084	-6.193695	1.440929		
H	5.481562	1.813842	-0.160619		
H	4.489228	3.215314	-0.405137		
H	-4.855693	2.802824	-0.119381		
H	-5.71228	1.31674	0.12411		
H	0.953152	-2.883101	-1.205044		
O	2.719248	-0.470233	2.443821		
H	3.619865	-0.806268	2.587721		

RDA-C1-TS-P

Atom	X	Y	Z		
O	-0.119691	1.799641	-0.038915	Electronic Energy (EE)	-993.255
N	4.56388	2.22634	-0.220262	Zero-point Energy Correction	0.308192
N	-4.832584	1.802751	0.044827	Thermal Correction to Energy	0.327858
C	0.011203	-0.943926	0.429624	Thermal Correction to Enthalpy	0.328803
C	1.178148	-0.19117	0.273062	Thermal Correction to Free Energy	0.25957
C	-1.238209	-0.297166	0.332479		
C	1.082379	1.201247	0.040106		
C	-1.270527	1.10144	0.10194		
C	3.467175	1.47205	-0.054498		
C	-3.664839	1.143741	0.124502		
C	0.082558	-2.399168	0.70601		
C	2.183613	2.024309	-0.119513		
C	-2.440925	1.819815	0.001655		
C	2.506099	-0.72501	0.442677		
C	-2.49501	-0.95875	0.425495		
C	3.613802	0.069932	0.185149		
C	-3.665183	-0.270095	0.328503		
C	0.597483	-3.272067	-0.255107		
C	-0.367833	-2.898293	1.930536		
C	0.653954	-4.63661	0.006721		
C	-0.292052	-4.261365	2.192891		
C	0.213995	-5.13138	1.231187		
H	2.040212	3.083749	-0.291168		
H	-2.405923	2.888265	-0.17289		
H	2.627423	-1.797555	0.495706		
H	-2.513253	-2.031878	0.567634		
H	4.609559	-0.357429	0.214548		
H	-4.615857	-0.785753	0.396596		
H	-0.760073	-2.215907	2.676789		
H	1.042075	-5.312863	-0.74591		
H	-0.630646	-4.643168	3.148801		
H	0.264669	-6.194484	1.435654		
H	5.486	1.819652	-0.172973		
H	4.49226	3.214738	-0.411481		
H	-4.861018	2.799053	-0.110534		
H	-5.710547	1.312001	0.121262		
H	0.937921	-2.882549	-1.208645		
O	2.676984	-0.480959	2.430063		
H	3.587559	-0.77492	2.599607		

RDA-C11-TS-H

Atom	X	Y	Z		
O	-0.15167	1.708457	-0.337288	Electronic Energy (EE)	-992.776
N	4.569436	2.024125	-0.377876	Zero-point Energy Correction	0.296039
				Thermal Correction to Energy	0.314569

N	-4.847415	1.972011	0.307676	Thermal Correction to Enthalpy	0.315513
C	-0.151407	-1.023611	0.298045	Thermal Correction to Free Energy	0.249593
C	1.094498	-0.295853	0.153812		
C	-1.333935	-0.323689	0.300873		
C	1.041873	1.069755	-0.174509		
C	-1.325283	1.096552	0.001734		
C	3.432911	1.289833	-0.160003		
C	-3.736515	1.304756	0.2908		
C	-0.087714	-2.508789	0.499297		
C	2.168413	1.851718	-0.353688		
C	-2.437587	1.866507	-0.016136		
C	2.384374	-0.821696	0.390508		
C	-2.643574	-0.880142	0.593126		
C	3.520154	-0.061919	0.239524		
C	-3.75649	-0.123609	0.602048		
C	0.873624	-3.282554	-0.234911		
C	-0.528932	-3.03301	1.756186		
C	1.219395	-4.555101	0.175424		
C	-0.163707	-4.298545	2.154319		
C	0.69841	-5.072376	1.362231		
H	2.055583	2.895903	-0.620978		
H	-2.357331	2.917837	-0.269281		
H	2.494596	-1.844285	0.727206		
H	-2.714614	-1.932144	0.821998		
H	4.495582	-0.49212	0.435677		
H	-4.718068	-0.563187	0.842485		
H	-1.150664	-2.416573	2.394003		
H	1.904208	-5.144045	-0.421872		
H	-0.52491	-4.690444	3.09743		
H	0.97848	-6.067307	1.68702		
H	5.418619	1.684438	0.052232		
H	4.471061	3.029959	-0.382146		
H	-4.660091	2.94702	0.06916		
H	1.275029	-2.868967	-1.151971		
O	-1.468889	-3.051192	-0.785422		
H	-1.357691	-4.014182	-0.755745		

RDA-C11-TS-M

Atom	X	Y	Z		
				Electronic Energy (EE)	-993.263
O	-0.157187	1.714863	-0.211428	Zero-point Energy Correction	0.308667
N	4.531441	2.026507	-0.315157	Thermal Correction to Energy	0.327637
N	-4.825718	1.992376	0.243068	Thermal Correction to Enthalpy	0.328581
C	-0.114206	-1.022754	0.331179	Thermal Correction to Free Energy	0.262261
C	1.09205	-0.300529	0.205205		
C	-1.335109	-0.317742	0.328323		

C	1.031995	1.08949	-0.092344
C	-1.318178	1.078256	0.054566
C	3.41842	1.302557	-0.134104
C	-3.696607	1.270121	0.268386
C	-0.063542	-2.509791	0.512417
C	2.146695	1.873932	-0.284895
C	-2.451148	1.859182	0.008899
C	2.403961	-0.832952	0.406349
C	-2.623413	-0.882857	0.587018
C	3.519105	-0.071569	0.248019
C	-3.752674	-0.125965	0.571082
C	0.900146	-3.285965	-0.214897
C	-0.531815	-3.035217	1.75831
C	1.22645	-4.564005	0.192697
C	-0.18088	-4.305594	2.154301
C	0.684899	-5.082065	1.370029
H	2.029007	2.922273	-0.531437
H	-2.366856	2.914049	-0.22287
H	2.520875	-1.858431	0.730146
H	-2.703421	-1.936819	0.802845
H	4.503151	-0.491984	0.421613
H	-4.717675	-0.572887	0.781379
H	-1.155789	-2.418021	2.393392
H	1.911652	-5.15751	-0.400197
H	-0.559176	-4.699359	3.090238
H	0.950794	-6.081958	1.692722
H	5.443084	1.610033	-0.193027
H	4.481774	3.000925	-0.575665
H	-4.80929	2.975473	0.012824
H	-5.720302	1.556662	0.4138
H	1.311904	-2.872811	-1.12789
O	-1.425456	-2.973613	-0.812525
H	-1.338294	-3.940171	-0.82886

RDA-C11-TS-P

Atom	X	Y	Z		
O	-0.157254	1.717058	-0.20449	Electronic Energy (EE)	-993.248
N	4.533862	2.020992	-0.305757	Zero-point Energy Correction	0.309124
N	-4.826626	1.993065	0.238745	Thermal Correction to Energy	0.328131
C	-0.120138	-1.023514	0.328935	Thermal Correction to Enthalpy	0.329075
C	1.087891	-0.300513	0.20522	Thermal Correction to Free Energy	0.262493
C	-1.338158	-0.316434	0.326552		
C	1.029336	1.089634	-0.087918		
C	-1.318024	1.080624	0.05511		
C	3.417055	1.298498	-0.127582		

C	-3.697065	1.270025	0.264421
C	-0.06807	-2.510492	0.512293
C	2.147522	1.872435	-0.276661
C	-2.45191	1.860539	0.009077
C	2.397708	-0.835992	0.404862
C	-2.627044	-0.883516	0.578158
C	3.514558	-0.076249	0.24926
C	-3.75511	-0.126129	0.563559
C	0.886917	-3.286568	-0.224934
C	-0.513607	-3.030329	1.767877
C	1.218655	-4.56377	0.181791
C	-0.154115	-4.299091	2.165261
C	0.697951	-5.078106	1.370924
H	2.031805	2.921412	-0.519633
H	-2.36879	2.915287	-0.221521
H	2.510533	-1.86323	0.723577
H	-2.7023	-1.940757	0.777523
H	4.496894	-0.500801	0.421289
H	-4.721081	-0.573847	0.766117
H	-1.13228	-2.41167	2.406807
H	1.892049	-5.160523	-0.421079
H	-0.518103	-4.688824	3.108279
H	0.969434	-6.076785	1.69191
H	5.444462	1.603614	-0.186599
H	4.490565	2.994851	-0.566034
H	-4.813497	2.973892	0.002232
H	-5.722729	1.558729	0.400003
H	1.282534	-2.875511	-1.14587
O	-1.448391	-2.969934	-0.771579
H	-1.33477	-3.931939	-0.826617

RDA-C12-TS-H

Atom	X	Y	Z		
O	-0.098716	1.816664	-0.10608	Electronic Energy (EE)	-992.788
N	4.615914	2.207956	-0.327955	Zero-point Energy Correction	0.295869
N	-4.85066	1.760724	0.084649	Thermal Correction to Energy	0.314651
C	-0.005672	-0.929709	0.449838	Thermal Correction to Enthalpy	0.315595
C	1.210114	-0.177432	0.261906	Thermal Correction to Free Energy	0.24852
C	-1.217261	-0.293394	0.356954		
C	1.114271	1.196326	-0.016763		
C	-1.26465	1.126827	0.077725		
C	3.500378	1.445166	-0.101471		
C	-3.70068	1.166074	0.145431		
C	0.071715	-2.379804	0.747284		
C	2.221894	2.002885	-0.202038		

C	-2.419647	1.824733	-0.018919
C	2.510835	-0.711298	0.37653
C	-2.500788	-0.954967	0.478979
C	3.627696	0.06918	0.19664
C	-3.659522	-0.277754	0.383292
C	0.641045	-3.266343	-0.156935
C	-0.400687	-2.862815	1.996493
C	0.6857	-4.630863	0.131979
C	-0.391467	-4.253555	2.250059
C	0.165062	-5.124217	1.332542
H	2.088781	3.057549	-0.412927
H	-2.391234	2.888705	-0.226236
H	2.63344	-1.759966	0.618553
H	-2.514368	-2.026335	0.640344
H	4.618989	-0.359162	0.290232
H	-4.610224	-0.790842	0.473671
H	-0.984761	-2.206278	2.628822
H	1.119708	-5.313793	-0.588667
H	-0.80192	-4.61928	3.183357
H	0.192949	-6.187466	1.537268
H	5.490657	1.850261	0.030352
H	4.508544	3.210465	-0.258758
H	-4.707098	2.756522	-0.089456
H	1.033841	-2.896465	-1.097331
O	1.245286	-2.328397	3.140617
H	1.901576	-2.814539	2.615268

RDA-C12-TS-M

Atom	X	Y	Z		
O	-0.10385	1.812015	-0.05297	Electronic Energy (EE)	-993.273
N	4.580728	2.226876	-0.249732	Zero-point Energy Correction	0.307503
N	-4.811387	1.794365	0.072617	Thermal Correction to Energy	0.326393
C	0.039259	-0.930553	0.42599	Thermal Correction to Enthalpy	0.327338
C	1.214209	-0.181232	0.255307	Thermal Correction to Free Energy	0.260158
C	-1.208601	-0.287648	0.351562		
C	1.107714	1.215592	0.009926		
C	-1.248563	1.110936	0.107982		
C	3.487245	1.470389	-0.086283		
C	-3.64331	1.141746	0.152599		
C	0.111128	-2.384748	0.695613		
C	2.201696	2.030836	-0.164438		
C	-2.422886	1.822026	0.013068		
C	2.5342	-0.716063	0.349225		
C	-2.464589	-0.953483	0.464225		
C	3.629306	0.071955	0.182514		

C	-3.637751	-0.271647	0.373876
C	0.637095	-3.260551	-0.236361
C	-0.314	-2.870808	1.963471
C	0.686036	-4.631475	0.041033
C	-0.310205	-4.264491	2.19963
C	0.208562	-5.130727	1.253055
H	2.064037	3.089296	-0.349841
H	-2.395429	2.888912	-0.173197
H	2.659857	-1.768849	0.568258
H	-2.481786	-2.026232	0.615066
H	4.628155	-0.341909	0.259329
H	-4.587216	-0.787606	0.457973
H	-0.8796	-2.218009	2.617046
H	1.089495	-5.309537	-0.70199
H	-0.69401	-4.638239	3.14188
H	0.234029	-6.196398	1.447013
H	5.50298	1.820399	-0.187133
H	4.505642	3.216827	-0.435064
H	-4.841371	2.788865	-0.100154
H	-5.686918	1.299284	0.16094
H	0.992307	-2.885842	-1.190003
O	1.41087	-2.387036	2.956873
H	1.20208	-2.85	3.784717

RDA-C12-TS

Atom	X	Y	Z		
O	-0.109966	1.813304	-0.061242	Electronic Energy (EE)	-993.257
N	4.576347	2.22475	-0.235507	Zero-point Energy Correction	0.308484
N	-4.819764	1.795735	0.036965	Thermal Correction to Energy	0.327947
C	0.032265	-0.92301	0.457266	Thermal Correction to Enthalpy	0.328891
C	1.206069	-0.172545	0.290282	Thermal Correction to Free Energy	0.260777
C	-1.216003	-0.281131	0.359024		
C	1.099817	1.219664	0.020515		
C	-1.253667	1.114326	0.099651		
C	3.48023	1.471672	-0.063919		
C	-3.649512	1.144879	0.127568		
C	0.100931	-2.374726	0.740315		
C	2.196465	2.031103	-0.161385		
C	-2.428963	1.823548	-0.01134		
C	2.524417	-0.704267	0.410307		
C	-2.471924	-0.946554	0.465301		
C	3.620383	0.079636	0.235034		
C	-3.644423	-0.26607	0.359365		
C	0.630452	-3.258886	-0.186299		
C	-0.338018	-2.849252	2.007079		

C	0.665573	-4.62713	0.093965
C	-0.348911	-4.243871	2.24486
C	0.171758	-5.11657	1.305396
H	2.060688	3.085787	-0.365989
H	-2.399777	2.888303	-0.208142
H	2.642959	-1.749716	0.662111
H	-2.48817	-2.017991	0.623304
H	4.617776	-0.33208	0.334566
H	-4.592834	-0.783985	0.438813
H	-0.913559	-2.193885	2.647953
H	1.067518	-5.312801	-0.642513
H	-0.739552	-4.6109	3.186352
H	0.185909	-6.181563	1.503647
H	5.498034	1.820972	-0.16147
H	4.506876	3.210327	-0.440456
H	-4.853374	2.788262	-0.141086
H	-5.695292	1.301925	0.122473
H	0.996394	-2.888341	-1.13735
O	1.28724	-2.322823	3.059673
H	1.790767	-3.148853	2.978539

RDA-C13-TS-H

Atom	X	Y	Z		
O	-0.106635	1.846613	-0.087783	Electronic Energy (EE)	-992.761
N	4.622598	2.207194	-0.228014	Zero-point Energy Correction	0.294316
N	-4.802594	1.750274	0.006405	Thermal Correction to Energy	0.313028
C	0.044322	-0.90876	0.442706	Thermal Correction to Enthalpy	0.313972
C	1.22878	-0.136186	0.27241	Thermal Correction to Free Energy	0.246781
C	-1.187225	-0.27046	0.338765		
C	1.113845	1.249497	0.000999		
C	-1.242672	1.138181	0.073272		
C	3.52188	1.500102	-0.071945		
C	-3.634761	1.12338	0.10417		
C	0.139176	-2.357448	0.733693		
C	2.208545	2.057935	-0.174453		
C	-2.421307	1.825948	-0.036894		
C	2.541464	-0.684814	0.397772		
C	-2.441876	-0.956823	0.450855		
C	3.642423	0.092807	0.230819		
C	-3.617086	-0.294763	0.343273		
C	0.575418	-3.233364	-0.272305		
C	-0.233077	-2.810421	1.990512		
C	0.508602	-4.608918	-0.002312		
C	0.019064	-4.179745	2.361397		
C	0.159427	-5.085041	1.246159		

H	2.078339	3.113747	-0.382956
H	-2.416539	2.890238	-0.236521
H	2.638714	-1.737072	0.633066
H	-2.429041	-2.025865	0.619941
H	4.639896	-0.318159	0.327746
H	-4.564151	-0.815001	0.423326
H	-0.616242	-2.11198	2.72688
H	0.699276	-5.313367	-0.806653
H	-0.537103	-4.550678	3.21677
H	0.070146	-6.152198	1.418608
H	4.375185	3.179998	-0.428238
H	-4.84537	2.744202	-0.174682
H	-5.670635	1.24099	0.102801
H	0.848575	-2.858529	-1.251207
O	1.592528	-4.130778	3.220623
H	1.753478	-5.054293	3.453876

RDA-C13-TS-M

Atom	X	Y	Z		
O	-0.108128	1.818928	-0.098298	Electronic Energy (EE)	-993.271
N	4.579619	2.219062	-0.285319	Zero-point Energy Correction	0.307736
N	-4.814238	1.81621	0.024872	Thermal Correction to Energy	0.327228
C	0.028226	-0.909999	0.456768	Thermal Correction to Enthalpy	0.328172
C	1.204707	-0.164503	0.276076	Thermal Correction to Free Energy	0.259487
C	-1.217596	-0.269421	0.358198		
C	1.102487	1.22342	-0.01156		
C	-1.253993	1.124149	0.080238		
C	3.483081	1.470425	-0.10342		
C	-3.64875	1.162238	0.122707		
C	0.105685	-2.36323	0.748816		
C	2.199398	2.030983	-0.205023		
C	-2.426062	1.835517	-0.032682		
C	2.522175	-0.698539	0.396105		
C	-2.47639	-0.92827	0.487084		
C	3.620666	0.081275	0.211337		
C	-3.647209	-0.245338	0.379173		
C	0.619108	-3.244567	-0.219984		
C	-0.326441	-2.852485	1.966797		
C	0.666109	-4.614914	0.025826		
C	-0.188315	-4.231113	2.264111		
C	0.242808	-5.114554	1.245621		
H	2.065004	3.083721	-0.422625		
H	-2.3956	2.897506	-0.244713		
H	2.645293	-1.744236	0.651011		
H	-2.495248	-1.995862	0.667599		

H	4.618061	-0.331827	0.308762
H	-4.598269	-0.755953	0.476725
H	-0.724877	-2.18026	2.718765
H	1.027281	-5.28573	-0.744383
H	-0.680517	-4.629189	3.141897
H	0.268683	-6.17989	1.444286
H	5.500414	1.812383	-0.2057
H	4.508199	3.202913	-0.50154
H	-4.840657	2.806621	-0.170707
H	-5.691521	1.326855	0.126797
H	0.955092	-2.850277	-1.173089
O	1.577839	-4.094969	3.273122
H	1.74992	-5.048834	3.328211

RDA-C13-TS-P

Atom	X	Y	Z		
O	-0.097271	1.795585	-0.081743	Electronic Energy (EE)	-993.255
N	4.591259	2.183343	-0.266542	Zero-point Energy Correction	0.308055
N	-4.803381	1.81395	0.046634	Thermal Correction to Energy	0.327697
C	0.028552	-0.938645	0.461039	Thermal Correction to Enthalpy	0.328641
C	1.207302	-0.193987	0.281613	Thermal Correction to Free Energy	0.258964
C	-1.214548	-0.289983	0.36394		
C	1.109257	1.195711	0.001428		
C	-1.244021	1.105132	0.092425		
C	3.491412	1.436618	-0.089444		
C	-3.639178	1.154444	0.137198		
C	0.097622	-2.391966	0.752067		
C	2.21004	2.000492	-0.188206		
C	-2.413821	1.822123	-0.016369		
C	2.523367	-0.730608	0.399094		
C	-2.476546	-0.943407	0.487503		
C	3.624275	0.046086	0.218258		
C	-3.643718	-0.254181	0.384549		
C	0.63607	-3.274227	-0.198089		
C	-0.372383	-2.882013	1.961084		
C	0.675193	-4.645464	0.057018		
C	-0.252545	-4.258991	2.267432		
C	0.213044	-5.142647	1.263272		
H	2.078417	3.054335	-0.39994		
H	-2.377638	2.884838	-0.222734		
H	2.642713	-1.777657	0.647019		
H	-2.499136	-2.012053	0.659642		
H	4.619274	-0.371772	0.316404		
H	-4.595782	-0.762863	0.477954		
H	-0.788338	-2.206505	2.699984		

H	1.059245	-5.319956	-0.69873
H	-0.790619	-4.656073	3.117137
H	0.236091	-6.206573	1.465858
H	5.511023	1.774252	-0.197816
H	4.526384	3.166576	-0.483803
H	-4.829425	2.80422	-0.145371
H	-5.682942	1.328849	0.14113
H	0.996507	-2.886157	-1.144493
O	1.361909	-4.17272	3.441808
H	2.03838	-4.069862	2.753649

RDA-C14-TS-H

Atom	X	Y	Z		
				Electronic Energy (EE)	-992.785
O	-0.109862	1.826479	-0.023302	Zero-point Energy Correction	0.295166
N	4.627782	2.12565	-0.301583	Thermal Correction to Energy	0.314299
N	-4.842914	1.849008	0.131327	Thermal Correction to Enthalpy	0.315243
C	0.053009	-0.928465	0.471517	Thermal Correction to Free Energy	0.246832
C	1.201546	-0.199863	0.29262		
C	-1.227837	-0.271467	0.387769		
C	1.115452	1.221724	0.02641		
C	-1.260852	1.111357	0.14238		
C	3.538124	1.450286	-0.11202		
C	-3.660895	1.158666	0.174492		
C	0.106827	-2.382192	0.75845		
C	2.200187	2.00551	-0.171386		
C	-2.43924	1.826851	0.040815		
C	2.542101	-0.741834	0.392796		
C	-2.47294	-0.925722	0.493658		
C	3.632216	0.023419	0.200938		
C	-3.658763	-0.237486	0.395842		
C	0.652342	-3.266922	-0.18014		
C	-0.40348	-2.874011	1.974548		
C	0.71191	-4.620452	0.09517		
C	-0.33072	-4.217833	2.263981		
C	0.288235	-5.110717	1.353341		
H	2.071491	3.063845	-0.369257		
H	-2.404125	2.893159	-0.149368		
H	2.655555	-1.790869	0.637941		
H	-2.49725	-1.997563	0.648196		
H	4.627166	-0.399239	0.283059		
H	-4.605396	-0.758753	0.479544		
H	-0.848703	-2.18344	2.682009		
H	1.11581	-5.310157	-0.636636		
H	-0.713246	-4.597471	3.203576		
H	0.181398	-6.177184	1.499882		

H	4.392798	3.098826	-0.502145
H	-4.8265	2.767033	-0.290733
H	-5.679815	1.312736	-0.05042
H	1.011898	-2.883428	-1.128202
O	2.147082	-5.158976	2.245936
H	2.611853	-5.539356	1.484067

RDA-C14-TS-M

Atom	X	Y	Z
O	-0.101183	1.795444	-0.077941
N	4.587262	2.175802	-0.260247
N	-4.80751	1.816233	0.029004
C	0.02054	-0.93642	0.464508
C	1.201896	-0.197027	0.286677
C	-1.222549	-0.288812	0.365883
C	1.106158	1.193347	0.006274
C	-1.251066	1.105647	0.09362
C	3.487885	1.430946	-0.082374
C	-3.645742	1.155658	0.126765
C	0.087913	-2.38737	0.758825
C	2.206501	1.99701	-0.182538
C	-2.419154	1.823489	-0.019959
C	2.517585	-0.736343	0.407023
C	-2.484948	-0.942688	0.483324
C	3.619224	0.039879	0.226934
C	-3.65205	-0.253616	0.373821
C	0.62502	-3.267362	-0.188301
C	-0.398185	-2.873392	1.986059
C	0.693718	-4.619862	0.090056
C	-0.312408	-4.2163	2.277162
C	0.297307	-5.107388	1.358426
H	2.076369	3.051188	-0.395706
H	-2.382289	2.886331	-0.226601
H	2.637205	-1.783434	0.657091
H	-2.511076	-2.01171	0.65481
H	4.61476	-0.377819	0.323857
H	-4.605869	-0.760653	0.462604
H	-0.833642	-2.182069	2.69912
H	1.086558	-5.310028	-0.647661
H	-0.675882	-4.595499	3.224767
H	0.198909	-6.174392	1.510808
H	5.506482	1.765167	-0.182166
H	4.519801	3.160579	-0.47372
H	-4.828014	2.808064	-0.159999
H	-5.687772	1.330705	0.12339

Electronic Energy (EE)	-993.271
Zero-point Energy Correction	0.307731
Thermal Correction to Energy	0.327276
Thermal Correction to Enthalpy	0.32822
Thermal Correction to Free Energy	0.259329

H	0.965745	-2.884766	-1.143973
O	2.157235	-5.122563	2.214945
H	2.614329	-5.580807	1.491321

RDA-C14-TS-P

Atom	X	Y	Z
O	-0.086602	1.778458	-0.062273
N	4.599082	2.161969	-0.29357
N	-4.791163	1.808876	0.110267
C	0.03757	-0.964321	0.437707
C	1.217315	-0.220269	0.254504
C	-1.205531	-0.310328	0.361584
C	1.118994	1.174139	-0.002816
C	-1.233597	1.088477	0.111443
C	3.500523	1.413707	-0.116584
C	-3.628157	1.144298	0.175574
C	0.103833	-2.417796	0.7142
C	2.21878	1.980161	-0.192372
C	-2.402036	1.810884	0.024627
C	2.534538	-0.759632	0.349484
C	-2.468672	-0.962931	0.477476
C	3.634382	0.018497	0.168381
C	-3.634635	-0.269089	0.393113
C	0.671749	-3.287978	-0.229387
C	-0.41122	-2.924971	1.917331
C	0.729823	-4.643574	0.026797
C	-0.34001	-4.27852	2.18555
C	0.284993	-5.158209	1.268839
H	2.086027	3.037084	-0.387472
H	-2.363753	2.876724	-0.164583
H	2.657265	-1.810594	0.578101
H	-2.494682	-2.034932	0.626034
H	4.629485	-0.402948	0.247913
H	-4.587412	-0.778068	0.478007
H	-0.860369	-2.247452	2.634882
H	1.14975	-5.320518	-0.706867
H	-0.731837	-4.671572	3.115615
H	0.157043	-6.225485	1.38669
H	5.518948	1.749945	-0.246669
H	4.53269	3.147467	-0.500043
H	-4.816143	2.802531	-0.063635
H	-5.671398	1.323528	0.197334
H	1.042151	-2.891409	-1.168121
O	2.063038	-5.37302	2.182872
H	2.248304	-4.437966	2.365955

Electronic Energy (EE)	-993.255
Zero-point Energy Correction	0.308256
Thermal Correction to Energy	0.327793
Thermal Correction to Enthalpy	0.328737
Thermal Correction to Free Energy	0.259924

RDA-C2-TS-H

Atom	X	Y	Z		
O	-0.096364	1.837489	-0.043233	Electronic Energy (EE)	-992.794
N	4.61859	2.102548	-0.519737	Zero-point Energy Correction	0.29506
N	-4.790804	1.784237	0.142573	Thermal Correction to Energy	0.314164
C	0.053481	-0.930969	0.421048	Thermal Correction to Enthalpy	0.315108
C	1.239514	-0.176013	0.257494	Thermal Correction to Free Energy	0.246952
C	-1.184883	-0.284844	0.351465		
C	1.13396	1.23909	-0.001392		
C	-1.232073	1.12595	0.124244		
C	3.540086	1.463394	-0.190092		
C	-3.627398	1.136111	0.200259		
C	0.129257	-2.390653	0.674886		
C	2.210523	2.031566	-0.216455		
C	-2.407974	1.826391	0.054266		
C	2.534368	-0.722065	0.366947		
C	-2.443273	-0.957783	0.466924		
C	3.651059	0.074071	0.246728		
C	-3.618144	-0.283149	0.400323		
C	0.672779	-3.243734	-0.288998		
C	-0.343586	-2.9171	1.879471		
C	0.732765	-4.612431	-0.051323		
C	-0.265975	-4.284817	2.11907		
C	0.267847	-5.133895	1.153412		
H	2.078176	3.083066	-0.444463		
H	-2.394125	2.895497	-0.118231		
H	2.649812	-1.77103	0.605555		
H	-2.450414	-2.031533	0.602308		
H	4.641725	-0.358172	0.273933		
H	-4.566449	-0.800318	0.485434		
H	-0.761591	-2.252357	2.627598		
H	1.145358	-5.27064	-0.806682		
H	-0.625256	-4.685922	3.059292		
H	0.321753	-6.20015	1.339275		
H	4.378773	3.058802	-0.79078		
H	-4.821802	2.782521	-0.011931		
H	-5.663831	1.284961	0.240698		
H	1.037703	-2.833486	-1.224223		
O	3.950504	0.753361	2.502282		
H	3.659065	-0.113791	2.802351		

RDA-C2-TS-M

Atom	X	Y	Z	Electronic Energy (EE)	-993.277

O	-0.093657	1.806803	-0.080694	Zero-point Energy Correction	0.308079
N	4.581309	2.222823	-0.350407	Thermal Correction to Energy	0.327472
N	-4.791453	1.796431	0.125618	Thermal Correction to Enthalpy	0.328416
C	0.045739	-0.939945	0.42294	Thermal Correction to Free Energy	0.259944
C	1.22693	-0.182886	0.239837		
C	-1.194167	-0.295353	0.354411		
C	1.118447	1.214269	-0.026607		
C	-1.235973	1.106748	0.104461		
C	3.492784	1.481354	-0.15204		
C	-3.628863	1.141039	0.187738		
C	0.126737	-2.395695	0.689807		
C	2.204178	2.031028	-0.232428		
C	-2.406656	1.819007	0.025825		
C	2.531157	-0.714272	0.329816		
C	-2.45454	-0.95767	0.484215		
C	3.645032	0.091173	0.197085		
C	-3.625169	-0.273837	0.409967		
C	0.672916	-3.254656	-0.267751		
C	-0.344557	-2.912324	1.89959		
C	0.736121	-4.621083	-0.018219		
C	-0.260737	-4.277356	2.150925		
C	0.274292	-5.133009	1.191643		
H	2.058292	3.082727	-0.446719		
H	-2.380877	2.885075	-0.164989		
H	2.667709	-1.766418	0.54591		
H	-2.470535	-2.029462	0.63651		
H	4.635799	-0.343634	0.166679		
H	-4.575564	-0.785768	0.506433		
H	-0.763567	-2.24263	2.643059		
H	1.148771	-5.28511	-0.768965		
H	-0.617324	-4.671663	3.095465		
H	0.331311	-6.197855	1.386691		
H	5.503575	1.817408	-0.269719		
H	4.508801	3.209236	-0.55876		
H	-4.820259	2.791835	-0.04542		
H	-5.668119	1.306001	0.232716		
H	1.033388	-2.852199	-1.20844		
O	3.914009	0.623762	2.299926		
H	3.732192	-0.260633	2.658284		

RDA-C2-TS-P

Atom	X	Y	Z	Electronic Energy (EE)	-993.26
O	-0.092846	1.802221	-0.081984	Zero-point Energy Correction	0.308633
N	4.581939	2.220719	-0.333651	Thermal Correction to Energy	0.328081
N	-4.794126	1.797758	0.129523	Thermal Correction to Enthalpy	0.329025

C	0.048166	-0.942727	0.419492	Thermal Correction to Free Energy	0.26034
C	1.226963	-0.186307	0.242134		
C	-1.194759	-0.296635	0.349663		
C	1.117322	1.211754	-0.025636		
C	-1.234855	1.103635	0.101424		
C	3.491624	1.483174	-0.138167		
C	-3.628221	1.141375	0.18651		
C	0.1274	-2.398221	0.68855		
C	2.204276	2.028341	-0.23261		
C	-2.406628	1.817763	0.024432		
C	2.532753	-0.720443	0.324701		
C	-2.453567	-0.958096	0.479006		
C	3.649451	0.090085	0.219153		
C	-3.624132	-0.272331	0.405893		
C	0.663382	-3.261532	-0.270602		
C	-0.334188	-2.909952	1.904156		
C	0.726863	-4.626845	-0.016393		
C	-0.249892	-4.273851	2.15992		
C	0.275499	-5.133299	1.199294		
H	2.057376	3.079248	-0.447782		
H	-2.378105	2.883974	-0.16472		
H	2.666273	-1.776955	0.519798		
H	-2.468579	-2.029821	0.631251		
H	4.632566	-0.352532	0.131614		
H	-4.573143	-0.786339	0.501726		
H	-0.745103	-2.236555	2.648732		
H	1.130402	-5.295049	-0.767997		
H	-0.597726	-4.664228	3.108998		
H	0.332662	-6.19718	1.397694		
H	5.501091	1.829618	-0.183427		
H	4.515385	3.213409	-0.508263		
H	-4.827061	2.792404	-0.038716		
H	-5.670398	1.307739	0.23182		
H	1.014127	-2.863914	-1.216812		
O	4.028912	0.584845	2.189505		
H	3.606366	-0.171986	2.626164		

RDA-C3-TS-H

Atom	X	Y	Z		
				Electronic Energy (EE)	-992.799
O	-0.008976	1.777417	-0.030678	Zero-point Energy Correction	0.294802
N	4.69389	2.021817	-0.5094	Thermal Correction to Energy	0.314059
N	-4.708526	1.850659	0.200441	Thermal Correction to Enthalpy	0.315003
C	0.090011	-0.975274	0.420571	Thermal Correction to Free Energy	0.245942
C	1.278085	-0.239677	0.22795		
C	-1.142657	-0.305801	0.373131		

C	1.185458	1.144157	-0.00779
C	-1.164048	1.101501	0.146742
C	3.613758	1.3548	-0.228141
C	-3.552942	1.178254	0.244364
C	0.143178	-2.435275	0.672536
C	2.301217	1.953036	-0.174667
C	-2.324074	1.836984	0.087047
C	2.605382	-0.81238	0.296937
C	-2.412212	-0.948021	0.501225
C	3.70643	-0.065595	0.08127
C	-3.570369	-0.240909	0.443317
C	0.683511	-3.293209	-0.289089
C	-0.346667	-2.957302	1.872342
C	0.722933	-4.662865	-0.054033
C	-0.288131	-4.326227	2.109688
C	0.241822	-5.180147	1.146212
H	2.17406	3.000788	-0.412351
H	-2.280051	2.905093	-0.085684
H	2.699848	-1.86436	0.532222
H	-2.446857	-2.021234	0.635916
H	4.696442	-0.503549	0.124235
H	-4.529443	-0.737061	0.535801
H	-0.761173	-2.288669	2.618869
H	1.132678	-5.325034	-0.807406
H	-0.659446	-4.72454	3.046358
H	0.280498	-6.247333	1.33043
H	4.45397	2.999166	-0.690946
H	-4.721447	2.847846	0.041383
H	-5.589144	1.367012	0.300591
H	1.061097	-2.885858	-1.220662
O	2.417307	2.648393	2.2168
H	1.647398	2.103272	2.409473

RDA-C3-TS-M

Atom	X	Y	Z		
				Electronic Energy (EE)	-993.269
O	-0.107787	1.804836	-0.0153	Zero-point Energy Correction	0.308287
N	4.564677	2.124348	-0.526998	Thermal Correction to Energy	0.327466
N	-4.80972	1.778667	0.077563	Thermal Correction to Enthalpy	0.32841
C	0.032759	-0.944045	0.45322	Thermal Correction to Free Energy	0.260689
C	1.212407	-0.183282	0.293998		
C	-1.208281	-0.299816	0.368807		
C	1.097549	1.199819	0.052693		
C	-1.251473	1.10733	0.131331		
C	3.513221	1.468556	-0.011601		
C	-3.643536	1.133369	0.160217		

C	0.108128	-2.404161	0.70243
C	2.196066	2.023385	-0.124741
C	-2.423217	1.817392	0.031798
C	2.544593	-0.729677	0.393942
C	-2.468371	-0.967865	0.467057
C	3.639078	0.041622	0.24098
C	-3.637631	-0.285656	0.371891
C	0.657229	-3.250761	-0.264044
C	-0.373666	-2.936417	1.900815
C	0.713131	-4.621	-0.034758
C	-0.298927	-4.305797	2.131659
C	0.239554	-5.149153	1.163644
H	2.063622	3.07325	-0.355413
H	-2.396056	2.885218	-0.148124
H	2.657065	-1.786321	0.599529
H	-2.484432	-2.040726	0.610108
H	4.638281	-0.372161	0.31169
H	-4.588483	-0.801166	0.442834
H	-0.796017	-2.276333	2.651039
H	1.128802	-5.27534	-0.792307
H	-0.665208	-4.712859	3.067022
H	0.289971	-6.217124	1.342729
H	5.490602	1.757136	-0.35029
H	4.497422	3.125675	-0.656043
H	-4.843684	2.775337	-0.085895
H	-5.684348	1.28001	0.162486
H	1.027164	-2.835702	-1.195694
O	3.395637	2.183082	1.842497
H	2.788037	1.623555	2.350446

RDA-C3-TS-P

Atom	X	Y	Z		
O	-0.108989	1.803694	-0.00945	Electronic Energy (EE)	-993.251
N	4.563948	2.126251	-0.535848	Zero-point Energy Correction	0.309031
N	-4.81559	1.781646	0.080613	Thermal Correction to Energy	0.32803
C	0.032762	-0.946446	0.448773	Thermal Correction to Enthalpy	0.328974
C	1.208966	-0.187164	0.289318	Thermal Correction to Free Energy	0.261859
C	-1.211816	-0.299932	0.364737		
C	1.095404	1.200224	0.056403		
C	-1.252969	1.104829	0.131376		
C	3.511514	1.469104	-0.00988		
C	-3.646366	1.133868	0.15815		
C	0.106563	-2.40593	0.700217		
C	2.195669	2.024558	-0.111963		
C	-2.425975	1.816422	0.031867		

C	2.541927	-0.732446	0.384093
C	-2.470133	-0.967265	0.45786
C	3.635358	0.039823	0.229273
C	-3.640109	-0.283679	0.363086
C	0.656016	-3.256008	-0.26292
C	-0.374788	-2.934408	1.900677
C	0.713665	-4.624923	-0.027831
C	-0.296908	-4.302108	2.137703
C	0.242455	-5.148256	1.17314
H	2.064163	3.075418	-0.335461
H	-2.396735	2.884603	-0.14496
H	2.653884	-1.78993	0.584631
H	-2.48505	-2.040619	0.596182
H	4.63361	-0.376103	0.295318
H	-4.589322	-0.802365	0.429075
H	-0.796948	-2.271774	2.648616
H	1.12852	-5.282483	-0.782608
H	-0.660144	-4.705654	3.075413
H	0.295109	-6.214957	1.357039
H	5.486714	1.772233	-0.321439
H	4.501356	3.132929	-0.612286
H	-4.85261	2.77812	-0.076039
H	-5.690278	1.284361	0.160114
H	1.023382	-2.84517	-1.197257
O	3.390953	2.200191	1.802713
H	2.84695	1.596571	2.332767

RDA-C4'-TS-H

Atom	X	Y	Z		
				Electronic Energy (EE)	-992.796
O	-0.106144	1.77934	-0.127706	Zero-point Energy Correction	0.295499
N	4.612983	2.161541	-0.295433	Thermal Correction to Energy	0.314301
N	-4.831542	1.82789	0.160852	Thermal Correction to Enthalpy	0.315245
C	0.057877	-0.949227	0.453928	Thermal Correction to Free Energy	0.248265
C	1.204386	-0.207876	0.291283		
C	-1.217214	-0.29117	0.400959		
C	1.089052	1.214298	0.098673		
C	-1.259774	1.084164	0.114323		
C	3.535376	1.467211	-0.090551		
C	-3.652653	1.143132	0.199823		
C	0.122722	-2.414479	0.687236		
C	2.205709	2.019845	-0.155515		
C	-2.433848	1.803025	0.009568		
C	2.552575	-0.73047	0.398881		
C	-2.464073	-0.937829	0.561904		
C	3.63938	0.043751	0.226358		

C	-3.645747	-0.247243	0.473203
C	0.640387	-3.260762	-0.295649
C	-0.327106	-2.952744	1.895583
C	0.70231	-4.632796	-0.073173
C	-0.251249	-4.32329	2.119267
C	0.259876	-5.165321	1.134659
H	2.057984	3.066918	-0.39396
H	-2.398693	2.861417	-0.219515
H	2.67158	-1.781776	0.630974
H	-2.486559	-2.003209	0.754078
H	4.636958	-0.369819	0.31636
H	-4.592079	-0.759793	0.602186
H	-0.725951	-2.295051	2.660347
H	1.099062	-5.284149	-0.842972
H	-0.592738	-4.732754	3.062644
H	0.313232	-6.233604	1.30885
H	4.361071	3.13095	-0.497963
H	-4.833856	2.743174	-0.266542
H	-5.681966	1.293178	0.055306
H	0.988361	-2.841637	-1.233553
O	1.432072	1.864396	2.098077
H	1.131999	2.780447	2.01476

RDA-C4'-TS-M

Atom	X	Y	Z		
				Electronic Energy (EE)	-993.27
O	-0.10032	1.768515	-0.159539	Zero-point Energy Correction	0.308604
N	4.582659	2.198128	-0.257531	Thermal Correction to Energy	0.327643
N	-4.810382	1.79712	0.069484	Thermal Correction to Enthalpy	0.328587
C	0.045221	-0.953376	0.463707	Thermal Correction to Free Energy	0.26137
C	1.206641	-0.206399	0.303585		
C	-1.210469	-0.297203	0.403329		
C	1.086728	1.216805	0.131473		
C	-1.251811	1.082124	0.090627		
C	3.508285	1.444284	-0.054917		
C	-3.645143	1.134096	0.1636		
C	0.11622	-2.417839	0.693764		
C	2.216447	2.019357	-0.143432		
C	-2.420922	1.795042	-0.03423		
C	2.532329	-0.723561	0.411232		
C	-2.465327	-0.944784	0.566483		
C	3.637342	0.053958	0.247286		
C	-3.640626	-0.260074	0.461064		
C	0.624482	-3.258804	-0.298808		
C	-0.319157	-2.95807	1.906391		
C	0.687423	-4.631409	-0.081555		

C	-0.239283	-4.329251	2.123907
C	0.259335	-5.167484	1.129812
H	2.079737	3.064121	-0.397003
H	-2.387521	2.849732	-0.280083
H	2.659118	-1.776243	0.634593
H	-2.486894	-2.00718	0.77524
H	4.632158	-0.365738	0.336013
H	-4.589381	-0.76753	0.592691
H	-0.707837	-2.30267	2.678598
H	1.073783	-5.280532	-0.858962
H	-0.568896	-4.742207	3.070363
H	0.314333	-6.236765	1.299591
H	5.510438	1.795555	-0.218294
H	4.498108	3.18506	-0.46526
H	-4.832338	2.780923	-0.155229
H	-5.688496	1.318966	0.206572
H	0.959311	-2.837055	-1.240588
O	1.365406	1.843669	1.972796
H	1.092112	2.774572	1.923079

RDA-C4'-TS-P

Atom	X	Y	Z		
O	-0.102278	1.772367	-0.167315	Electronic Energy (EE)	-993.253
N	4.587335	2.204905	-0.232957	Zero-point Energy Correction	0.308919
N	-4.812335	1.791895	0.055952	Thermal Correction to Energy	0.328034
C	0.043874	-0.951741	0.452172	Thermal Correction to Enthalpy	0.328978
C	1.206104	-0.204959	0.287064	Thermal Correction to Free Energy	0.261573
C	-1.209312	-0.293111	0.399643		
C	1.085839	1.222017	0.132148		
C	-1.250154	1.086618	0.084583		
C	3.505048	1.449065	-0.045782		
C	-3.644612	1.132618	0.155511		
C	0.11481	-2.415455	0.686673		
C	2.216434	2.023403	-0.134644		
C	-2.421923	1.79523	-0.043947		
C	2.527056	-0.724583	0.387943		
C	-2.463107	-0.94201	0.568194		
C	3.632964	0.056506	0.233719		
C	-3.638653	-0.259622	0.461656		
C	0.593599	-3.265467	-0.312378		
C	-0.289433	-2.944241	1.914889		
C	0.658817	-4.636188	-0.085337		
C	-0.207056	-4.313485	2.141326		
C	0.262719	-5.160712	1.141405		
H	2.083094	3.071081	-0.378006		

H	-2.39088	2.848705	-0.294411
H	2.651181	-1.779912	0.599367
H	-2.479865	-2.003794	0.780257
H	4.626324	-0.366152	0.321674
H	-4.586143	-0.767983	0.596603
H	-0.653419	-2.280549	2.691925
H	1.021115	-5.293233	-0.867306
H	-0.510897	-4.717892	3.099687
H	0.319796	-6.22834	1.318649
H	5.512732	1.800557	-0.196376
H	4.511566	3.194789	-0.420738
H	-4.840279	2.77192	-0.180602
H	-5.689743	1.313043	0.189365
H	0.902221	-2.852889	-1.266862
O	1.324363	1.787239	1.979099
H	1.12591	2.738209	1.957007

RDA-C4-TS-H

Atom	X	Y	Z		
O	-0.008973	1.777425	-0.030626	Electronic Energy (EE)	-992.799
N	4.693888	2.021834	-0.50939	Zero-point Energy Correction	0.294802
N	-4.708526	1.850655	0.200433	Thermal Correction to Energy	0.314059
C	0.090012	-0.975277	0.420562	Thermal Correction to Enthalpy	0.315003
C	1.278086	-0.23968	0.227934	Thermal Correction to Free Energy	0.245941
C	-1.142655	-0.305801	0.373138		
C	1.18546	1.144161	-0.007765		
C	-1.164046	1.101504	0.146766		
C	3.613757	1.354804	-0.228147		
C	-3.552941	1.178252	0.244361		
C	0.143176	-2.435277	0.672532		
C	2.301219	1.953044	-0.174623		
C	-2.324072	1.836986	0.087068		
C	2.605382	-0.812392	0.296869		
C	-2.41221	-0.948024	0.501214		
C	3.706428	-0.065608	0.08119		
C	-3.570368	-0.240914	0.443302		
C	0.683512	-3.293223	-0.289081		
C	-0.346676	-2.957291	1.872342		
C	0.722935	-4.662876	-0.054007		
C	-0.288139	-4.326212	2.109705		
C	0.24182	-5.180144	1.146242		
H	2.17406	3.000798	-0.412294		
H	-2.28005	2.905097	-0.08565		
H	2.69985	-1.864378	0.532125		
H	-2.446856	-2.021239	0.635891		

H	4.696439	-0.503568	0.124111
H	-4.529442	-0.737068	0.535769
H	-0.761184	-2.28865	2.61886
H	1.132684	-5.325054	-0.80737
H	-0.659458	-4.724514	3.046378
H	0.280498	-6.247327	1.330474
H	4.453956	2.999191	-0.690878
H	-4.721448	2.84784	0.04136
H	-5.589145	1.367001	0.300542
H	1.0611	-2.885884	-1.220658
O	2.417302	2.648434	2.216825
H	1.647436	2.103251	2.409494

RDA-C4-TS-M

Atom	X	Y	Z		
O	-0.087984	1.810818	-0.075487	Electronic Energy (EE)	-993.28
N	4.587773	2.217028	-0.353272	Zero-point Energy Correction	0.30787
N	-4.79197	1.814657	0.091744	Thermal Correction to Energy	0.327408
C	0.048548	-0.929706	0.428143	Thermal Correction to Enthalpy	0.328352
C	1.224936	-0.176186	0.238314	Thermal Correction to Free Energy	0.259407
C	-1.19517	-0.282614	0.35743		
C	1.113347	1.208461	-0.016922		
C	-1.235076	1.11599	0.10267		
C	3.508471	1.463931	-0.150807		
C	-3.627258	1.158742	0.166613		
C	0.125003	-2.38448	0.701357		
C	2.216233	2.038309	-0.174412		
C	-2.404719	1.832705	0.011351		
C	2.550067	-0.70795	0.326056		
C	-2.455654	-0.941173	0.483038		
C	3.647523	0.069925	0.135702		
C	-3.625025	-0.254013	0.396696		
C	0.67787	-3.248319	-0.247858		
C	-0.35702	-2.895169	1.909485		
C	0.736834	-4.613775	0.008		
C	-0.277246	-4.259146	2.16748		
C	0.264271	-5.119706	1.216244		
H	2.074854	3.079235	-0.437248		
H	-2.373275	2.897797	-0.184054		
H	2.676693	-1.757435	0.560595		
H	-2.475986	-2.012177	0.640134		
H	4.644855	-0.348828	0.201806		
H	-4.576305	-0.76519	0.489303		
H	-0.78077	-2.221652	2.646787		
H	1.154966	-5.281694	-0.736216		

H	-0.64182	-4.648818	3.110888
H	0.318162	-6.183763	1.416438
H	5.514012	1.815156	-0.305241
H	4.504949	3.207106	-0.540121
H	-4.819604	2.808143	-0.088193
H	-5.668878	1.32387	0.191546
H	1.047125	-2.850359	-1.187084
O	2.412115	2.556868	2.075327
H	1.504723	2.904917	2.099736

RDA-C4-TS-P

Atom	X	Y	Z		
O	-0.127399	1.803804	-0.075858	Electronic Energy (EE)	-993.262
N	4.539967	2.230426	-0.378707	Zero-point Energy Correction	0.309103
N	-4.835299	1.772515	0.041294	Thermal Correction to Energy	0.328457
C	0.023529	-0.9358	0.429525	Thermal Correction to Enthalpy	0.329402
C	1.195845	-0.173764	0.245981	Thermal Correction to Free Energy	0.261011
C	-1.223992	-0.29515	0.349547		
C	1.075638	1.207879	-0.00565		
C	-1.270864	1.102	0.088818		
C	3.473492	1.468479	-0.158635		
C	-3.664275	1.126947	0.128086		
C	0.106106	-2.389447	0.703949		
C	2.178147	2.056901	-0.132308		
C	-2.44565	1.80938	-0.018844		
C	2.523104	-0.704305	0.318552		
C	-2.480157	-0.962735	0.465166		
C	3.616869	0.075252	0.112008		
C	-3.653088	-0.284196	0.364128		
C	0.659385	-3.25283	-0.245374		
C	-0.372771	-2.899921	1.913461		
C	0.721164	-4.617604	0.011636		
C	-0.289061	-4.263025	2.172588		
C	0.252035	-5.122868	1.221036		
H	2.030527	3.063969	-0.49922		
H	-2.4199	2.873718	-0.218436		
H	2.651134	-1.753717	0.551378		
H	-2.491942	-2.033132	0.626415		
H	4.614097	-0.344937	0.161717		
H	-4.600041	-0.804009	0.448607		
H	-0.794624	-2.226122	2.651376		
H	1.137743	-5.286133	-0.732467		
H	-0.649164	-4.652495	3.117416		
H	0.308229	-6.186326	1.421979		
H	5.471719	1.842144	-0.33279		

H	4.450762	3.231694	-0.481497
H	-4.873895	2.763976	-0.143048
H	-5.708806	1.275528	0.132358
H	1.024265	-2.855776	-1.186486
O	2.398397	2.736028	1.817521
H	1.495772	3.070696	1.935197

RDA-C5'-TS-H

Atom	X	Y	Z		
O	-0.086507	1.803607	-0.214765	Zero-point Energy Correction	0.294612
N	4.596811	2.169487	-0.351697	Thermal Correction to Energy	0.313625
N	-4.8258	1.763701	0.079039	Thermal Correction to Enthalpy	0.314569
C	0.058008	-0.925826	0.443714	Thermal Correction to Free Energy	0.246883
C	1.208404	-0.173429	0.323238		
C	-1.203169	-0.265251	0.358509		
C	1.102705	1.263688	0.124081		
C	-1.237329	1.121055	0.050588		
C	3.4795	1.469205	-0.11211		
C	-3.661189	1.156346	0.148061		
C	0.125781	-2.391184	0.683175		
C	2.217288	2.032377	-0.216318		
C	-2.407634	1.821141	-0.056575		
C	2.536678	-0.704842	0.45489		
C	-2.453525	-0.931962	0.529525		
C	3.621826	0.075218	0.261633		
C	-3.63066	-0.258498	0.436715		
C	0.623163	-3.242322	-0.305196		
C	-0.294766	-2.920587	1.905564		
C	0.693904	-4.612775	-0.073879		
C	-0.213151	-4.289565	2.136439		
C	0.27811	-5.137436	1.146579		
H	2.084053	3.073191	-0.483018		
H	-2.388163	2.877911	-0.298524		
H	2.657304	-1.750283	0.70913		
H	-2.453486	-1.994306	0.736363		
H	4.623857	-0.325777	0.364364		
H	-4.577646	-0.766794	0.572334		
H	-0.677237	-2.25728	2.674077		
H	1.076059	-5.268736	-0.847122		
H	-0.533753	-4.693135	3.089623		
H	0.337001	-6.204396	1.326959		
H	5.502608	1.727934	-0.292696		
H	4.550213	3.142549	-0.617351		
H	-4.676085	2.753541	-0.132795		
H	0.949601	-2.828741	-1.253227		

Electronic Energy (EE) -992.795

O	0.845762	1.561696	2.237414
H	1.161793	2.471469	2.257255

RDA-C5-TS-H

Atom	X	Y	Z
O	-0.084983	1.828641	-0.117185
N	4.613577	2.220894	-0.390423
N	-4.835173	1.772593	0.120302
C	0.016073	-0.924163	0.411617
C	1.225141	-0.164478	0.216474
C	-1.197165	-0.286622	0.337491
C	1.122405	1.206602	-0.049431
C	-1.250167	1.135424	0.072411
C	3.5204	1.460155	-0.166312
C	-3.684437	1.176824	0.16634
C	0.106434	-2.379456	0.695771
C	2.231868	2.026283	-0.220879
C	-2.404989	1.835805	-0.007408
C	2.533584	-0.696197	0.312325
C	-2.479315	-0.946612	0.473138
C	3.648773	0.079351	0.12552
C	-3.639275	-0.267759	0.395677
C	0.644722	-3.250298	-0.255503
C	-0.343208	-2.887167	1.916859
C	0.719903	-4.613681	0.008772
C	-0.255105	-4.250011	2.183123
C	0.27292	-5.115256	1.22896
H	2.094794	3.072572	-0.466237
H	-2.377218	2.901215	-0.207356
H	2.656549	-1.746337	0.547675
H	-2.489334	-2.017896	0.631997
H	4.640443	-0.351159	0.199828
H	-4.588752	-0.781631	0.495003
H	-0.75688	-2.210672	2.65691
H	1.129027	-5.283867	-0.738087
H	-0.601411	-4.634506	3.135262
H	0.33688	-6.17722	1.435385
H	5.531193	1.842782	-0.208106
H	4.522658	3.222972	-0.46827
H	-4.692577	2.768856	-0.051341
H	0.99539	-2.857507	-1.20391
O	2.437251	2.569091	2.159014
H	1.505558	2.842589	2.104456

Electronic Energy (EE)	-992.794
Zero-point Energy Correction	0.294422
Thermal Correction to Energy	0.314017
Thermal Correction to Enthalpy	0.314962
Thermal Correction to Free Energy	0.245127

RDA-C6-TS-H

Atom	X	Y	Z		
O	-0.092491	1.833504	-0.080799	Electronic Energy (EE)	-992.789
N	4.597906	2.136172	-0.482576	Zero-point Energy Correction	0.292469
N	-4.840503	1.739229	0.122769	Thermal Correction to Energy	0.312114
C	0.018098	-0.932637	0.420612	Thermal Correction to Enthalpy	0.313058
C	1.219752	-0.169716	0.246386	Thermal Correction to Free Energy	0.243276
C	-1.199176	-0.292191	0.339478		
C	1.12065	1.21004	-0.013475		
C	-1.254024	1.133941	0.090087		
C	3.522661	1.438506	-0.1123		
C	-3.687121	1.152568	0.16922		
C	0.104186	-2.392397	0.687025		
C	2.223956	2.012544	-0.18584		
C	-2.412651	1.825111	0.010878		
C	2.529569	-0.721951	0.360846		
C	-2.473916	-0.965369	0.461347		
C	3.643033	0.040722	0.193417		
C	-3.638297	-0.293446	0.384273		
C	0.623364	-3.253307	-0.283128		
C	-0.332135	-2.912102	1.907529		
C	0.695392	-4.620297	-0.03684		
C	-0.249101	-4.27883	2.154713		
C	0.261513	-5.134629	1.182612		
H	2.105994	3.067813	-0.400208		
H	-2.393979	2.893203	-0.174746		
H	2.634594	-1.773774	0.593798		
H	-2.474963	-2.037674	0.610597		
H	4.635734	-0.386035	0.274946		
H	-4.585795	-0.812258	0.474012		
H	-0.732628	-2.242802	2.661323		
H	1.090937	-5.283019	-0.797516		
H	-0.585587	-4.67376	3.106065		
H	0.321774	-6.199499	1.374575		
H	5.519987	1.741764	-0.358121		
H	4.525726	3.130722	-0.647733		
H	-4.705733	2.739155	-0.037032		
H	0.963234	-2.849866	-1.231085		
O	3.286446	2.086804	2.172192		
H	2.509552	1.572568	2.429061		

RDA-C7-TS-H

Atom	X	Y	Z		
O	-0.10375	1.817466	-0.079613	Electronic Energy (EE)	-992.795
N	4.581044	2.177257	-0.354057	Zero-point Energy Correction	0.295513
				Thermal Correction to Energy	0.313953

N	-4.849602	1.754543	0.111747	Thermal Correction to Enthalpy	0.314897
C	-0.015758	-0.946817	0.429872	Thermal Correction to Free Energy	0.247984
C	1.203997	-0.184237	0.289827		
C	-1.222898	-0.308054	0.334245		
C	1.106217	1.212682	0.009403		
C	-1.270474	1.118905	0.090923		
C	3.486253	1.458981	-0.087873		
C	-3.704578	1.154528	0.158171		
C	0.071676	-2.407581	0.687411		
C	2.201492	2.015892	-0.196819		
C	-2.421671	1.819372	0.009781		
C	2.477922	-0.715793	0.42591		
C	-2.508295	-0.973519	0.437932		
C	3.619249	0.094556	0.348148		
C	-3.666464	-0.294888	0.359613		
C	0.613439	-3.2586	-0.279536		
C	-0.382767	-2.939409	1.895967		
C	0.687974	-4.627005	-0.04283		
C	-0.296273	-4.307696	2.134351		
C	0.235827	-5.153271	1.165013		
H	2.068386	3.060547	-0.45005		
H	-2.394534	2.887936	-0.172499		
H	2.604725	-1.76686	0.650603		
H	-2.516808	-2.047333	0.577612		
H	4.600223	-0.361867	0.309834		
H	-4.618024	-0.808031	0.438175		
H	-0.799478	-2.277966	2.647841		
H	1.100059	-5.281794	-0.801653		
H	-0.646813	-4.711676	3.076804		
H	0.298909	-6.219243	1.349911		
H	5.499028	1.777383	-0.221424		
H	4.51107	3.157656	-0.587215		
H	-4.704099	2.754305	-0.038034		
H	0.968183	-2.846323	-1.218095		
O	3.905597	0.630812	2.355544		
H	4.803715	0.276155	2.390393		

RDA-C8'-TS-H

Atom	X	Y	Z		
				Electronic Energy (EE)	-992.795
O	-0.105581	1.846104	-0.077543	Zero-point Energy Correction	0.294572
N	4.625835	2.16278	-0.297412	Thermal Correction to Energy	0.313769
N	-4.794131	1.796741	0.240216	Thermal Correction to Enthalpy	0.314713
C	0.071035	-0.916809	0.435283	Thermal Correction to Free Energy	0.246292
C	1.217943	-0.161117	0.31061		
C	-1.192648	-0.286659	0.22373		

C	1.121998	1.24333	-0.004762
C	-1.24246	1.138007	0.080362
C	3.540939	1.481545	-0.101849
C	-3.630292	1.148171	0.237465
C	0.131004	-2.376169	0.707406
C	2.203145	2.029395	-0.212785
C	-2.415326	1.84463	0.084312
C	2.555027	-0.690928	0.470768
C	-2.446638	-0.959079	0.387402
C	3.645889	0.073737	0.273742
C	-3.620045	-0.279474	0.386513
C	0.67002	-3.238964	-0.249636
C	-0.356962	-2.89545	1.908745
C	0.720025	-4.607931	-0.005835
C	-0.296873	-4.2636	2.15295
C	0.239327	-5.121403	1.195763
H	2.073893	3.079375	-0.450338
H	-2.404594	2.920444	-0.038985
H	2.661644	-1.730997	0.752407
H	-2.447224	-2.03614	0.49529
H	4.642847	-0.334332	0.392382
H	-4.567127	-0.794534	0.496336
H	-0.777328	-2.224976	2.650786
H	1.135177	-5.272157	-0.754682
H	-0.670084	-4.658877	3.090351
H	0.282055	-6.18744	1.386004
H	4.385235	3.124498	-0.545563
H	-4.829668	2.800669	0.128075
H	-5.664009	1.293607	0.348184
H	1.044046	-2.833623	-1.183761
O	-1.151924	-0.390624	-2.139049
H	-2.078499	-0.129432	-2.203478

RDA-C8-TS-H

Atom	X	Y	Z	Electronic Energy (EE)	-992.789
O	-0.107677	1.852189	0.011872	Zero-point Energy Correction	0.29477
N	4.562325	2.179458	-0.355712	Thermal Correction to Energy	0.313711
N	-4.856683	1.73792	0.076195	Thermal Correction to Enthalpy	0.314655
C	0.044482	-0.922301	0.437759	Thermal Correction to Free Energy	0.247114
C	1.193849	-0.171014	0.326644		
C	-1.224299	-0.271871	0.326697		
C	1.105882	1.246078	0.077667		
C	-1.261731	1.133421	0.131101		
C	3.497218	1.413326	-0.09703		
C	-3.686124	1.139463	0.141681		

C	0.105314	-2.38364	0.697467
C	2.202067	2.023342	-0.109092
C	-2.434689	1.831428	0.045677
C	2.53278	-0.71374	0.521294
C	-2.467935	-0.962102	0.397404
C	3.637909	0.039498	0.165758
C	-3.650247	-0.293278	0.311158
C	0.619404	-3.250508	-0.268316
C	-0.353347	-2.894109	1.914173
C	0.672972	-4.618355	-0.018396
C	-0.287165	-4.260249	2.164351
C	0.222889	-5.124025	1.197894
H	2.098959	3.084443	-0.299727
H	-2.418614	2.905937	-0.098368
H	2.629769	-1.7864	0.58098
H	-2.461311	-2.037621	0.518352
H	4.628421	-0.398058	0.171679
H	-4.595286	-0.819891	0.365205
H	-0.751348	-2.217976	2.663296
H	1.067829	-5.287151	-0.774039
H	-0.635446	-4.649649	3.113651
H	0.26886	-6.189053	1.392858
H	5.491473	1.783511	-0.353119
H	4.455934	3.160815	-0.568522
H	-4.712878	2.743652	-0.04643
H	0.971942	-2.851099	-1.213111
O	2.359024	-0.694241	2.622436
H	3.28241	-0.47405	2.786142

RDA-C9-TS-H

Atom	X	Y	Z		
O	-0.111503	1.823692	-0.046834	Electronic Energy (EE)	-992.798
N	4.607593	2.143085	-0.456133	Zero-point Energy Correction	0.294572
N	-4.815265	1.727297	-0.085205	Thermal Correction to Energy	0.313475
C	0.045872	-0.889943	0.696304	Thermal Correction to Enthalpy	0.314419
C	1.23438	-0.138264	0.3888	Thermal Correction to Free Energy	0.247281
C	-1.197237	-0.274266	0.45835		
C	1.108215	1.219267	0.025018		
C	-1.247678	1.106224	0.122579		
C	3.512036	1.453532	-0.187395		
C	-3.64458	1.09898	0.082235		
C	0.122311	-2.368315	0.853795		
C	2.198447	2.006699	-0.262343		
C	-2.428139	1.78787	-0.057877		
C	2.546854	-0.680262	0.490306		

C	-2.44861	-0.949429	0.568638
C	3.643581	0.074635	0.210316
C	-3.628313	-0.297339	0.390718
C	0.658949	-3.117285	-0.1979
C	-0.393402	-3.024262	1.972538
C	0.683046	-4.506562	-0.126042
C	-0.359717	-4.412357	2.043075
C	0.177923	-5.156494	0.995801
H	2.058815	3.044518	-0.543202
H	-2.411254	2.839843	-0.315
H	2.661859	-1.708021	0.809443
H	-2.454999	-2.007903	0.794636
H	4.642307	-0.337096	0.2895
H	-4.572816	-0.821827	0.473818
H	-0.802253	-2.436764	2.783693
H	1.098866	-5.077941	-0.947427
H	-0.753613	-4.912717	2.91992
H	0.202927	-6.238351	1.05444
H	4.34846	3.098571	-0.716249
H	-4.847441	2.71035	-0.313249
H	-5.685853	1.224816	0.008226
H	1.048715	-2.614267	-1.076417
O	0.346201	-0.386489	2.981471
H	0.567917	0.533469	2.800407

RDA-C9-TS-M

Atom	X	Y	Z		
O	-0.10653	1.81551	-0.000788	Electronic Energy (EE)	-993.27
N	4.557623	2.164254	-0.451819	Zero-point Energy Correction	0.309072
N	-4.817829	1.739568	-0.092221	Thermal Correction to Energy	0.328008
C	0.048939	-0.90288	0.706266	Thermal Correction to Enthalpy	0.328952
C	1.233663	-0.144454	0.451396	Thermal Correction to Free Energy	0.262184
C	-1.208838	-0.281222	0.47426		
C	1.108174	1.22997	0.069875		
C	-1.251759	1.093335	0.150395		
C	3.483049	1.442223	-0.160484		
C	-3.648745	1.098428	0.083316		
C	0.123307	-2.381822	0.856048		
C	2.190908	2.010799	-0.224986		
C	-2.42723	1.7828	-0.039531		
C	2.556331	-0.688115	0.52131		
C	-2.454794	-0.95178	0.571419		
C	3.640667	0.066431	0.227366		
C	-3.636546	-0.293524	0.384089		
C	0.631876	-3.130251	-0.208389		

C	-0.362704	-3.030355	1.991737
C	0.655227	-4.519118	-0.133802
C	-0.328147	-4.418628	2.06432
C	0.178937	-5.164688	1.003333
H	2.055608	3.046104	-0.513903
H	-2.399829	2.836942	-0.288772
H	2.67875	-1.716999	0.835086
H	-2.467188	-2.011868	0.792848
H	4.642591	-0.342499	0.28542
H	-4.580788	-0.820447	0.458358
H	-0.75527	-2.44534	2.814421
H	1.048161	-5.094084	-0.964376
H	-0.698722	-4.917563	2.952422
H	0.2029	-6.246855	1.063259
H	5.482888	1.756304	-0.40621
H	4.477802	3.13679	-0.721179
H	-4.842752	2.722229	-0.321002
H	-5.6939	1.244232	-0.017306
H	1.000251	-2.626087	-1.096111
O	0.634196	-0.328853	2.643726
H	0.244298	0.559655	2.712412

RDA-C9-TS-P

Atom	X	Y	Z		
O	-0.160493	-2.333437	-0.038641	Zero-point Energy Correction	0.30919
N	-4.85224	-2.156321	-0.280056	Thermal Correction to Energy	0.328171
N	4.514898	-2.881972	-0.306867	Thermal Correction to Enthalpy	0.329115
C	0.051181	0.466132	0.156388	Thermal Correction to Free Energy	0.262359
C	-1.225455	-0.168908	0.069196		
C	1.212505	-0.347533	0.013579		
C	-1.287189	-1.593182	-0.041446		
C	1.070138	-1.750259	-0.056904		
C	-3.680092	-1.536635	-0.160098		
C	3.441751	-2.075078	-0.203906		
C	0.165233	1.943665	0.004279		
C	-2.474357	-2.268774	-0.147041		
C	2.142639	-2.607788	-0.158392		
C	-2.465356	0.54449	0.069266		
C	2.532577	0.164431	-0.048034		
C	-3.647685	-0.105375	-0.043937		
C	3.61393	-0.663028	-0.152899		
C	-0.240635	2.510518	-1.206116		
C	0.722461	2.748389	0.997525		
C	-0.087824	3.8761	-1.420284		
C	0.865786	4.114294	0.779734		

Note: the header row of the second table also contains "Electronic Energy (EE)" with value -993.253 to the right of the X/Y/Z headers.

C	0.463693	4.679551	-0.427215
H	-2.479788	-3.348324	-0.233075
H	1.974493	-3.676568	-0.213319
H	-2.441165	1.620877	0.181673
H	2.681003	1.236944	-0.019759
H	-4.586241	0.435828	-0.037037
H	4.616845	-0.25571	-0.204312
H	1.023601	2.302434	1.936865
H	-0.401449	4.309761	-2.362509
H	1.290701	4.738471	1.557092
H	0.578206	5.744491	-0.592731
H	-5.716546	-1.632322	-0.292074
H	-4.909945	-3.162714	-0.355513
H	4.410685	-3.883039	-0.368484
H	5.446254	-2.499412	-0.36193
H	-0.667899	1.88142	-1.980447
O	-0.498121	0.382019	2.15243
H	-0.228259	-0.518805	2.401534

RDA-H2A-C1'-TS-H

Atom	X	Y	Z		
O	-0.127739	1.851511	-0.176182	Electronic Energy (EE)	-993.69
N	4.621737	2.277713	-0.314716	Zero-point Energy Correction	0.323889
N	-4.774482	1.8871	0.409618	Thermal Correction to Energy	0.343042
C	-0.013717	-0.909205	0.355425	Thermal Correction to Enthalpy	0.343986
C	1.184804	-0.13191	0.233058	Thermal Correction to Free Energy	0.27611
C	-1.245889	-0.276395	0.094067		
C	1.074891	1.232738	-0.079311		
C	-1.273683	1.179687	0.029442		
C	3.424529	1.459695	-0.115571		
C	-3.64424	1.220038	0.317835		
C	0.052588	-2.35418	0.64821		
C	2.189451	2.042857	-0.26987		
C	-2.418435	1.90275	0.114504		
C	2.48134	-0.664717	0.411469		
C	-2.529157	-0.928872	0.320632		
C	3.597022	0.119714	0.23484		
C	-3.666755	-0.222204	0.419223		
C	0.711765	-3.211403	-0.239827		
C	-0.541453	-2.868155	1.804755		
C	0.763432	-4.574282	0.025422		
C	-0.464715	-4.229557	2.074451		
C	0.181809	-5.082928	1.184234		
H	2.068218	3.091007	-0.515586		
H	-2.394184	2.982289	0.035152		

H	2.597765	-1.702409	0.69327
H	-2.549888	-2.009754	0.372948
H	4.593049	-0.284256	0.368407
H	-4.623387	-0.709261	0.566537
H	-1.045129	-2.198767	2.493466
H	1.260432	-5.237992	-0.671807
H	-0.913787	-4.623277	2.978242
H	0.232164	-6.145011	1.393164
H	5.144213	1.966096	-1.143386
H	4.391715	3.269452	-0.442897
H	-4.79262	2.896736	0.330157
H	-5.651488	1.401229	0.553435
H	1.164465	-2.807732	-1.139084
H	5.255482	2.202513	0.490758
O	-1.14635	-0.483367	-1.886398
H	-1.17303	-1.453545	-1.938274

RDA-H2A-C1-TS-H

Atom	X	Y	Z		
O	-0.117232	1.801249	-0.038273	Electronic Energy (EE)	-993.692
N	4.552883	2.192409	-0.274855	Zero-point Energy Correction	0.323508
N	-4.887354	1.839743	0.046684	Thermal Correction to Energy	0.342884
C	0.039331	-0.952117	0.437364	Thermal Correction to Enthalpy	0.343828
C	1.181101	-0.203134	0.277323	Thermal Correction to Free Energy	0.275052
C	-1.241667	-0.291201	0.34362		
C	1.087201	1.20789	0.027903		
C	-1.260073	1.089611	0.114039		
C	3.466333	1.455577	-0.092396		
C	-3.619979	1.112884	0.146096		
C	0.099307	-2.405171	0.71221		
C	2.178522	2.018373	-0.152254		
C	-2.445772	1.812702	0.015503		
C	2.519401	-0.737937	0.434939		
C	-2.478628	-0.959343	0.448805		
C	3.619233	0.048153	0.170532		
C	-3.664364	-0.266161	0.35655		
C	0.661795	-3.273971	-0.226217		
C	-0.408791	-2.907383	1.913688		
C	0.710363	-4.638567	0.036078		
C	-0.338463	-4.270143	2.177529		
C	0.215519	-5.136558	1.238226		
H	2.044499	3.076987	-0.335304		
H	-2.420697	2.881202	-0.162315		
H	2.645836	-1.806013	0.537189		
H	-2.496524	-2.030862	0.59714		

H	4.618321	-0.369753	0.207884
H	-4.617101	-0.774846	0.43759
H	-0.84374	-2.229025	2.639605
H	1.137027	-5.311064	-0.698412
H	-0.720443	-4.655086	3.11538
H	0.260474	-6.199673	1.443166
H	5.473327	1.775435	-0.2253
H	4.480442	3.182203	-0.472358
H	-4.736592	2.848865	-0.059474
H	-5.435239	1.515581	-0.760081
H	1.047312	-2.879147	-1.159799
O	2.749927	-0.381762	2.454911
H	3.634377	-0.759154	2.599347
H	-5.461814	1.6917	0.885791

RDA-H2A-C11-TS-H

Atom	X	Y	Z		
O	-0.163192	1.708127	-0.248466	Electronic Energy (EE)	-993.682
N	4.585219	2.06007	-0.306829	Zero-point Energy Correction	0.323791
N	-4.816298	1.974857	0.226326	Thermal Correction to Energy	0.342776
C	-0.149428	-1.028992	0.337433	Thermal Correction to Enthalpy	0.34372
C	1.090182	-0.291781	0.208006	Thermal Correction to Free Energy	0.276711
C	-1.338394	-0.323882	0.337003		
C	1.01942	1.071437	-0.114358		
C	-1.322511	1.08654	0.040446		
C	3.373237	1.258857	-0.127274		
C	-3.696621	1.264908	0.259762		
C	-0.089492	-2.514075	0.513052		
C	2.153007	1.857231	-0.313019		
C	-2.449748	1.85887	-0.013264		
C	2.375941	-0.833966	0.433796		
C	-2.638709	-0.891151	0.605626		
C	3.509201	-0.072141	0.268289		
C	-3.757587	-0.136453	0.583664		
C	0.87767	-3.277098	-0.225394		
C	-0.536698	-3.04368	1.765272		
C	1.216716	-4.553152	0.174106		
C	-0.171964	-4.312758	2.152413		
C	0.690337	-5.079378	1.355506		
H	2.050267	2.899744	-0.589329		
H	-2.375803	2.909622	-0.263555		
H	2.483139	-1.856716	0.767127		
H	-2.712924	-1.941002	0.839513		
H	4.492786	-0.49116	0.442716		
H	-4.725739	-0.570998	0.803174		

H	-1.157016	-2.43217	2.408858
H	1.901055	-5.138938	-0.426474
H	-0.536555	-4.712269	3.090774
H	0.967655	-6.077716	1.671774
H	5.330598	1.509274	-0.749344
H	4.408718	2.884632	-0.892067
H	-4.80057	2.958995	-0.006715
H	-5.708365	1.538476	0.416811
H	1.280071	-2.855916	-1.138693
O	-1.465646	-2.988884	-0.784998
H	-1.370608	-3.954572	-0.784796
H	4.945548	2.392088	0.597565

RDA-H2A-C12-TS-H

Atom	X	Y	Z	Electronic Energy (EE)	-993.692
O	-0.108499	1.80876	-0.078273	Zero-point Energy Correction	0.323027
N	4.637966	2.259968	-0.275738	Thermal Correction to Energy	0.34262
N	-4.800522	1.771671	0.077783	Thermal Correction to Enthalpy	0.343564
C	0.009651	-0.939723	0.426015	Thermal Correction to Free Energy	0.274303
C	1.216396	-0.172484	0.249293		
C	-1.20871	-0.298533	0.346905		
C	1.096761	1.200556	-0.006313		
C	-1.252283	1.116238	0.091968		
C	3.444344	1.43276	-0.090508		
C	-3.641577	1.131907	0.152097		
C	0.095443	-2.389481	0.702058		
C	2.208428	2.021783	-0.186898		
C	-2.420943	1.820269	0.001046		
C	2.514169	-0.717189	0.355644		
C	-2.476469	-0.970646	0.464455		
C	3.624793	0.075167	0.185017		
C	-3.639482	-0.291475	0.376867		
C	0.657353	-3.25746	-0.216825		
C	-0.351319	-2.878746	1.960805		
C	0.715483	-4.627226	0.062102		
C	-0.331893	-4.271893	2.198897		
C	0.21783	-5.131429	1.264073		
H	2.081749	3.079012	-0.386757		
H	-2.40381	2.885739	-0.190739		
H	2.636038	-1.767285	0.584817		
H	-2.488507	-2.04293	0.613681		
H	4.623746	-0.334969	0.267963		
H	-4.592995	-0.799157	0.460669		
H	-0.94212	-2.234023	2.599411		
H	1.142651	-5.300211	-0.671473		

H	-0.72919	-4.649108	3.133571
H	0.253112	-6.196102	1.459389
H	5.17395	1.952262	-1.096905
H	4.402143	3.249908	-0.406584
H	-4.834276	2.768198	-0.092324
H	-5.673627	1.271686	0.179831
H	1.031097	-2.878174	-1.161221
O	1.3349	-2.368432	3.004645
H	1.068912	-2.799398	3.832934
H	5.261668	2.190166	0.538191

RDA-H2A-C13-TS-H

Atom	X	Y	Z		
O	-0.114269	1.816689	-0.118075	Electronic Energy (EE)	-993.69
N	4.634242	2.257295	-0.316051	Zero-point Energy Correction	0.323154
N	-4.805211	1.792528	0.043977	Thermal Correction to Energy	0.342611
C	0.000841	-0.920115	0.450471	Thermal Correction to Enthalpy	0.343555
C	1.207818	-0.155584	0.263421	Thermal Correction to Free Energy	0.274586
C	-1.217066	-0.281954	0.349379		
C	1.090466	1.210159	-0.027958		
C	-1.258549	1.128497	0.067541		
C	3.438702	1.437839	-0.110113		
C	-3.648007	1.150771	0.128861		
C	0.092644	-2.367387	0.75172		
C	2.203615	2.025366	-0.226143		
C	-2.425625	1.833221	-0.035167		
C	2.504517	-0.697708	0.391627		
C	-2.487051	-0.94862	0.477061		
C	3.616868	0.088433	0.203786		
C	-3.64856	-0.268333	0.377262		
C	0.660541	-3.242528	-0.19347		
C	-0.381126	-2.858741	1.953334		
C	0.71551	-4.610638	0.057702		
C	-0.234171	-4.234628	2.259891		
C	0.250007	-5.113243	1.261202		
H	2.078677	3.077206	-0.453524		
H	-2.405931	2.895054	-0.246019		
H	2.626293	-1.740816	0.652727		
H	-2.500571	-2.017352	0.646305		
H	4.615105	-0.319829	0.303336		
H	-4.603027	-0.772821	0.469019		
H	-0.820396	-2.190047	2.684819		
H	1.115762	-5.277981	-0.695291		
H	-0.75891	-4.638436	3.115321		
H	0.283642	-6.177113	1.464171		

H	5.160114	1.937698	-1.139287
H	4.401826	3.246857	-0.455528
H	-4.836057	2.786026	-0.143388
H	-5.679649	1.297007	0.155471
H	1.029424	-2.845332	-1.132756
O	1.482781	-4.079246	3.342716
H	1.660076	-5.03231	3.388025
H	5.265744	2.193102	0.492207

RDA-H2A-C14-TS-H

Atom	X	Y	Z		
O	-0.10789	1.792889	-0.101865	Electronic Energy (EE)	-993.69
N	4.642373	2.211916	-0.284003	Zero-point Energy Correction	0.323094
N	-4.799597	1.792818	0.032857	Thermal Correction to Energy	0.342626
C	-0.009395	-0.94457	0.465263	Thermal Correction to Enthalpy	0.34357
C	1.203186	-0.186867	0.281966	Thermal Correction to Free Energy	0.274337
C	-1.223966	-0.29955	0.360098		
C	1.093273	1.179962	-0.007877		
C	-1.256649	1.110772	0.077252		
C	3.442808	1.397397	-0.081932		
C	-3.646319	1.145092	0.123749		
C	0.071135	-2.390426	0.766988		
C	2.210741	1.990278	-0.201582		
C	-2.419501	1.821353	-0.032228		
C	2.497228	-0.734521	0.414876		
C	-2.497962	-0.960335	0.478111		
C	3.6136	0.046995	0.231739		
C	-3.655428	-0.27446	0.370648		
C	0.648772	-3.263699	-0.163885		
C	-0.444363	-2.880133	1.980226		
C	0.719823	-4.614908	0.115255		
C	-0.35402	-4.222561	2.273018		
C	0.288498	-5.107312	1.371052		
H	2.090583	3.042694	-0.428764		
H	-2.392901	2.883094	-0.24274		
H	2.613764	-1.778472	0.674509		
H	-2.519245	-2.02941	0.645003		
H	4.609582	-0.365926	0.334651		
H	-4.612884	-0.774537	0.455209		
H	-0.906545	-2.192876	2.679628		
H	1.140164	-5.301097	-0.610231		
H	-0.742933	-4.605646	3.208316		
H	0.190217	-6.174793	1.51658		
H	5.16714	1.893071	-1.108254		
H	4.414454	3.203029	-0.419856		

H	-4.82411	2.78682	-0.15295
H	-5.677372	1.301624	0.137408
H	1.014647	-2.877027	-1.108239
O	2.123739	-5.126923	2.274599
H	2.594019	-5.548997	1.537927
H	5.27322	2.141761	0.524268

RDA-H2A-C2-TS-H

Atom	X	Y	Z	Electronic Energy (EE)	-993.695
O	-0.092202	1.80695	-0.072277	Zero-point Energy Correction	0.323343
N	4.568096	2.199191	-0.406874	Thermal Correction to Energy	0.342833
N	-4.855929	1.834102	0.107205	Thermal Correction to Enthalpy	0.343777
C	0.079522	-0.94184	0.447249	Thermal Correction to Free Energy	0.274536
C	1.227019	-0.188222	0.258433		
C	-1.19931	-0.283108	0.377194		
C	1.117968	1.221669	-0.022585		
C	-1.22629	1.094994	0.120928		
C	3.488464	1.477866	-0.171329		
C	-3.585458	1.112823	0.198054		
C	0.148215	-2.395001	0.708644		
C	2.198022	2.030902	-0.247367		
C	-2.416264	1.812584	0.03164		
C	2.548783	-0.720254	0.353845		
C	-2.435517	-0.951939	0.513933		
C	3.651549	0.087898	0.22025		
C	-3.623153	-0.263199	0.432999		
C	0.730681	-3.247893	-0.233428		
C	-0.370548	-2.917628	1.897346		
C	0.786142	-4.615088	0.011673		
C	-0.29096	-4.28248	2.145103		
C	0.281207	-5.132282	1.201413		
H	2.056083	3.078863	-0.479058		
H	-2.398283	2.877479	-0.167382		
H	2.686799	-1.767442	0.587709		
H	-2.450254	-2.021187	0.676305		
H	4.645887	-0.338179	0.183181		
H	-4.573077	-0.772395	0.539204		
H	-0.818319	-2.252702	2.627678		
H	1.226599	-5.274661	-0.726308		
H	-0.679791	-4.68219	3.073868		
H	0.332614	-6.197484	1.393531		
H	5.489648	1.78725	-0.33287		
H	4.495312	3.178255	-0.653517		
H	-4.709723	2.843516	-0.003385		
H	-5.408597	1.505167	-0.6946		

H	1.123269	-2.83888	-1.15781
O	3.835148	0.58812	2.264503
H	3.937126	-0.32193	2.589744
H	-5.4234	1.685821	0.951087

RDA-H2A-C3-TS-H

Atom	X	Y	Z
O	-0.095309	1.802448	0.013649
N	4.553554	2.100166	-0.5636
N	-4.863323	1.825334	0.064822
C	0.071309	-0.95355	0.471831
C	1.219111	-0.197712	0.311719
C	-1.207123	-0.293445	0.389371
C	1.10095	1.20339	0.077188
C	-1.234556	1.089628	0.158011
C	3.524608	1.468663	-0.006454
C	-3.592646	1.107637	0.175298
C	0.132706	-2.411874	0.714814
C	2.200672	2.034329	-0.076062
C	-2.422165	1.810739	0.049917
C	2.56837	-0.736887	0.408832
C	-2.446089	-0.963771	0.489364
C	3.653377	0.035482	0.260103
C	-3.630789	-0.273329	0.389783
C	0.714084	-3.255697	-0.235262
C	-0.39296	-2.946934	1.894399
C	0.761885	-4.6263	-0.00744
C	-0.323744	-4.315787	2.124649
C	0.24831	-5.156454	1.17306
H	2.066658	3.077932	-0.330276
H	-2.397067	2.878705	-0.131147
H	2.683794	-1.79268	0.614023
H	-2.464154	-2.035141	0.636532
H	4.655722	-0.370085	0.328621
H	-4.582435	-0.785091	0.465329
H	-0.8413	-2.289082	2.630941
H	1.202865	-5.278626	-0.751566
H	-0.720241	-4.725254	3.045938
H	0.292994	-6.224335	1.351537
H	5.481033	1.7083	-0.466566
H	4.478785	3.084632	-0.784872
H	-4.718726	2.833705	-0.056797
H	-5.408856	1.485242	-0.737197
H	1.114801	-2.836985	-1.151906
O	3.273431	2.235415	1.736157

Electronic Energy (EE)	-993.686
Zero-point Energy Correction	0.324195
Thermal Correction to Energy	0.343181
Thermal Correction to Enthalpy	0.344126
Thermal Correction to Free Energy	0.276532

H	2.814929	1.575428	2.280849
H	-5.437438	1.686532	0.905981

RDA-H2A-C4'-TS-H

Atom	X	Y	Z
O	-0.099322	1.765388	-0.16157
N	4.572813	2.168264	-0.311893
N	-4.868394	1.829422	0.077483
C	0.067543	-0.959884	0.473856
C	1.205743	-0.211357	0.316795
C	-1.216451	-0.292978	0.403187
C	1.08852	1.21531	0.104252
C	-1.243109	1.070434	0.089458
C	3.500941	1.433587	-0.08885
C	-3.600406	1.104241	0.183403
C	0.131454	-2.421895	0.703613
C	2.21056	2.012789	-0.175123
C	-2.427663	1.788422	-0.029751
C	2.546175	-0.731584	0.436706
C	-2.451309	-0.947786	0.581022
C	3.637467	0.040142	0.248134
C	-3.639115	-0.256667	0.482961
C	0.68055	-3.257766	-0.271505
C	-0.348443	-2.965392	1.898476
C	0.740942	-4.630175	-0.054362
C	-0.268948	-4.335799	2.116354
C	0.270243	-5.169442	1.139361
H	2.076787	3.053095	-0.445994
H	-2.406248	2.843342	-0.276013
H	2.669537	-1.77648	0.691801
H	-2.470171	-2.008239	0.793298
H	4.636974	-0.366049	0.345231
H	-4.589022	-0.756479	0.628237
H	-0.768641	-2.31341	2.656489
H	1.158858	-5.275999	-0.817312
H	-0.630315	-4.752089	3.049033
H	0.323494	-6.238332	1.309292
H	5.497688	1.756977	-0.263391
H	4.4931	3.151659	-0.543273
H	-4.717429	2.836462	-0.047246
H	-5.419774	1.491957	-0.721418
H	1.04861	-2.831177	-1.19812
O	1.450223	1.885935	1.969289
H	1.135336	2.802561	1.899
H	-5.43918	1.696609	0.92139

Electronic Energy (EE)	-993.692
Zero-point Energy Correction	0.324164
Thermal Correction to Energy	0.343138
Thermal Correction to Enthalpy	0.344082
Thermal Correction to Free Energy	0.276718

RDA-H2A-C4-TS-H

Atom	X	Y	Z		
O	-0.080703	1.807793	-0.044331	Zero-point Energy Correction	0.323627
N	4.57374	2.184347	-0.416244	Thermal Correction to Energy	0.343046
N	-4.846631	1.848579	0.081688	Thermal Correction to Enthalpy	0.343991
C	0.08149	-0.938441	0.460177	Thermal Correction to Free Energy	0.275196
C	1.229229	-0.1865	0.271741		
C	-1.195641	-0.276971	0.387735		
C	1.114606	1.212989	0.013565		
C	-1.21996	1.10162	0.134756		
C	3.509366	1.450181	-0.16295		
C	-3.577579	1.126868	0.185768		
C	0.14569	-2.391917	0.720453		
C	2.216277	2.042304	-0.135927		
C	-2.40662	1.824351	0.033066		
C	2.568223	-0.719511	0.354871		
C	-2.435351	-0.943211	0.51134		
C	3.655014	0.052217	0.14146		
C	-3.619019	-0.250765	0.418655		
C	0.735197	-3.244992	-0.216971		
C	-0.38485	-2.914056	1.90413		
C	0.78515	-4.61244	0.027463		
C	-0.310376	-4.279235	2.151587		
C	0.268416	-5.129364	1.212325		
H	2.077896	3.074199	-0.432033		
H	-2.382289	2.889341	-0.16475		
H	2.694144	-1.764891	0.603879		
H	-2.454789	-2.012804	0.670559		
H	4.655591	-0.359293	0.196845		
H	-4.571347	-0.757929	0.512996		
H	-0.837618	-2.248661	2.630952		
H	1.230895	-5.272478	-0.706889		
H	-0.708401	-4.67886	3.076466		
H	0.315855	-6.194815	1.404068		
H	5.498514	1.771829	-0.404713		
H	4.48845	3.173152	-0.617867		
H	-4.699441	2.857444	-0.032906		
H	-5.393388	1.515498	-0.722553		
H	1.137208	-2.836215	-1.137394		
O	2.376407	2.530542	1.999776		
H	1.585245	3.092379	1.966539		
H	-5.420559	1.704711	0.92207		

Electronic Energy (EE): -993.698

RDA-H2A-C5'-TS-H

Atom	X	Y	Z		
				Electronic Energy (EE)	-993.688
O	-0.108273	1.792373	-0.171462	Zero-point Energy Correction	0.323156
N	4.643153	2.245985	-0.313557	Thermal Correction to Energy	0.342454
N	-4.793357	1.771989	0.059175	Thermal Correction to Enthalpy	0.343398
C	0.006831	-0.950774	0.450662	Thermal Correction to Free Energy	0.275134
C	1.209232	-0.184149	0.289125		
C	-1.208213	-0.297426	0.37344		
C	1.083092	1.227334	0.117547		
C	-1.252229	1.1104	0.066214		
C	3.445435	1.438751	-0.081548		
C	-3.641962	1.128766	0.143645		
C	0.093923	-2.406907	0.708776		
C	2.217504	2.032538	-0.155945		
C	-2.416479	1.808853	-0.048176		
C	2.501092	-0.718586	0.387384		
C	-2.478676	-0.961096	0.523423		
C	3.613534	0.078541	0.205116		
C	-3.641892	-0.285185	0.422357		
C	0.64882	-3.254083	-0.254264		
C	-0.373375	-2.93331	1.915414		
C	0.720509	-4.621333	-0.014153		
C	-0.282465	-4.299834	2.156018		
C	0.259226	-5.144633	1.191228		
H	2.084036	3.083689	-0.385269		
H	-2.401325	2.865484	-0.284293		
H	2.631931	-1.77093	0.60091		
H	-2.489695	-2.025925	0.71465		
H	4.610901	-0.339051	0.273598		
H	-4.595553	-0.786711	0.535388		
H	-0.795963	-2.270584	2.662597		
H	1.139029	-5.276947	-0.76835		
H	-0.638018	-4.703139	3.096583		
H	0.32261	-6.210074	1.378441		
H	5.188859	1.875476	-1.101793		
H	4.410694	3.223665	-0.522627		
H	-4.822247	2.762585	-0.147584		
H	-5.669476	1.283725	0.19467		
H	1.009232	-2.841939	-1.190425		
O	1.081775	1.603752	2.120513		
H	1.309486	2.548923	2.103089		
H	5.254944	2.239139	0.512802		

RDA-H2A-C5-TS-H

Atom	X	Y	Z	Electronic Energy (EE)	-993.699

O	-0.147563	1.809068	-0.156986	Zero-point Energy Correction	0.322415
N	4.587636	2.302827	-0.347642	Thermal Correction to Energy	0.342259
N	-4.835689	1.762426	0.061359	Thermal Correction to Enthalpy	0.343204
C	-0.01104	-0.933823	0.400805	Thermal Correction to Free Energy	0.273014
C	1.1896	-0.160224	0.195384		
C	-1.232962	-0.296669	0.319999		
C	1.060739	1.207513	-0.08657		
C	-1.285605	1.113593	0.042313		
C	3.408954	1.44936	-0.194645		
C	-3.674334	1.125724	0.131989		
C	0.085996	-2.379627	0.699444		
C	2.165551	2.030382	-0.295522		
C	-2.457012	1.813817	-0.04383		
C	2.490792	-0.694622	0.296681		
C	-2.498783	-0.968606	0.459593		
C	3.597657	0.099264	0.099935		
C	-3.665019	-0.293591	0.375662		
C	0.670683	-3.248298	-0.22717		
C	-0.41043	-2.878659	1.906624		
C	0.742527	-4.608228	0.049457		
C	-0.31563	-4.237369	2.185813		
C	0.254858	-5.102949	1.256761		
H	2.031742	3.08295	-0.515269		
H	-2.444326	2.876121	-0.252893		
H	2.621935	-1.739366	0.54444		
H	-2.506117	-2.038139	0.623181		
H	4.598978	-0.305045	0.183339		
H	-4.615717	-0.803632	0.475479		
H	-0.856709	-2.199902	2.624982		
H	1.182265	-5.280844	-0.677131		
H	-0.691493	-4.618645	3.127634		
H	0.320207	-6.162732	1.473193		
H	5.411447	1.756882	-0.624466		
H	4.436099	3.03266	-1.054161		
H	-4.873392	2.756111	-0.123388		
H	-5.706313	1.26218	0.180833		
H	1.051905	-2.85828	-1.164554		
O	3.092727	2.611669	2.299359		
H	2.140122	2.578434	2.507758		
H	4.8075	2.77341	0.540814		

RDA-H2A-C6-TS-H

Atom	X	Y	Z	Electronic Energy (EE)	-993.69
O	-0.139049	1.80327	-0.059822	Zero-point Energy Correction	0.323126
N	4.596458	2.206838	-0.474994	Thermal Correction to Energy	0.342419

N	-4.828017	1.749226	0.081946	Thermal Correction to Enthalpy	0.343363
C	-0.012459	-0.954921	0.437383	Thermal Correction to Free Energy	0.275057
C	1.189684	-0.181451	0.264944		
C	-1.232127	-0.312374	0.354346		
C	1.069253	1.196294	0.0131		
C	-1.279962	1.105602	0.104034		
C	3.435921	1.440608	-0.022833		
C	-3.669348	1.1132	0.157423		
C	0.075797	-2.409161	0.701472		
C	2.171114	2.012252	-0.178591		
C	-2.449057	1.805891	0.012614		
C	2.492606	-0.732892	0.35604		
C	-2.49951	-0.987831	0.463764		
C	3.600705	0.048922	0.185454		
C	-3.664047	-0.311921	0.375248		
C	0.651315	-3.258297	-0.248147		
C	-0.421688	-2.934269	1.896659		
C	0.714781	-4.6252	-0.005508		
C	-0.337162	-4.300466	2.141206		
C	0.225599	-5.146584	1.189866		
H	2.04922	3.066228	-0.398597		
H	-2.434834	2.872139	-0.175037		
H	2.611274	-1.787251	0.564907		
H	-2.507869	-2.060086	0.607699		
H	4.600808	-0.361983	0.250739		
H	-4.616548	-0.822724	0.451419		
H	-0.862713	-2.270615	2.632233		
H	1.148406	-5.282376	-0.749704		
H	-0.715255	-4.702609	3.073394		
H	0.283386	-6.21193	1.379393		
H	5.442211	1.899166	0.01951		
H	4.469577	3.208352	-0.286536		
H	-4.864224	2.746614	-0.084857		
H	-5.700521	1.246625	0.180139		
H	1.03302	-2.847926	-1.176663		
O	3.800053	2.097782	1.869088		
H	3.140127	1.593772	2.375812		
H	4.746439	2.08235	-1.483957		

RDA-H2A-C7-TS-H

Atom	X	Y	Z	Electronic Energy (EE)	-993.691
O	-0.117001	1.799161	-0.108319	Zero-point Energy Correction	0.323412
N	4.618703	2.286596	-0.287435	Thermal Correction to Energy	0.342755
N	-4.806038	1.779629	0.097286	Thermal Correction to Enthalpy	0.343699
C	-0.001286	-0.947816	0.426632	Thermal Correction to Free Energy	0.275226

C	1.203455	-0.173943	0.237191
C	-1.219277	-0.305192	0.346335
C	1.079276	1.196771	-0.040206
C	-1.264726	1.107351	0.082218
C	3.422973	1.46535	-0.152347
C	-3.651639	1.13343	0.164552
C	0.092654	-2.39599	0.711602
C	2.191842	2.031712	-0.248692
C	-2.427505	1.816802	-0.002146
C	2.497338	-0.709215	0.337349
C	-2.489424	-0.970954	0.474876
C	3.624805	0.106895	0.204428
C	-3.651379	-0.287913	0.393908
C	0.686391	-3.255147	-0.218302
C	-0.414669	-2.90673	1.90934
C	0.756822	-4.617524	0.045995
C	-0.321357	-4.267966	2.176075
C	0.258463	-5.124038	1.243996
H	2.046975	3.083512	-0.464628
H	-2.407263	2.880773	-0.201847
H	2.636634	-1.758862	0.558601
H	-2.503498	-2.042009	0.627754
H	4.605793	-0.335221	0.084764
H	-4.605559	-0.792991	0.485341
H	-0.86741	-2.235065	2.630264
H	1.203435	-5.282878	-0.683055
H	-0.705491	-4.658589	3.110669
H	0.322822	-6.185774	1.450894
H	5.262572	1.895378	-0.986824
H	4.398101	3.251249	-0.560467
H	-4.835103	2.775548	-0.078124
H	-5.681506	1.285589	0.209277
H	1.075161	-2.856101	-1.148814
O	4.007118	0.51601	2.158825
H	4.270334	-0.384414	2.410556
H	5.1218	2.31449	0.611412

RDA-H2A-C8'-TS-H

Atom	X	Y	Z		
O	-0.093716	1.858879	0.033192	Electronic Energy (EE)	-993.684
N	4.625663	2.212248	-0.531887	Zero-point Energy Correction	0.323505
N	-4.778476	1.787755	0.094876	Thermal Correction to Energy	0.342745
C	0.008468	-0.913508	0.527896	Thermal Correction to Enthalpy	0.343689
C	1.225257	-0.088479	0.562426	Thermal Correction to Free Energy	0.275592
C	-1.193313	-0.282215	0.357298		

C	1.106795	1.261237	0.102237
C	-1.238526	1.148826	0.171982
C	3.44267	1.430469	-0.180455
C	-3.628885	1.143825	0.160856
C	0.111832	-2.369222	0.761021
C	2.210494	2.036459	-0.22536
C	-2.401832	1.849285	0.089554
C	2.540418	-0.67189	0.503542
C	-2.465635	-0.968835	0.361051
C	3.628649	0.082485	0.172614
C	-3.628627	-0.293348	0.277981
C	0.769899	-3.185223	-0.163947
C	-0.455935	-2.930064	1.908192
C	0.845757	-4.555432	0.054469
C	-0.35904	-4.299523	2.12923
C	0.286598	-5.11265	1.202172
H	2.083057	3.071777	-0.51797
H	-2.389016	2.923119	-0.048965
H	2.661753	-1.70896	0.784995
H	-2.471015	-2.048331	0.437036
H	4.624585	-0.343284	0.170382
H	-4.582502	-0.807116	0.288215
H	-0.95949	-2.290217	2.624429
H	1.343219	-5.187241	-0.671536
H	-0.791705	-4.729938	3.024339
H	0.354536	-6.180498	1.373383
H	5.118482	1.791665	-1.330798
H	4.385331	3.178486	-0.781714
H	-4.807408	2.795166	-0.00134
H	-5.656133	1.284281	0.132173
H	1.204702	-2.747307	-1.05583
H	5.289543	2.244923	0.252852
O	1.065644	0.28982	2.419834
H	1.979743	0.545025	2.627858

RDA-H2A-C8-TS-H

Atom	X	Y	Z		
O	-0.126429	1.800858	-0.077202	Electronic Energy (EE)	-993.69
N	4.617567	2.269483	-0.247582	Zero-point Energy Correction	0.323016
N	-4.819952	1.791273	0.072445	Thermal Correction to Energy	0.342518
C	-0.019607	-0.949765	0.420084	Thermal Correction to Enthalpy	0.343462
C	1.17929	-0.181049	0.251376	Thermal Correction to Free Energy	0.274327
C	-1.238846	-0.302977	0.330731		
C	1.070235	1.186192	0.009211		
C	-1.275985	1.112375	0.08368		

C	3.432071	1.429742	-0.071478
C	-3.664051	1.143567	0.140936
C	0.067166	-2.399235	0.705607
C	2.190195	2.01229	-0.157272
C	-2.439698	1.825018	-0.006014
C	2.491185	-0.723791	0.428535
C	-2.508939	-0.967742	0.441731
C	3.614616	0.076822	0.191633
C	-3.668749	-0.280545	0.355256
C	0.643871	-3.263757	-0.229237
C	-0.427881	-2.902611	1.911327
C	0.710419	-4.625886	0.038355
C	-0.339204	-4.263762	2.180695
C	0.224094	-5.125899	1.243987
H	2.059765	3.070627	-0.350913
H	-2.41462	2.891757	-0.18958
H	2.618094	-1.795052	0.487302
H	-2.525721	-2.040105	0.585545
H	4.610586	-0.345881	0.246901
H	-4.624958	-0.784196	0.433605
H	-0.867861	-2.225714	2.635366
H	1.144167	-5.295934	-0.694195
H	-0.714181	-4.649739	3.12094
H	0.284672	-6.187431	1.453097
H	5.311626	1.808409	-0.848649
H	4.382185	3.172694	-0.674554
H	-4.847699	2.788977	-0.090926
H	-5.695794	1.296131	0.173403
H	1.023691	-2.868781	-1.165278
O	2.567032	-0.557504	2.471236
H	3.477507	-0.868967	2.610179
H	5.070886	2.458027	0.655646

RDA-H2A-C9-TS-H

Atom	X	Y	Z		
O	-0.08675	1.78336	-0.127366	Electronic Energy (EE)	-993.69
N	4.656312	2.171408	-0.508894	Zero-point Energy Correction	0.324338
N	-4.777745	1.736558	-0.042326	Thermal Correction to Energy	0.343296
C	0.039998	-0.905512	0.716202	Thermal Correction to Enthalpy	0.34424
C	1.245657	-0.154684	0.405997	Thermal Correction to Free Energy	0.276803
C	-1.198442	-0.272379	0.512476		
C	1.12185	1.1743	-0.009316		
C	-1.233343	1.108547	0.103218		
C	3.468945	1.382215	-0.177303		
C	-3.625309	1.111372	0.116896		

C	0.128345	-2.383443	0.8725
C	2.229502	1.960023	-0.315158
C	-2.399802	1.789134	-0.084306
C	2.53961	-0.685935	0.554272
C	-2.461011	-0.940925	0.665678
C	3.652289	0.072745	0.26303
C	-3.625394	-0.284128	0.483094
C	0.69302	-3.103435	-0.187051
C	-0.377165	-3.067966	1.979251
C	0.747256	-4.491098	-0.13751
C	-0.305256	-4.456191	2.027981
C	0.253725	-5.170151	0.972675
H	2.100069	2.984738	-0.642161
H	-2.386148	2.826411	-0.394658
H	2.662301	-1.699871	0.911621
H	-2.466468	-1.989039	0.933651
H	4.651161	-0.33041	0.376095
H	-4.578559	-0.785887	0.599452
H	-0.816109	-2.518904	2.800928
H	1.179172	-5.038848	-0.96642
H	-0.690079	-4.978664	2.895632
H	0.304326	-6.251768	1.014619
H	5.100336	1.819386	-1.366725
H	4.429186	3.161225	-0.655601
H	-4.807605	2.713986	-0.306452
H	-5.654424	1.248152	0.093081
H	1.076357	-2.578867	-1.055207
O	-0.273245	-0.300867	2.755563
H	-0.150339	0.663616	2.701327
H	5.355727	2.115146	0.241172

RDA-HA-C1-TS-H

Atom	X	Y	Z		
O	-0.123713	1.803698	-0.036676	Electronic Energy (EE)	-993.264
N	4.560639	2.224219	-0.226145	Zero-point Energy Correction	0.307901
N	-4.835095	1.805565	0.048331	Thermal Correction to Energy	0.3274
C	0.005473	-0.943448	0.429096	Thermal Correction to Enthalpy	0.328345
C	1.1746	-0.192018	0.269948	Thermal Correction to Free Energy	0.259752
C	-1.243441	-0.297183	0.337776		
C	1.080729	1.201621	0.037577		
C	-1.277689	1.101628	0.107008		
C	3.463893	1.474433	-0.060856		
C	-3.670545	1.144561	0.131403		
C	0.077956	-2.39829	0.705408		
C	2.1798	2.025826	-0.123286		

C	-2.446194	1.820423	0.008153
C	2.50186	-0.725912	0.428182
C	-2.501507	-0.957521	0.434709
C	3.610894	0.071068	0.181154
C	-3.672008	-0.268867	0.339193
C	0.630117	-3.266816	-0.239277
C	-0.407773	-2.90212	1.914685
C	0.687691	-4.63118	0.023188
C	-0.330526	-4.265119	2.178904
C	0.212158	-5.130762	1.23285
H	2.036295	3.085222	-0.295723
H	-2.412637	2.888645	-0.167117
H	2.632566	-1.796633	0.491416
H	-2.522175	-2.030347	0.578462
H	4.607271	-0.353647	0.218798
H	-4.624358	-0.780922	0.411017
H	-0.833783	-2.224146	2.646155
H	1.10612	-5.302804	-0.716952
H	-0.698345	-4.650224	3.12246
H	0.26409	-6.193579	1.437895
H	5.47943	1.808341	-0.176421
H	4.485733	3.212699	-0.419275
H	-4.854416	2.800984	-0.117449
H	-5.712149	1.312154	0.124599
H	1.001711	-2.872376	-1.178769
O	2.747784	-0.474909	2.445069
H	3.654325	-0.806009	2.556637

RDA-HA-C11-TS-H

Atom	X	Y	Z		
O	-0.157534	1.71836	-0.2165	Electronic Energy (EE)	-993.255
N	4.532557	2.024524	-0.316614	Zero-point Energy Correction	0.308489
N	-4.826905	1.991423	0.249335	Thermal Correction to Energy	0.32754
C	-0.11419	-1.021495	0.328182	Thermal Correction to Enthalpy	0.328484
C	1.091962	-0.299787	0.20214	Thermal Correction to Free Energy	0.261966
C	-1.335078	-0.31629	0.326616		
C	1.033269	1.090282	-0.095741		
C	-1.319787	1.079435	0.05288		
C	3.419282	1.30097	-0.135829		
C	-3.697371	1.26938	0.271774		
C	-0.064714	-2.508515	0.508562		
C	2.148345	1.873396	-0.287892		
C	-2.453164	1.859311	0.009431		
C	2.403519	-0.833033	0.404377		
C	-2.622268	-0.882123	0.587958		

C	3.519231	-0.072549	0.246689
C	-3.752263	-0.126217	0.574057
C	0.905317	-3.283504	-0.212337
C	-0.538731	-3.034174	1.752655
C	1.229998	-4.561036	0.196615
C	-0.189679	-4.30434	2.149925
C	0.680819	-5.080095	1.37015
H	2.033431	2.921617	-0.534794
H	-2.371537	2.914131	-0.221968
H	2.519097	-1.858274	0.728547
H	-2.70055	-1.935695	0.804904
H	4.503028	-0.492469	0.420886
H	-4.716562	-0.572889	0.786496
H	-1.166542	-2.417446	2.383759
H	1.919311	-5.153531	-0.391695
H	-0.572866	-4.69856	3.083169
H	0.944908	-6.079717	1.693718
H	5.442281	1.605812	-0.190637
H	4.479964	2.998975	-0.575046
H	-4.80806	2.974118	0.019175
H	-5.719092	1.552601	0.422519
H	1.32284	-2.869604	-1.121979
O	-1.422381	-2.981075	-0.817432
H	-1.319218	-3.945787	-0.822547

RDA-HA-C12-TS-H

Atom	X	Y	Z		
O	-0.102626	1.817225	-0.05459	Electronic Energy (EE)	-993.265
N	4.583232	2.228228	-0.263469	Zero-point Energy Correction	0.307468
N	-4.810953	1.790622	0.078819	Thermal Correction to Energy	0.327219
C	0.042845	-0.927204	0.428362	Thermal Correction to Enthalpy	0.328163
C	1.217408	-0.177825	0.25349	Thermal Correction to Free Energy	0.258782
C	-1.205436	-0.285433	0.35587		
C	1.111128	1.218465	0.006355		
C	-1.24814	1.112665	0.110131		
C	3.490012	1.471995	-0.095941		
C	-3.642116	1.139377	0.158288		
C	0.11574	-2.38078	0.698595		
C	2.204768	2.032801	-0.171741		
C	-2.423308	1.821564	0.01606		
C	2.537478	-0.712678	0.344968		
C	-2.46042	-0.95308	0.471		
C	3.632596	0.074627	0.174654		
C	-3.634644	-0.273202	0.381461		
C	0.655518	-3.253938	-0.228137		

C	-0.326897	-2.870424	1.958991
C	0.701397	-4.625382	0.046064
C	-0.324491	-4.264913	2.191748
C	0.207667	-5.128209	1.250269
H	2.068803	3.090892	-0.358677
H	-2.399436	2.887862	-0.172031
H	2.663409	-1.765188	0.563902
H	-2.475406	-2.025392	0.623128
H	4.631629	-0.338502	0.248894
H	-4.58356	-0.789236	0.467618
H	-0.903927	-2.220884	2.605056
H	1.115114	-5.300916	-0.692978
H	-0.719777	-4.640636	3.128008
H	0.23117	-6.193952	1.441656
H	5.503953	1.819046	-0.201596
H	4.504493	3.217338	-0.449799
H	-4.839049	2.784332	-0.096753
H	-5.683885	1.291875	0.168373
H	1.02352	-2.875864	-1.175178
O	1.377902	-2.390216	2.992333
H	1.159446	-2.89782	3.79042

RDA-HA-C13-TS-H

Atom	X	Y	Z		
				Electronic Energy (EE)	-993.264
O	-0.107286	1.823153	-0.097119	Zero-point Energy Correction	0.307587
N	4.581193	2.220753	-0.299562	Thermal Correction to Energy	0.327176
N	-4.813978	1.813505	0.043541	Thermal Correction to Enthalpy	0.32812
C	0.031941	-0.909467	0.453309	Thermal Correction to Free Energy	0.259169
C	1.207896	-0.163597	0.26868		
C	-1.214541	-0.269341	0.359498		
C	1.105496	1.224697	-0.016095		
C	-1.253774	1.124617	0.084572		
C	3.485274	1.471456	-0.115303		
C	-3.647716	1.159848	0.135064		
C	0.11021	-2.361977	0.745748		
C	2.201663	2.032225	-0.211749		
C	-2.426624	1.834883	-0.022556		
C	2.525919	-0.69746	0.383539		
C	-2.472471	-0.929885	0.489148		
C	3.623966	0.082477	0.196477		
C	-3.644306	-0.248005	0.386458		
C	0.650568	-3.240546	-0.210825		
C	-0.34901	-2.854545	1.952623		
C	0.698566	-4.610984	0.035233		
C	-0.211483	-4.232717	2.252702		

C	0.248904	-5.113452	1.243997
H	2.068344	3.08526	-0.42699
H	-2.399472	2.897109	-0.232065
H	2.650313	-1.743245	0.635847
H	-2.489623	-1.997871	0.665449
H	4.621738	-0.329961	0.28945
H	-4.594809	-0.759052	0.48407
H	-0.770352	-2.184735	2.693708
H	1.081076	-5.279097	-0.726361
H	-0.726372	-4.634358	3.115141
H	0.276131	-6.178187	1.443541
H	5.500677	1.811802	-0.221021
H	4.505565	3.204766	-0.511862
H	-4.838494	2.803937	-0.150298
H	-5.68866	1.320376	0.146164
H	1.007161	-2.843638	-1.154906
O	1.523941	-4.098186	3.313777
H	1.698472	-5.052311	3.340836

RDA-HA-C14-TS-H

Atom	X	Y	Z		
O	-0.100522	1.799308	-0.07744	Electronic Energy (EE)	-993.264
N	4.58905	2.17694	-0.267609	Zero-point Energy Correction	0.307618
N	-4.807925	1.811726	0.037605	Thermal Correction to Energy	0.327222
C	0.023682	-0.935317	0.46417	Thermal Correction to Enthalpy	0.328166
C	1.204576	-0.195806	0.283263	Thermal Correction to Free Energy	0.259227
C	-1.220044	-0.288556	0.368048		
C	1.108959	1.194649	0.004106		
C	-1.251258	1.105792	0.096259		
C	3.490009	1.431616	-0.08843		
C	-3.645262	1.152167	0.132921		
C	0.09122	-2.385666	0.75898		
C	2.208887	1.997998	-0.185945		
C	-2.420199	1.821904	-0.015141		
C	2.520504	-0.735217	0.400551		
C	-2.48154	-0.944204	0.486314		
C	3.622002	0.040892	0.219093		
C	-3.649684	-0.256759	0.378515		
C	0.644049	-3.264314	-0.180783		
C	-0.412114	-2.873499	1.978365		
C	0.708447	-4.61689	0.096208		
C	-0.330523	-4.216793	2.268858		
C	0.290923	-5.107067	1.357188		
H	2.08029	3.052244	-0.398081		
H	-2.386632	2.884599	-0.221275		

H	2.640407	-1.78234	0.648855
H	-2.505918	-2.013106	0.656778
H	4.617794	-0.376094	0.313101
H	-4.60298	-0.764149	0.467571
H	-0.859008	-2.183238	2.684803
H	1.113337	-5.305832	-0.63555
H	-0.709451	-4.597292	3.209398
H	0.185031	-6.173749	1.503128
H	5.50682	1.764018	-0.189185
H	4.517714	3.161678	-0.478262
H	-4.826566	2.803114	-0.151967
H	-5.685579	1.32227	0.132233
H	0.999594	-2.879516	-1.129745
O	2.139992	-5.146332	2.239639
H	2.600983	-5.554011	1.48935

RDA-HA-C2-TS-H

Atom	X	Y	Z	Electronic Energy (EE)	-993.27
O	-0.092227	1.811705	-0.078014	Zero-point Energy Correction	0.30786
N	4.583609	2.218572	-0.369329	Thermal Correction to Energy	0.327394
N	-4.7906	1.795551	0.131518	Thermal Correction to Enthalpy	0.328338
C	0.047204	-0.937838	0.427001	Thermal Correction to Free Energy	0.259453
C	1.228916	-0.180683	0.242985		
C	-1.192415	-0.293457	0.359438		
C	1.121577	1.216111	-0.024857		
C	-1.23581	1.108652	0.109209		
C	3.495483	1.479844	-0.156682		
C	-3.627971	1.140552	0.193728		
C	0.128587	-2.393363	0.691818		
C	2.207494	2.031201	-0.233947		
C	-2.406666	1.819653	0.031188		
C	2.532441	-0.712174	0.334672		
C	-2.452569	-0.956698	0.489396		
C	3.64713	0.092396	0.199443		
C	-3.623633	-0.273864	0.415932		
C	0.692819	-3.248054	-0.259181		
C	-0.360816	-2.914944	1.892217		
C	0.755696	-4.614897	-0.012823		
C	-0.276987	-4.280302	2.141059		
C	0.275832	-5.13158	1.18797		
H	2.063919	3.081945	-0.4529		
H	-2.383524	2.885331	-0.160166		
H	2.668686	-1.763086	0.555429		
H	-2.467486	-2.028238	0.641145		
H	4.637703	-0.342317	0.171103		

H	-4.573973	-0.785262	0.512682
H	-0.794054	-2.248402	2.629821
H	1.182168	-5.275265	-0.758435
H	-0.647451	-4.678291	3.078184
H	0.332729	-6.196427	1.380752
H	5.50431	1.809062	-0.295903
H	4.507391	3.202489	-0.586236
H	-4.816486	2.790508	-0.040612
H	-5.665227	1.30225	0.239061
H	1.067708	-2.841147	-1.191875
O	3.905028	0.613724	2.313558
H	3.678935	-0.269994	2.646044

RDA-HA-C3-TS-H

Atom	X	Y	Z		
O	-0.106819	1.807843	-0.022018	Electronic Energy (EE)	-993.262
N	4.567709	2.1267	-0.526391	Zero-point Energy Correction	0.308309
N	-4.809239	1.779204	0.085691	Thermal Correction to Energy	0.327473
C	0.033603	-0.94279	0.450567	Thermal Correction to Enthalpy	0.328418
C	1.213587	-0.182675	0.286649	Thermal Correction to Free Energy	0.260824
C	-1.207203	-0.298389	0.369349		
C	1.09968	1.199731	0.045169		
C	-1.25181	1.108204	0.129496		
C	3.514486	1.467931	-0.018497		
C	-3.643011	1.133578	0.165965		
C	0.109307	-2.401947	0.701781		
C	2.198973	2.023379	-0.13001		
C	-2.423638	1.817639	0.03229		
C	2.545567	-0.729208	0.38533		
C	-2.467133	-0.966321	0.472574		
C	3.640356	0.042062	0.232078		
C	-3.636707	-0.284486	0.38021		
C	0.674016	-3.247914	-0.256275		
C	-0.388266	-2.934629	1.893507		
C	0.72933	-4.617798	-0.025635		
C	-0.313529	-4.303531	2.126409		
C	0.24	-5.146232	1.166323		
H	2.067864	3.073043	-0.360854		
H	-2.398734	2.884833	-0.149572		
H	2.658034	-1.785697	0.590004		
H	-2.482427	-2.038636	0.617343		
H	4.639401	-0.371624	0.301029		
H	-4.587593	-0.798743	0.455339		
H	-0.822764	-2.274696	2.636377		
H	1.156767	-5.271447	-0.776593		

H	-0.691872	-4.71076	3.056383
H	0.290144	-6.213596	1.346607
H	5.491347	1.757347	-0.343785
H	4.497337	3.128565	-0.647192
H	-4.840083	2.775193	-0.080244
H	-5.681804	1.278417	0.17485
H	1.056579	-2.831827	-1.181935
O	3.371714	2.173118	1.849167
H	2.770989	1.583383	2.329729

RDA-HA-C4'-TS-H

Atom	X	Y	Z		
O	-0.100433	1.770445	-0.160152	Electronic Energy (EE)	-993.262
N	4.582986	2.194152	-0.265016	Zero-point Energy Correction	0.308597
N	-4.81126	1.797978	0.079895	Thermal Correction to Energy	0.327655
C	0.04493	-0.953029	0.466214	Thermal Correction to Enthalpy	0.328599
C	1.206384	-0.206115	0.305972	Thermal Correction to Free Energy	0.261413
C	-1.21107	-0.29676	0.40673		
C	1.087646	1.216981	0.132822		
C	-1.253498	1.082315	0.093935		
C	3.508872	1.441213	-0.059191		
C	-3.645982	1.134171	0.170798		
C	0.116109	-2.417232	0.694718		
C	2.217726	2.017601	-0.146043		
C	-2.422508	1.795233	-0.028686		
C	2.532126	-0.724322	0.411947		
C	-2.465813	-0.944502	0.569976		
C	3.637544	0.051706	0.244931		
C	-3.641299	-0.25975	0.466336		
C	0.646868	-3.25434	-0.289385		
C	-0.341228	-2.961903	1.897281		
C	0.709951	-4.627114	-0.074208		
C	-0.260898	-4.333144	2.113402		
C	0.259838	-5.167362	1.127292		
H	2.083012	3.062343	-0.399612		
H	-2.390804	2.849643	-0.274449		
H	2.658174	-1.776569	0.636104		
H	-2.487345	-2.006805	0.777271		
H	4.632311	-0.367791	0.331491		
H	-4.590045	-0.766607	0.597824		
H	-0.747761	-2.309441	2.662257		
H	1.113574	-5.272915	-0.844978		
H	-0.607539	-4.749246	3.051867		
H	0.314951	-6.236456	1.29557		
H	5.509054	1.788172	-0.227701		

H	4.495023	3.179848	-0.47583
H	-4.829985	2.781147	-0.146244
H	-5.687514	1.316863	0.215853
H	0.999294	-2.828651	-1.222535
O	1.366369	1.854279	1.97266
H	1.095054	2.783815	1.900733

RDA-HA-C4-TS-H

Atom	X	Y	Z
O	-0.086223	1.814787	-0.070222
N	4.590299	2.211952	-0.367453
N	-4.790932	1.814979	0.097545
C	0.049497	-0.928292	0.432427
C	1.226378	-0.175049	0.242308
C	-1.19397	-0.28107	0.36263
C	1.115999	1.209319	-0.013009
C	-1.234786	1.117641	0.108834
C	3.510557	1.462055	-0.153205
C	-3.626242	1.158942	0.172482
C	0.125645	-2.383009	0.703296
C	2.219458	2.03768	-0.173474
C	-2.404389	1.833693	0.018061
C	2.551306	-0.707368	0.330289
C	-2.45439	-0.940228	0.487124
C	3.649217	0.069361	0.137535
C	-3.623956	-0.253526	0.401189
C	0.695123	-3.242918	-0.239722
C	-0.373792	-2.898381	1.902293
C	0.753289	-4.608846	0.01295
C	-0.294628	-4.262765	2.157728
C	0.26347	-5.119274	1.212407
H	2.079624	3.078473	-0.436656
H	-2.374841	2.898596	-0.176708
H	2.677261	-1.756365	0.565877
H	-2.474123	-2.011101	0.642894
H	4.646427	-0.349261	0.201738
H	-4.575417	-0.763969	0.492899
H	-0.81098	-2.2278	2.633887
H	1.184175	-5.273362	-0.726401
H	-0.672708	-4.655943	3.09384
H	0.316768	-6.183374	1.410284
H	5.514279	1.805306	-0.323647
H	4.50505	3.19992	-0.5622
H	-4.815205	2.8081	-0.082878
H	-5.665983	1.321349	0.196109

Electronic Energy (EE)	-993.273
Zero-point Energy Correction	0.307811
Thermal Correction to Energy	0.327364
Thermal Correction to Enthalpy	0.328308
Thermal Correction to Free Energy	0.259387

H	1.078083	-2.840727	-1.171221
O	2.393407	2.55634	2.087138
H	1.486824	2.905639	2.069002

RDA-HA-C9-TS-H

Atom	X	Y	Z
O	-0.106624	1.819876	0.000997
N	4.558271	2.162486	-0.455491
N	-4.819444	1.737603	-0.082509
C	0.050195	-0.901676	0.707971
C	1.234796	-0.143916	0.449441
C	-1.208096	-0.280121	0.477687
C	1.109893	1.23095	0.068775
C	-1.253327	1.094283	0.153784
C	3.4841	1.440538	-0.164725
C	-3.649616	1.096738	0.089734
C	0.124052	-2.380335	0.856669
C	2.192347	2.010524	-0.227185
C	-2.429315	1.782425	-0.034642
C	2.557126	-0.689172	0.515358
C	-2.453449	-0.9519	0.575297
C	3.641625	0.064445	0.220434
C	-3.636053	-0.294816	0.389412
C	0.649924	-3.128102	-0.199705
C	-0.380971	-3.030063	1.983441
C	0.67204	-4.516979	-0.125655
C	-0.347359	-4.418164	2.056079
C	0.177418	-5.163385	1.003088
H	2.05944	3.046079	-0.515134
H	-2.404783	2.836304	-0.283766
H	2.678962	-1.718609	0.826341
H	-2.464657	-2.011836	0.795783
H	4.643426	-0.344496	0.274932
H	-4.57986	-0.821837	0.463779
H	-0.788971	-2.445901	2.798825
H	1.078283	-5.091037	-0.94987
H	-0.732921	-4.917418	2.937066
H	0.200449	-6.24518	1.062595
H	5.48197	1.751448	-0.410554
H	4.475247	3.135053	-0.722872
H	-4.841789	2.718662	-0.316855
H	-5.692896	1.23761	-0.012707
H	1.032372	-2.62354	-1.080756
O	0.634683	-0.324424	2.647892
H	0.248086	0.566025	2.699247

Electronic Energy (EE)	-993.262
Zero-point Energy Correction	0.308948
Thermal Correction to Energy	0.327951
Thermal Correction to Enthalpy	0.328895
Thermal Correction to Free Energy	0.261974

RDA-HA-N17-TS-H

Atom	X	Y	Z		
O	-0.119767	1.833037	-0.07543	Electronic Energy (EE)	-993.268
N	4.558098	2.21373	-0.319536	Zero-point Energy Correction	0.305377
N	-4.81099	1.768354	0.129849	Thermal Correction to Energy	0.324559
C	0.037022	-0.923611	0.431379	Thermal Correction to Enthalpy	0.325503
C	1.209094	-0.161421	0.246172	Thermal Correction to Free Energy	0.256557
C	-1.206331	-0.281107	0.360606		
C	1.097077	1.236892	-0.018302		
C	-1.258396	1.126865	0.10883		
C	3.47859	1.476252	-0.134807		
C	-3.649074	1.129928	0.19274		
C	0.120851	-2.380337	0.696203		
C	2.182336	2.043495	-0.213683		
C	-2.431447	1.824904	0.030314		
C	2.529605	-0.701934	0.344719		
C	-2.460594	-0.959659	0.491059		
C	3.624908	0.075663	0.160225		
C	-3.635515	-0.289315	0.416612		
C	0.676589	-3.233792	-0.260271		
C	-0.358654	-2.902101	1.900084		
C	0.74184	-4.600835	-0.015439		
C	-0.274501	-4.26797	2.14654		
C	0.270686	-5.118409	1.188394		
H	2.052543	3.100002	-0.414032		
H	-2.422032	2.890827	-0.160697		
H	2.64978	-1.750941	0.580821		
H	-2.463078	-2.030825	0.643778		
H	4.625361	-0.33282	0.238085		
H	-4.582898	-0.806175	0.512617		
H	-0.785997	-2.236131	2.641787		
H	1.162876	-5.260468	-0.764746		
H	-0.638342	-4.666804	3.085875		
H	0.328429	-6.183481	1.379601		
H	5.558322	1.771287	-0.257706		
H	4.478986	3.203937	-0.516365		
H	-4.848339	2.764483	-0.044334		
H	-5.682528	1.265537	0.235818		
H	1.044357	-2.825939	-1.195516		
O	6.772458	1.169805	-0.851825		
H	7.287559	1.704	-1.472607		

RDA-N17-TS-H

Atom	X	Y	Z	Electronic Energy (EE)	-992.796
O	-0.086245	1.741384	-0.053291	Zero-point Energy Correction	0.294996
N	4.656126	2.029853	-0.236701	Thermal Correction to Energy	0.314112
N	-4.787069	1.812433	0.132614	Thermal Correction to Enthalpy	0.315056
C	0.006504	-1.014733	0.415446	Thermal Correction to Free Energy	0.246522
C	1.1999	-0.279155	0.257022		
C	-1.225032	-0.346178	0.343393		
C	1.119485	1.117423	0.010892		
C	-1.241103	1.059763	0.115223		
C	3.521982	1.333012	-0.076639		
C	-3.633785	1.135281	0.186502		
C	0.055998	-2.476595	0.657815		
C	2.231044	1.909248	-0.154885		
C	-2.401696	1.79302	0.041874		
C	2.508326	-0.840067	0.370396		
C	-2.496328	-0.988268	0.453033		
C	3.619215	-0.070766	0.210108		
C	-3.655281	-0.282628	0.382912		
C	0.611102	-3.326131	-0.302928		
C	-0.452023	-3.009622	1.845182		
C	0.646825	-4.697986	-0.079858		
C	-0.396381	-4.380589	2.071263		
C	0.147833	-5.226079	1.108162		
H	2.112665	2.971708	-0.334465		
H	-2.356974	2.861207	-0.130832		
H	2.607775	-1.893835	0.59637		
H	-2.531985	-2.061638	0.587121		
H	4.609256	-0.501859	0.300201		
H	-4.615102	-0.77902	0.464866		
H	-0.87812	-2.347877	2.591305		
H	1.067664	-5.353293	-0.833154		
H	-0.781528	-4.787366	2.998674		
H	0.18353	-6.294903	1.283256		
H	4.424895	3.002896	-0.434132		
H	-4.794809	2.810025	-0.023477		
H	-5.670231	1.331995	0.224671		
H	1.002534	-2.910262	-1.224859		
O	4.706396	2.967891	1.914698		
H	4.682611	2.042806	2.181177		

RDA-N17-TS-M

Atom	X	Y	Z	Electronic Energy (EE)	-993.274
O	-0.119767	1.833037	-0.07543	Zero-point Energy Correction	0.305385
N	4.558098	2.21373	-0.319536	Thermal Correction to Energy	0.324601
N	-4.81099	1.768354	0.129849	Thermal Correction to Enthalpy	0.325545

C	0.037022	-0.923611	0.431379	Thermal Correction to Free Energy	0.25652
C	1.209094	-0.161421	0.246172		
C	-1.206331	-0.281107	0.360606		
C	1.097077	1.236892	-0.018302		
C	-1.258396	1.126865	0.10883		
C	3.47859	1.476252	-0.134807		
C	-3.649074	1.129928	0.19274		
C	0.120851	-2.380337	0.696203		
C	2.182336	2.043495	-0.213683		
C	-2.431447	1.824904	0.030314		
C	2.529605	-0.701934	0.344719		
C	-2.460594	-0.959659	0.491059		
C	3.624908	0.075663	0.160225		
C	-3.635515	-0.289315	0.416612		
C	0.676589	-3.233792	-0.260271		
C	-0.358654	-2.902101	1.900084		
C	0.74184	-4.600835	-0.015439		
C	-0.274501	-4.26797	2.14654		
C	0.270686	-5.118409	1.188394		
H	2.052543	3.100002	-0.414032		
H	-2.422032	2.890827	-0.160697		
H	2.64978	-1.750941	0.580821		
H	-2.463078	-2.030825	0.643778		
H	4.625361	-0.33282	0.238085		
H	-4.582898	-0.806175	0.512617		
H	-0.785997	-2.236131	2.641787		
H	1.162876	-5.260468	-0.764746		
H	-0.638342	-4.666804	3.085875		
H	0.328429	-6.183481	1.379601		
H	5.558322	1.771287	-0.257706		
H	4.478986	3.203937	-0.516365		
H	-4.848339	2.764483	-0.044334		
H	-5.682528	1.265537	0.235818		
H	1.044357	-2.825939	-1.195516		
O	6.772458	1.169805	-0.851825		
H	7.287559	1.704	-1.472607		

RDA-N17-TS-P

Atom	X	Y	Z		
				Electronic Energy (EE)	-993.251
O	-0.050818	1.822742	0.013024	Zero-point Energy Correction	0.304408
N	4.653058	2.242476	-0.002521	Thermal Correction to Energy	0.323897
N	-4.752731	1.800848	0.012623	Thermal Correction to Enthalpy	0.324841
C	0.067934	-0.931515	0.474203	Thermal Correction to Free Energy	0.255476
C	1.260632	-0.168092	0.34147		
C	-1.1629	-0.284529	0.362768		

C	1.15506	1.21982	0.101779
C	-1.195454	1.125895	0.133391
C	3.535784	1.469983	0.062587
C	-3.589624	1.152994	0.111407
C	0.139892	-2.38964	0.725673
C	2.261576	2.034552	-0.055795
C	-2.365019	1.836813	0.016522
C	2.569846	-0.709449	0.466629
C	-2.4285	-0.953241	0.418373
C	3.674438	0.078832	0.333841
C	-3.592378	-0.26941	0.302248
C	0.722016	-3.23452	-0.223352
C	-0.375733	-2.923043	1.90992
C	0.775098	-4.603866	0.008044
C	-0.299605	-4.291135	2.144473
C	0.269886	-5.132248	1.192894
H	2.130225	3.091417	-0.254505
H	-2.333882	2.905792	-0.154576
H	2.684603	-1.763338	0.683458
H	-2.446566	-2.028145	0.541969
H	4.670148	-0.336441	0.429912
H	-4.54462	-0.785454	0.337004
H	-0.820816	-2.263963	2.647437
H	1.214376	-5.257422	-0.736214
H	-0.688038	-4.699237	3.069972
H	0.320689	-6.199361	1.374854
H	5.584646	1.76674	-0.331013
H	4.516863	3.185277	-0.358958
H	-4.785521	2.799762	-0.132324
H	-5.630619	1.305158	0.071679
H	1.117372	-2.818886	-1.143944
O	6.278082	1.151258	-1.33834
H	6.31948	1.7375	-2.1098

References

(1) Evans, M. G.; Polanyi, M. Some Applications of the Transition State Method to the Calculation of Reaction Velocities, Especially in Solution. *Trans. Faraday Soc.* **1935**, *31*, 875-894.

(2) Eyring, H. The Activated Complex in Chemical Reactions. *J. Chem. Phys.* **1935**, *3*, 107-115.

(3) Truhlar, D. G.; Hase, W. L.; Hynes, J. T. Current Status of Transition-State Theory. *J. Phys. Chem.* **1983**, *87*, 2664-2682.

(4) Furuncuoglu, T.; Ugur, I.; Degirmenci, I.; Aviyente, V. Role of Chain Transfer Agents in Free Radical Polymerization Kinetics. *Macromolecules* **2010**, *43*, 1823-1835.

(5) Vélez, E.; Quijano, J.; Notario, R.; Pabón, E.; Murillo, J.; Leal, J.; Zapata, E.; Alarcón, G. A Computational Study of Stereospecifity in the Thermal Elimination Reaction of Menthyl Benzoate in the Gas Phase. *J. Phys. Org. Chem.* **2009**, *22*, 971-977.

(6) Pollak, E.; Pechukas, P. Symmetry Numbers, Not Statistical Factors, Should Be Used in Absolute Rate Theory and in Broensted Relations. *J. Am. Chem. Soc.* **1978**, *100*, 2984-2991.

(7) Fernández-Ramos, A.; Ellingson, B. A.; Meana-Pañeda, R.; Marques, J. M.; Truhlar, D. G. Symmetry Numbers and Chemical Reaction Rates. *Theor. Chem. Acc.* **2007**, *118*, 813-826.

(8) Eckart, C. The Penetration of a Potential Barrier by Electrons. *Phy. Rev.* **1930**, *35*, 1303.

(9) Marcus, R. A. Chemical and Electrochemical Electron-Transfer Theory. *Annu. Rev. Phys. Chem.* **1964**, *15*, 155-196.

(10) Marcus, R. A. Electron Transfer Reactions in Chemistry. Theory and Experiment. *Rev. Mod. Phys.* **1993**, *65*, 599.

(11) Lu, Y.; Wang, A.; Shi, P.; Zhang, H. A Theoretical Study on the Antioxidant Activity of Piceatannol and Isorhapontigenin Scavenging Nitric Oxide and Nitrogen Dioxide Radicals. *PloS one* **2017**, *12*, e0169773.

(12) Lu, Y.; Wang, A.; Shi, P.; Zhang, H.; Li, Z. Quantum Chemical Study on the Antioxidation Mechanism of Piceatannol and Isorhapontigenin toward Hydroxyl and Hydroperoxyl Radicals. *PloS one* **2015**, *10*, e0133259.

(13) Nelsen, S. F.; Blackstock, S. C.; Kim, Y. Estimation of Inner Shell Marcus Terms for Amino Nitrogen Compounds by Molecular Orbital Calculations. *J. Am. Chem. Soc.* **1987**, *109*, 677-682.

(14) Nelsen, S. F.; Weaver, M. N.; Luo, Y.; Pladziewicz, J. R.; Ausman, L. K.; Jentzsch, T. L.; O'Konek, J. J. Estimation of Electronic Coupling for Intermolecular Electron Transfer from Cross-Reaction Data. *J. Phys. Chem. A* **2006**, *110*, 11665-11676.

(15) Galano, A.; Alvarez-Idaboy, J. R. A Computational Methodology for Accurate Predictions of Rate Constants in Solution: Application to the Assessment of Primary Antioxidant Activity. *J. Comput. Chem.* **2013**, *34*, 2430-2445.

(16) Collins, F. C.; Kimball, G. E. Diffusion-Controlled Reaction Rates. *J. Colloid Sci.* **1949**, *4*, 425-437.

(17) Von Smoluchowski, M. Mathematical Theory of the Kinetics of the Coagulation of Colloidal Solutions. *Z. Phys. Chem* **1917**, *92*, 129-68.

(18) Truhlar, D. G. Nearly Encounter-Controlled Reactions: The Equivalence of the Steady-State and Diffusional Viewpoints. *J. Chem. Educ.* **1985**, *62*, 104.

(19) Einstein, A. On the Motion of Small Particles Suspended in Liquids at Rest Required by the Molecular-Kinetic Theory of Heat. *Ann. Phys.* **1905**, *17*, 549-560.

(20) Stokes, G. G., *Mathematical and Physical Papers*. University Press: Cambridge, 1905.
